# PAK4-targeting PROTAC inhibits bladder cancer cell proliferation

**DOI:** 10.1371/journal.pone.0358561

**Published:** 2026-09-21

**Authors:** Shan Xu, Hongjun Xie, Zhao Yang, Zhenkun Ma, Xiaojing Bai, Xiaoyu Feng, Jiale An, Bohan Ma

**Affiliations:** 1 Department of Urology, The First Affiliated Hospital of Xi’an Jiaotong University, Xi’an, Shaanxi, China; 2 Key Laboratory for Tumor Precision Medicine of Shaanxi Province, The First Affiliated Hospital of Xi’an Jiaotong University, Xi’an, Shaanxi, China; Noorda College of Osteopathic Medicine, UNITED STATES OF AMERICA

## Abstract

p21-Activated kinase 4 (PAK4) is widely dysregulated and acts as an oncogene in multiple cancers, and the inhibition or knockout of PAK4 has shown excellent therapeutic effects in inhibiting tumor progression in combination with immunotherapy across a variety of cancers. Previously, we reported our self-developed peptide proteolysis-targeting chimera (PROTAC) drug PpD, which specifically and selectively degrades the PAK4 protein to effectively inhibit the growth of renal cancer tumors. In this study, we confirmed that the PAK4 protein is highly expressed in bladder cancer (BLCA) tissues. Moreover, we established PAK4 knockout BLCA cell lines and explored the regulatory mechanisms of PAK4 in BLCA via RNA sequencing. Our RNA sequencing results revealed that PAK4 promotes the cell cycle progression of BLCA cells and inhibits apoptosis. Comparative studies of the PAK4 knockout cell lines T-24 sg/PAK4 and 5637 sg/PAK4 with PpD drug treatment revealed that both PAK4 knockout and PpD drug treatment inhibited the ERK1/2/CDK4 pathway, thereby blocking the cell cycle transition from the G1 phase to the S phase, and both induced caspase-3-mediated apoptotic cell death. *In vivo* animal experiments also confirmed that the growth of BLCA cells was significantly inhibited in both the PAK4 knockout group and the PpD drug-treated group. Collectively, our findings indicate that PpD exhibits potent anti-tumor activity against BLCA and holds promising potential for therapeutic application in other cancers.

## Introduction

Bladder cancer (BLCA) is one of the most common malignancies of the urinary system [1]. According to the GLOBOCAN 2024 update, which provides cancer incidence and mortality data for 2022, approximately 92,883 new cases and 41,367 deaths from BLCA occurred in 2022 (https://gco.iarc.fr/en). The incidence of BLCA has increased rapidly among both men and women in China [2]. Platinum chemotherapy has been the first-line treatment for advanced BLCA for 20 years [3–5]. However, due to side effects and drug resistance, platinum chemotherapy is not suitable for approximately 50% of patients [6], and their prognosis remains poor: the median progression-free survival is 8 months on average, and the overall survival is approximately 14 months on average [7,8]. In recent years, the rise of immunotherapy in the treatment of various cancers has provided new options for the treatment of advanced BLCA [9]. However, clinical trials have shown that more than 70% of patients with advanced BLCA do not have a significant response to immunotherapy [10,11]. Therefore, the development of new drugs with increased therapeutic prospects for new targets is urgently needed for the clinical treatment of advanced BLCA.

Continuously abnormal proliferation signals constitute a fundamental characteristic of malignant tumors and are intimately tied to their initiation, progression, invasion, and metastasis [12,13]. This aberrant proliferation of tumor cells heavily relies on dysregulation of cell cycle-related pathways [14,15]. Advancements in high-throughput sequencing technology have since revealed that cellular cycle abnormalities emerge as predominant drivers of tumorigenesis in more than 90% of BLCA tissues [16]. This finding underscores the notion that targeting the mechanisms governing the cell cycle in tumor cells could hold the key to identifying novel therapeutic targets for the treatment of BLCA.

p21-Activated kinase 4 (PAK4), which belongs to the serine/threonine (Ser/Thr) protein kinase family, is overexpressed in many human cancers and is related to poor prognosis, including in BLCA [17–20]. As an oncogene, PAK4 aberrantly phosphorylates substrates to upregulate specific oncogenic pathways, including the STAT3, ERK/MAPK, and PI3K/AKT pathways, resulting in growth factor independence, apoptosis inhibition, and metastasis [21,22]. In both *in vitro* and *in vivo* models, the genetic deletion of PAK4 inhibits tumor growth [23–25]. Abril-Rodríguez, Gabriel *et al.* reported that PAK4 inhibition enhances anti-PD-1 immunotherapy efficacy by reversing immune cell exclusion through increased infiltration of CD8 + T cells and CD103 + dendritic cells, mediated by remodeling of the extracellular matrix and upregulation of CCL21-CXCL10 chemokine signaling [26]. However, the immunomodulatory and immunotherapy-synergistic effects of PAK4 targeting in BLCA require further validation in immunocompetent preclinical models.

We designed a novel PAK4-targeting peptide degrader (PpD) that can degrade both wild-type PAK4^WT^ and mutant PAK4^E329K^ proteins to ultimately eliminate the PAK4 protein in renal cell carcinoma cells [27]. In this study, we validated PAK4 as a promising target in BLCA. PAK4 was overexpressed in BLCA compared with adjacent tissues, and the deletion of PAK4 in BLCA effectively inhibited tumor cell proliferation *in vitro* and *in vivo*. We verified the effect of PpD on PAK4 degradation and its therapeutic efficacy at both the cellular and animal levels in BLCA. In summary, our designed PAK4-targeted degradation drug, PpD, can degrade PAK4, highlighting the importance of selecting crucial targets for BLCA therapy.

## Materials and Methods

### Cell lines and cell culture

The human bladder cancer cell lines T-24 and 5637 were purchased from the American Type Culture Collection (ATCC, Manassas, VA, USA). The cell lines were grown in DMEM (Gibco-Thermo Fisher Scientific, Inc., Waltham, MA, USA) supplemented with 10% (v/v) fetal bovine serum (FBS; Gibco-Thermo Fisher Scientific, Inc., Waltham, MA, USA) at 37 °C and 5% CO_2_ in a humidified incubator.

### Western blotting

Briefly, total protein lysates were extracted from tumor cells and quantified via a BLCA protein assay (#23225, Thermo Scientific Inc., IL, USA). A total of 30 μg of protein was separated via 10% SDS‒PAGE (#P2012, NCM Biotech, Shanghai, China) and blotted onto a BioTrace™ NT nitrocellulose transfer membrane (#66485, Pall, Inc., USA). The membranes were blocked with 5% skim milk at room temperature for 1 h and then incubated at 4 °C overnight with primary antibodies, including primary antibodies against PAK4 (#1 Absin, abs146701, 1:1000, Shanghai, China; #2 Cell Signaling Technology, #62690, 1:1000, Shanghai, China), PARP (Cell Signaling Technology, #9532, 1:1000, Shanghai, China), CDK4 Cell Signaling Technology, #2906, 1:1000, Shanghai, China), Cyclin D1 (Proteintech, 60186–1-Ig, 1:1000, Wuhan, China), PCNA (Proteintech, 10205–2-AP, 1:1000, Wuhan, China), Erk1/2 (Abgent, #AM2189b, 1:1000, Suzhou, China), p-Erk1/2 (Cell Signaling Technology, #4370, 1:1000, Shanghai, China), caspase 3 (Cell Signaling Technology, #9662, 1:1000, Shanghai, China), antiVinculin (ABclonal, #A2752, 1:1000, Shanghai, China) and anti-β-actin (ABclonal, #A2752, 1:1000, Shanghai, China). For secondary antibodies, anti-mouse IgG (Beijing Zhongshan, #ZB-2305; 1:2000; Beijing, China) and anti-rabbit IgG (Beijing Zhongshan, #ZB2301; 1:2,000; Beijing, China) were utilized.

### Transfection and Establishment of PAK4 Knockout Stable Cloning Cells

T-24 and 5637 cells were transfected with packaging vectors (delta-8.9 and VSVG), and PAK4 knockout (KO) T-24 and 5637 cells were selected with puromycin. The sgRNA sequences for human PAK4 KO were 5’-CACCGGGACGAGTTTGAGAACATGT-3’ and 5’-AAACACATGTTCTCAAACTCGTCCC-3’. The PAK4 protein expression level in tumor cells was detected by western blotting. To establish CDK4-overexpressing BLCA cell models, T-24 and 5637 cells were transfected with HA-tagged human CDK4 plasmids (MiaoLing, Hubei, China) using Lipofectamine 2000 (#11668−019, Invitrogen, USA) according to the manufacturer’s protocol.

### Cell Viability Tests via Cell Counting Kit-8 (CCK-8)

CCK-8 was used to detect cell viability. T-24, 5637, T-24/sg-PAK4, and 5637/sg-PAK4 cells were seeded at a density of 5 × 10^3^ cells/well in 96-well plates, and three independent samples were seeded for each sample. After 24 hours, the samples were treated with or without the indicated concentrations (0, 31.3, 62.5, 125, 250, 500, 1000, 2000 nM) of the PpD drug. After 48 hours, the samples were incubated with CCK-8 reagent (#C6005, NCM Biotech, Suzhou, China) for 4 h, after which the absorbance values were measured at 450 nm. The cell viability rate was calculated as the average OD value in the PpD or sg group/average OD value in the control group × 100%.

### Cloning and Clonogenic Assay

Cells were seeded in a 6-well plate (1000 cells/well), cultured in DMEM containing the PpD drug (0, 125, 250, or 500 nM) for 7 days, and three independent samples were seeded for each sample. Crystal violet (#C0121, Beyotime, Shanghai, China) was used to stain the cells. The colony formation capacity of the cells was tested by 2-dimensional culture, and the number of clones was counted and plotted.

### Cell apoptosis assay

The human BLCA cell lines T-24 and 5737 were grown in 6-cm dishes. Overnight, the cells were treated with the PpD drug at the concentrations indicated in the figure legends for 48 h. The cells were harvested, washed, incubated with annexin V and propidium iodide (PI) for 30 min, and then analyzed using a FACSCalibur flow cytometer (BD Calibur). The data were analyzed via BD software and GraphPad Prism software.

### Datasets

The GEPIA2 (Gene Expression Profiling Interactive Analysis) website is a valuable and highly cited resource for gene expression analysis based on tumor and normal samples from the TCGA and GTEx databases (http://gepia2.cancer-pku.cn/#index). PAK4 was submitted to GEPIA2 to analyze PAK4 mRNA expression in BLCA. The expression of PAK4 was analyzed on the basis of pathological stage, and the results are shown in box plots and violin plots. The overall survival analyses and disease-free survival analysis of PAK4 are presented in the ‘Survival’ tab.

### KEGG and reactions pathway enrichment analysis

T-24/sg-control and T-24/sg-PAK4 cells were collected, and RNA extraction, library construction and sequencing were performed (Genedenovo Company, Guangzhou, China). Gene expression in T-24/sg-control and T-24/sg-PAK4 cells was compared, and the calculated *p* value was subjected to FDR correction. The differentially expressed genes (DEGs) were the genes whose FDR was ≤ 0.05 and whose expression threshold was |fold-change| > 2. DEGs were subjected to Kyoto Encyclopedia of Genes and Genomes (KEGG) pathway and Reactome enrichment analyses.

### Immunohistochemical analysis

We purchased tissue microarrays (#HBlaU079Su01) from Shanghai Outdo BioTeck Co. Ltd. (Shanghai, China). HBlaU079Su01 included 63 tumor tissues with BLCA and 16 adjacent bladder tissues. The GTVisionTM^+^ Detection System/Mo&Rb (#GK600510, Shanghai) was used to perform immunohistochemical (IHC) staining for PAK4 (absin, abs146701, 1:200, Shanghai). The percentage of stained cells was graded as follows: 0 for 0%, 1 for ≤ 25%, 2 for 25–50%, 3 for 50–75% and 4 for ≥ 75%. IHC intensity was scored as follows: 0 for no staining, 1 for weakly positive staining, 2 for moderately positive staining and 3 for strongly positive staining. The total score of each section was calculated by multiplying the intensity score by the percentage score.

### In Vivo Experimental Therapy in Mouse Models

The animal experimental procedures were conducted in accordance with institutional guidelines and were approved by the Laboratory Animal Center and Biomedical Ethics Committee of Xi’an Jiaotong University (approval number: 2020–256). All mice were raised in the Animal Centre of Xi’an Jiaotong University Health Science Centre in an SPF environment. In whole study, euthanasia was performed by cervical dislocation by trained personnel, and no anesthesia was required for our study as procedures did not involve surgery or cause significant pain. Mice were humanely euthanized by spinal dislocation immediately when mice tumor’s side length close to 2 cm. In our study, mice were monitored daily for signs of distress, pain, or illness. If mice exhibited severe morbidity or unexpected weight loss (>20% of baseline) will be removed from the study and euthanized spinal dislocation immediately. All handling and injections were performed by trained personnel using gentle restraint techniques to reduce stress. For the T-24-cell-derived xenograft therapy experiment, 20 BALB/c 4-week-old nude mice (female) were randomly separated into 4 groups (5 mice/group), and 2 × 10^6^ T-24 and 2 × 10^6^ T-24/sg-PAK4–10 cells were subcutaneously injected into the right shoulder as previously described. After 5 days, the mice were treated with control (PBS) or PpD (2.5 mg/kg) every 3 days via abdominal injection. Tumor size, tumor volume, and mouse body weight were measured every 2 days. Tumor volume was calculated using the following equation: tumor volume = length×width×0.5. To compare PpD and cisplatin in an *in vivo* experiment, 20 BALB/c 4-week-old nude mice (female) were randomly separated into 4 groups (5 mice/group), and 2 × 10^6^ T-24 cells were subcutaneously injected into the right shoulder as previously described. After 6 days, the mice were treated with control (PBS), or PpD (2.5 mg/kg), or cisplatin (2.5 mg/kg), or the combined regimen of PpD and cisplatin (alternating administration: PpD followed by cisplatin) every 2 days via abdominal injection. Tumor size, tumor volume, and mouse body weight were measured every 3 days. Tumor volume was calculated using the following equation: tumor volume = length×width×0.5.

### Ethics Statement

All experimental procedures involving animals were conducted in accordance with institutional guidelines and were approved by the Laboratory Animal Center and Biomedical Ethics Committee of Xi’an Jiaotong University (approval number: 2020−256). Informed consent was obtained from all individual participants included in the study from Shanghai Outdo BioTeck Co. Ltd. (approval number: YB M-0502).

### Statistical Analysis

GraphPad Prism version 6.0 software (GraphPad, USA) was used to perform one-way ANOVA (more than three groups), Pearson’s correlation analysis, linear regression analysis (IC50), and Student’s *t* test (two groups). *p* ≤ 0.05 was considered statistically significant.

## Results

### PAK4 was highly expressed in BLCA and predicted poor prognosis

**Fig 1:**
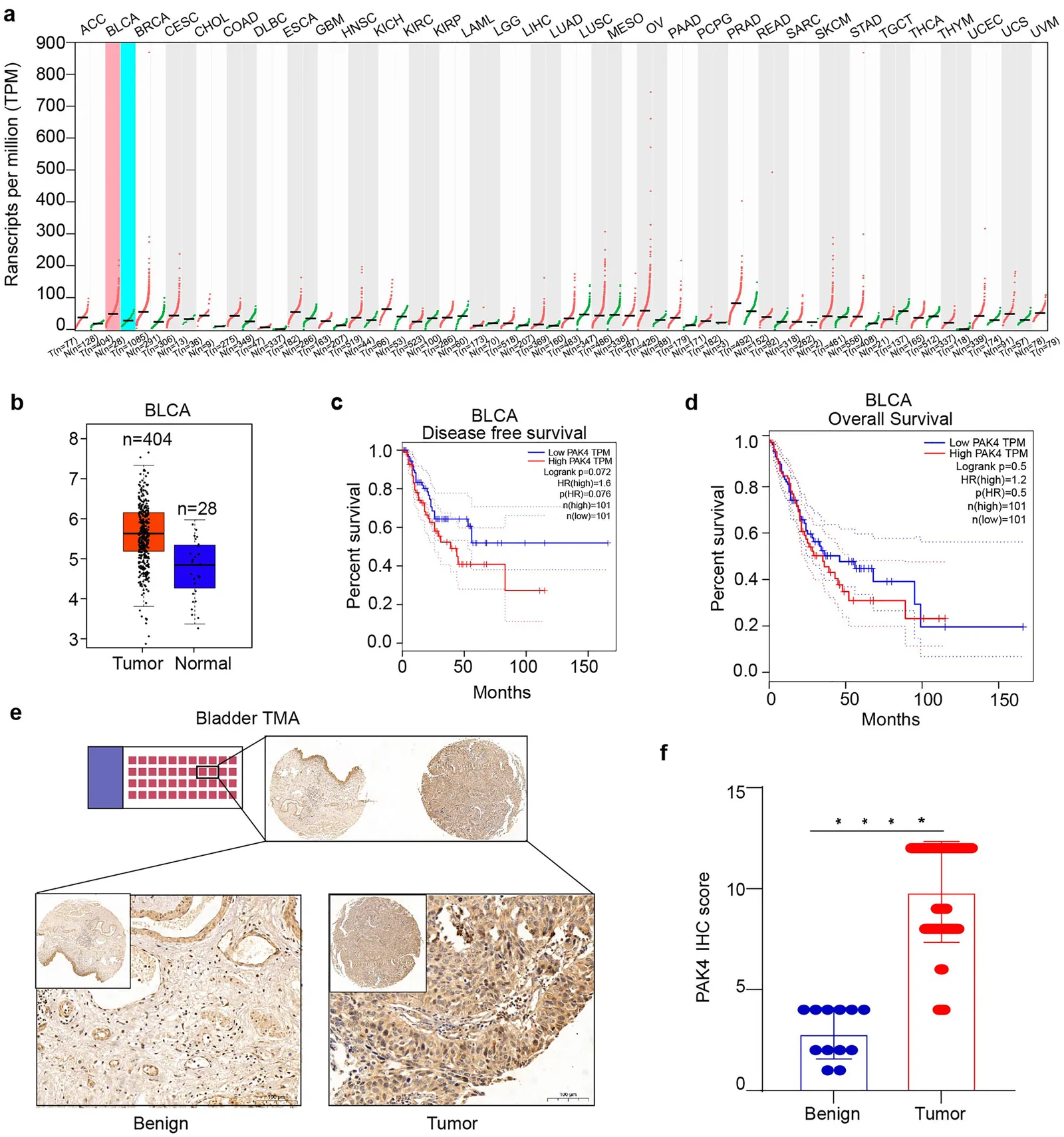
PAK4 is highly expressed and is a therapeutic target in BLCA. (a) PAK4 gene mRNA expression levels were analyzed via the GEPIA2 web resource among different types of tumors via analysis of The Cancer Genome Atlas (TCGA) database. ACC, adrenocortical carcinoma; BLCA, bladder urothelial carcinoma; BRCA, breast invasive carcinoma; CESC, cervical squamous cell carcinoma and endocervical adenocarcinoma; CHOL, cholangiocarcinoma; COAD, cholangiocarcinoma; ESCA, esophageal carcinoma; GBM, glioblastoma multiforme; HNSC, head and neck squamous cell carcinoma; KICH, kidney chromophobe; KIRC, kidney renal clear cell carcinoma; KIRP, kidney renal papillary cell carcinoma; LGG, brain lower grade glioma; LIHC, liver hepatocellular carcinoma; LUAD, lung adenocarcinoma; LUSC, lung squamous cell carcinoma; MESO, mesothelioma; OV, ovarian serous cystadenocarcinoma; PAAD, pancreatic adenocarcinoma; PCPG, pheochromocytoma and paraganglioma; PRAD, prostate adenocarcinoma; READ, rectum adenocarcinoma; SARC, sarcoma; SKCM, skin cutaneous melanoma; STAD, stomach adenocarcinoma; TGCT, testicular germ cell tumors; THCA, thyroid carcinoma; UCEC, uterine corpus endometrial carcinoma; UCS, uterine carcinosarcoma; UVM, uveal melanoma. (b) PAK4 mRNA levels were compared in tumor tissues from 404 patients with BLCA and 28 benign tissue samples from patients with BLCA. Statistical analysis was performed via Student’s t test to compare the two groups, with error bars representing ± standard deviation (SD). ** indicates p < 0.001. (c, d) A high level of PAK4 was related to poor disease-free survival (DFS) and overall survival (OS) in patients with BLCA according to the Cox proportional hazards model. (e) Representative immunohistochemistry (IHC) images of PAK4 expression in BLCA and benign tissues. Scale bar: 100 µm. (f) Statistical analysis of the differences in the IHC scores for PAK4 in tumors between BLCA and benign tissues. Statistical analysis was conducted via Student’s t test to compare the two groups, with error bars indicating ± SDs. **** represents p < 0.001.

We used GEPIA, a web tool for analyzing gene expression in cancers using TCGA & GTEx data, which includes differential expression, survival, and enrichment analyses with interactive visual imagery, to investigate the clinical relevance of PAK4 expression in patients with BLCA. As shown in Fig 1a, PAK4 is elevated in most tumor tissues compared with normal tissues. We further conducted comparative analyses of the PAK4 expression levels in tumor tissues and adjacent nontumor tissues in bladder urothelial carcinoma (BLCA)(Fig 1b). PAK4 expression was significantly greater in BLCA tissues than in adjacent noncancerous tissues (P < 0.01). To elucidate the correlation between PAK4 expression and the survival of patients with BLCA, we utilized the Cox proportional hazards model to evaluate the associations between PAK4 expression and disease-free survival (DFS) and overall survival (OS) in patients with BLCA. The HR values (1.6 and 1.2, respectively) indicated a trend toward worse disease-free and overall survival in patients with high PAK4 expression, although the differences did not reach statistical significance (Fig 1c, d). These observations suggest a potential involvement of PAK4 in BLCA pathogenesis. To explore the clinical significance of the PAK4 protein in patients with BLCA, we evaluated PAK4 protein levels in a human tissue microarray (TMA), which included both benign and tumor tissues. Our analysis revealed significantly higher PAK4 protein levels in tumor tissues than in benign tissues (Fig 1e, f; *p* < 0.001). These findings open avenues for future research to determine the functional role of PAK4 in the development and progression of BLCA, as well as its possible utility as a therapeutic target.

### Inhibition of PAK4 induced apoptosis and disrupted the cell cycle in BLCA cells

**Fig 2:**
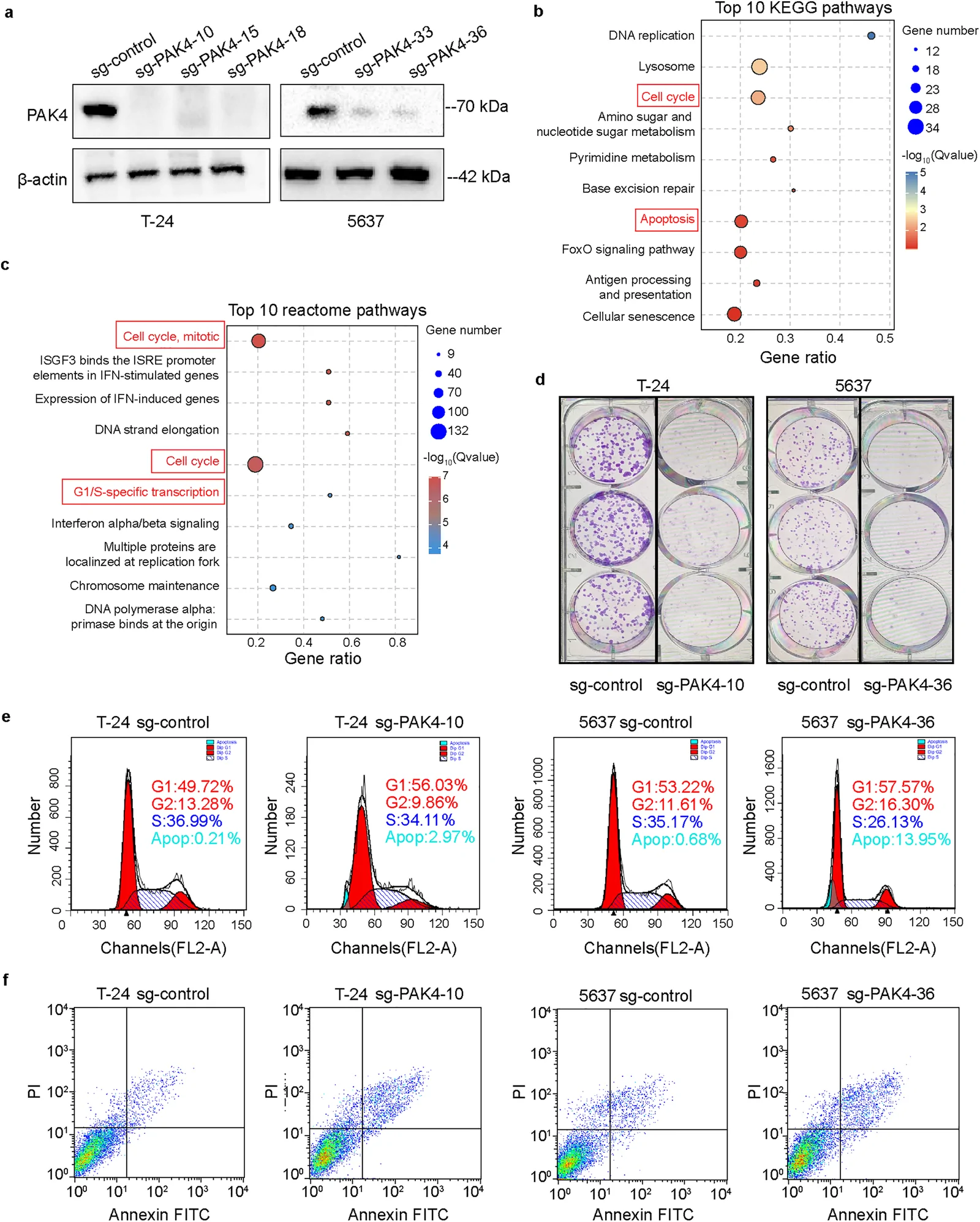
Knockout of PAK4 inhibited cell growth and apoptosis in BLCA. (a) Construction and validation of stable PAK4 knockout BLCA cell lines. (b) The top 10 KEGG pathways were analyzed from the RNA-sequencing results of the T-24 cell line with PAK4 knockout compared with the control group. The size of the bar plots represents the number of DEGs annotated in each KEGG pathway, and the color gradient of the bar plots represents significance. (c) Results from RNA sequence analysis of the RNA sequence results of the T-24 cell line with PAK4 knockout compared with the control group for Reactome. The size of the bar represents the number of enriched DEGs in the pathway, and the color gradient of the bar represents significance (the value of -log10 (Q value)). (d) Colony formation images of the sg-control and sg-PAK4 subclones in T-24 and 5637 cells, respectively. The cells were seeded in 6-well dishes, cultured for 7 days and stained with crystal violet. (e) Representative images of the cell cycle for the control and PAK4 knockdown subclones in T-24 and 5637 cells, respectively. The cells were seeded in 6-well dishes, cultured for 2 days, stained with PI and detected via a flow cytometer. (f) Representative images of the apoptosis of control and PAK4 knockdown subclones in T-24 and 5637 cells, respectively. The cells were seeded in a 6-well dish and cultured for 2 days. The samples were then stained with a cell apoptosis kit and detected with a flow cytometer.

To investigate the biological role of PAK4 in the progression of BLCA, we generated a monoclonal cell line that is deficient in PAK4 and conducted sequencing analysis. As shown in Fig 2a, we successfully developed PAK4-deficient monoclonal cell lines derived from both the T-24 and the 5637 cell lines. Specifically, the sg-PAK4–10 clone from the T-24 cell line and the sg-PAK4–36 clone from the 5637 cell line were meticulously selected for further investigative studies. We performed pathway enrichment analysis on the RNA sequencing data from the T-24 control and T-24 sg-PAK4–10 groups. Following the knockdown of PAK4, we identified 1506 upregulated genes and 647 downregulated genes, with an FDR of ≤0.05 and a fold change in expression of ≥2 (S1 Fig in S2 File). KEGG pathway enrichment analysis indicated that the knockdown of PAK4 impacts apoptosis and the cell cycle in BLCA tumor cells, as illustrated in Fig 2b. Similarly, the results of the Reactome enrichment analysis suggested that the knockdown of PAK4 affects the transition from the G1 to S phase of the cell cycle in BLCA tumor cells (Fig 2c). To validate the results of the KEGG pathway enrichment analysis, we designed experiments aimed at analyzing the progression of the cell cycle and apoptosis. As shown in Fig 2d, PAK4 knockout inhibited colony growth in T-24 and 5637 cells. As shown in Fig 2e, compared with that of the T-24 control cells, the proportion of the T-24/sg-PAK4–10 cells in the G1 phase of the cell cycle increased from 49.72% to 56.03%. Similarly, the proportion of the 5637/sg-PAK4–36 cells in the G1 phase increased from 53.22% to 57.57% compared with that of the control 5637 cells. Cell cycle fitting identified a small proportion of apoptotic cells, 2.97% of T-24/sg-PAK4–10 cells and 13.95% of 5637 sg-PAK4–36 cells. We then used a more sensitive cell apoptosis detection kit that can simultaneously detect early apoptosis and necrosis to detect the state of cell apoptosis. As shown in Fig 2f, we observed alterations in apoptosis among the PAK4-knockout cells. The apoptosis rates of T-24/sg-PAK4–10 cells increased from 9.8% to 20.0% upon PAK4 depletion. In the 5637 cell line, a comparable increase was noted, with apoptosis rates increasing from 11.6% to 18.65%. These findings indicate a correlation between PAK4 suppression and the induction of apoptosis in BLCA cells. This shift in the cell cycle distribution and cell apoptosis underscores the role of PAK4 in cell growth and death mechanisms.

### Inhibition of PAK4 induced apoptosis and disrupted the cell cycle in BLCA cells

**Fig 3:**
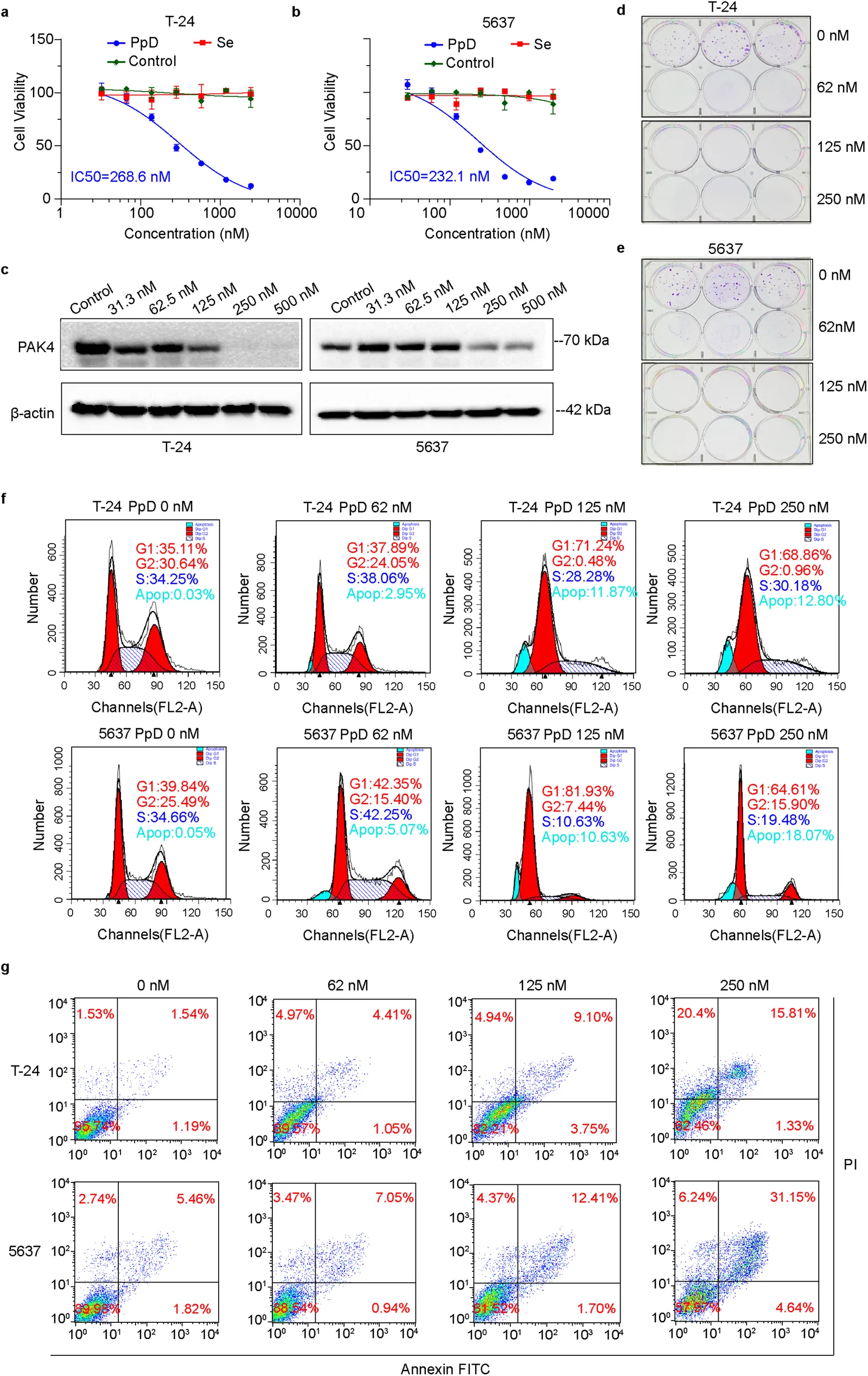
PpD selectively degrated PAK4 and its inhibits tumor cell growth in BLCA. **(a, b)** Cell viability assays were performed on BLCA cells treated with specified concentrations of PpD, the Se-mutation peptide (labeled ‘Se’), and the free PAK4 PROTAC peptide serving as a control. The treatments were applied for a period of 48 hours. The IC50 values for PpD were determined to be 268.6 nM for T-24 cells and 232.1 nM for 5637 cells. **(c)** The ability of PpD to selectively degrade PAK4 was evaluated via western blotting. This evaluation was conducted on T-24 and 5637 cells after exposure to various concentrations of PpD for 48 hours. **(d, e)** Colony formation analysis results for BLCA cell lines. T-24 and 5637 cells were treated with 0, 62, 125, or 250 nM PpD for 7 days, as determined via crystal violet staining. **(f)** Representative images of the cell cycle after 0, 62, 125, and 250 nM PpD treatment in T-24 and 5637 cells. The cells were seeded in 6-well dishes, treated with PpD for 2 days, stained with PI and detected via a flow cytometer. **(g)** Representative images of the apoptosis of 0, 62, 125, and 250 nM PpD-treated T-24 and 5637 cells. The cells were seeded in a 6-well dish and treated with PpD for 2 days. The samples were then stained with a cell apoptosis kit and detected with a flow cytometer.

We engineered a specialized peptide proteolysis targeting chimera (PROTAC) drug with selectivity for PAK4 degradation in kidney carcinoma cells (termed PpD) [27]. PpD significantly suppresses tumor growth in immunocompetent mouse models. Tumor inhibitory effects were also observed in minipatient-derived xenograft and patient-derived organoid models of kidney carcinoma [27]. Considering the high expression of PAK4 in BLCA, we have extended the application of PpD to BLCA. PpD can also suppress cell growth and degrade PAK4 in BLCA cells. To evaluate the efficacy of PpD in inhibiting cancer cell proliferation, we utilized a free PAK4 PROTAC peptide (denoted as the control) and a nanoselenium-mutated peptide (referred to as Se) as negative controls [27]. As illustrated in Fig 3a, b, our findings revealed that PpD is notably potent in inhibiting cell growth across the tested cell lines, achieving IC_50_ values of 268.6 nM in T-24 cells and 232.1 nM in 5637 cells. The potential of various concentrations of PpD to degrade PAK4 was evaluated in BLCA cell lines following a 24-hour treatment period. In the T-24 and 5637 cell lines, PpD significantly decreased the PAK4 protein levels (Fig 3c). We selected 62.5, 125, and 250 nM concentrations of PpD for use in our subsequent experiments. The clonogenic potential of BLCA cells was assessed via a colony formation assay. As shown in Fig 3d, e, PpD significantly inhibited colony growth, even at 62.5 nM, in both T-24 and 5637 cells. Furthermore, we conducted experiments to determine whether the effects of PpD treatment replicate those observed upon PAK4 knockout in the T-24 and 5637 BLCA cell lines (Fig 2e, f). To achieve this goal, we employed flow cytometry to assess the impact of PpD on the cell cycle in BLCA cells. As shown in Fig 3f, PpD increased the proportion of BLCA cells in the G1 phase of the cell cycle. Meanwhile, we found a small fraction of cells undergoing apoptosis in cell cycle assay. In further to test the PpD’s effect on BLCA cells popotosis, we designed cell apopotosis assay, under PpD drug treatment at 250 nM, T-24 cells presented a significant increase in the proportion of apoptosis cells, from 4.26% to 37.53%. Similar phenomena of apoptosis (from 10.0% to 42.08%) were observed in 5637 cells treated with PpD (Fig 3f). T24 and 5637 cell apoptosis significantly increased in a PpD concentration-dependent manner (Fig 3g). This shift in the cell cycle distribution and cell apoptosis underscores the effects of PpD on cell growth and death mechanisms.

### Mechanisms of PpD in BLCA: ERK1/2-Cyclin D1-CDK4 and apoptosis regulation

**Fig 4:**
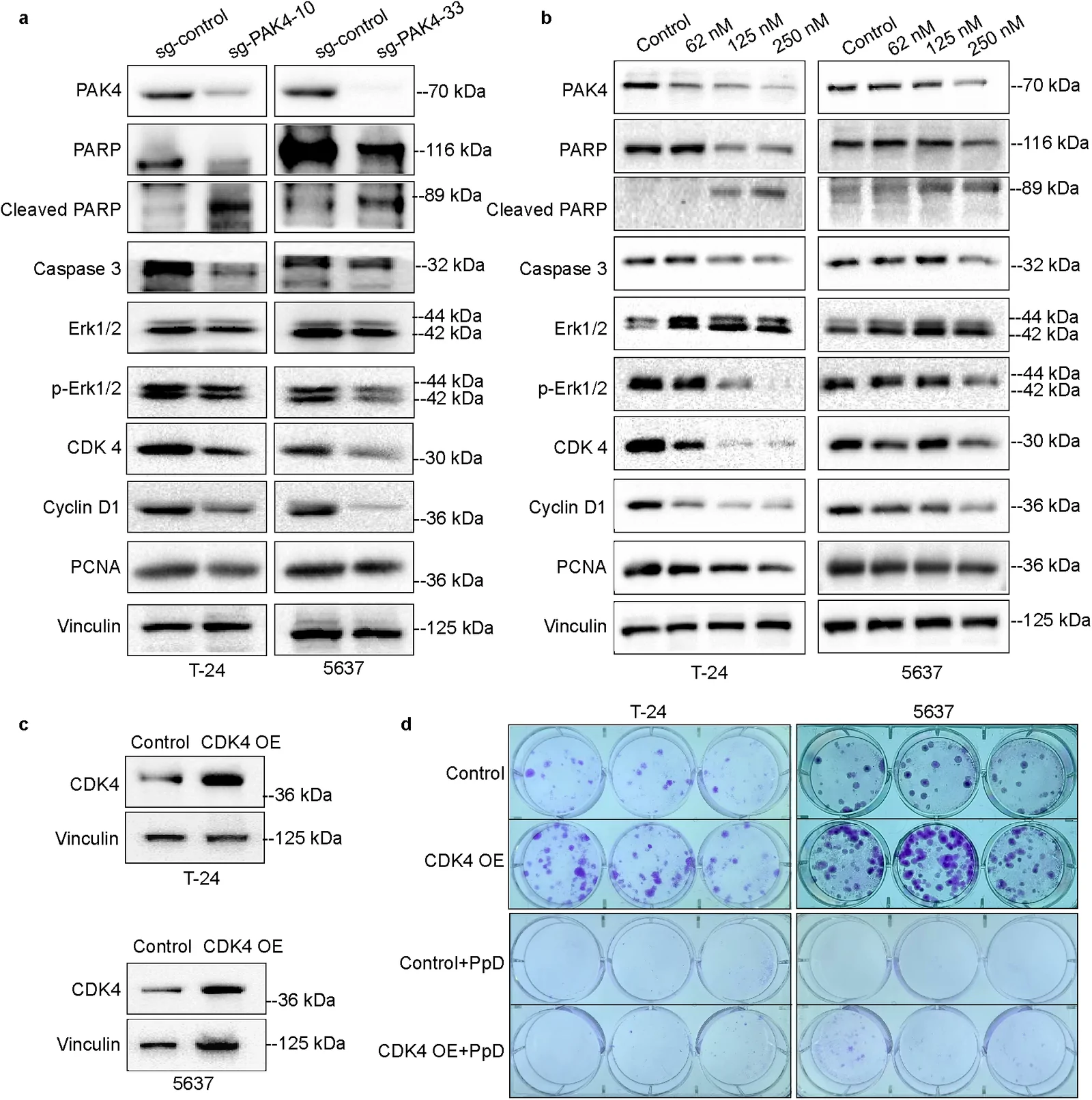
PpD downregulated the ERK1/2-CDK4 pathway and induced caspase-3-dependent apoptosis in BLCA cells. (a) Cell lysates from T-24 cells, 5637 cells, and the sg-PAK4 subclone were analyzed by immunoblotting. The expression levels of cleaved PARP, PAK4, PARP, Caspase 3, Cyclin D1, CDK 4, Erk1/2, PCNA, and Vinculin were assessed. (b) T-24 and 5637 cells were treated with 0, 62, 125, or 250 nM PpD for 48 h, after which the cell lysates were subjected to SDS–PAGE followed by immunoblotting. cleaved PARP, PAK4, PARP, Caspase 3, Cyclin D1, CDK 4, Erk1/2, PCNA, and Vinculin. (c) Cell lysates from T-24 cells, 5637 cells, and the CDK4 overexpression cells were analyzed by WB. The expression levels of cleaved CDK 4 and Vinculin were assessed. (d) Colony formation images of T-24 and 5637 cells overexpressing CDK4 or treated with PpD. Cells were seeded in 6-well dishes, cultured for 7 days, and stained with crystal violet, respectively. The cells were seeded in 6-well dishes, cultured for 7 days and stained with crystal violet.

To explore the growth- and apoptosis-inhibiting mechanisms of PAK4 and PpD in BLCA cells, the expression levels of the cyclin D1 and CDK4 proteins, which are crucial for cell cycle progression, were measured. As shown in Fig 4a, b, significantly decreased cyclin D1 levels were observed in T-24 and 5637 PAK4 knockout cells. Concurrently, decreased expression of cyclin D1 was also observed in PpD-treated cells (Fig 4b). Additionally, the expression of PCNA, a marker of cell growth, was substantially reduced by PpD treatment and PAK4 knockout. The ERK1/2 pathway regulates cyclin D1-CDK4 expression. Inhibition of this pathway decreases cyclin D1-CDK4 and impedes cell cycle progression. To investigate whether PpD and PAK4 influence cyclin D1-CDK4 by modulating the ERK1/2 pathway, T-24 and 5637 cells were exposed to PpD. The subsequent reductions in phosphorylated ERK1/2 indicate that PpD potentially diminishes cyclin D1/CDK4 expression and induces G1 arrest in renal cancer cells. An analogous phenomenon was observed in the cell line after the knockout of PAK4 (Fig 4a, b).

To dissect the molecular underpinnings of the apoptosis induced by PpD treatment and PAK4 knockout in BLCA cells, caspase 3, PARP and cleaved PARP, which are integral players in the apoptotic cascade, were detected. Compared with those in the control cells, the caspase-3 levels in the T-24 and 5637 cells subjected to PpD treatment were notably lower (Fig 4b). A similar decreasing trend was observed for caspase-3 and PARP, where the level of cleaved PARP protein was significantly greater following PpD treatment than in the controls (Fig 4b). Meanwhile, we also confirmed that caspase-3 and PARP protein are all decreased, and cleaved PARP protein is increased in the T-24 and 5637 cell lines in which PAK4 was knocked out (Fig 4a). To further verify whether cyclin D1-CDK4 affects the effect of PpD on cell apoptosis, we overexpressed CDK4 in T-24 and 5637 cells (Fig 4c). Colony formation assay showed that CDK4-overexpressing cells proliferated significantly faster than control cells both in T-24 and 5637 cells (Fig 4d). Importantly, Ppd treatment inhibited cell proliferation in both control and CDK4-overexpressing cells.

### PpD inhibited BLCA tumor cell growth in nude mice

**Fig 5:**
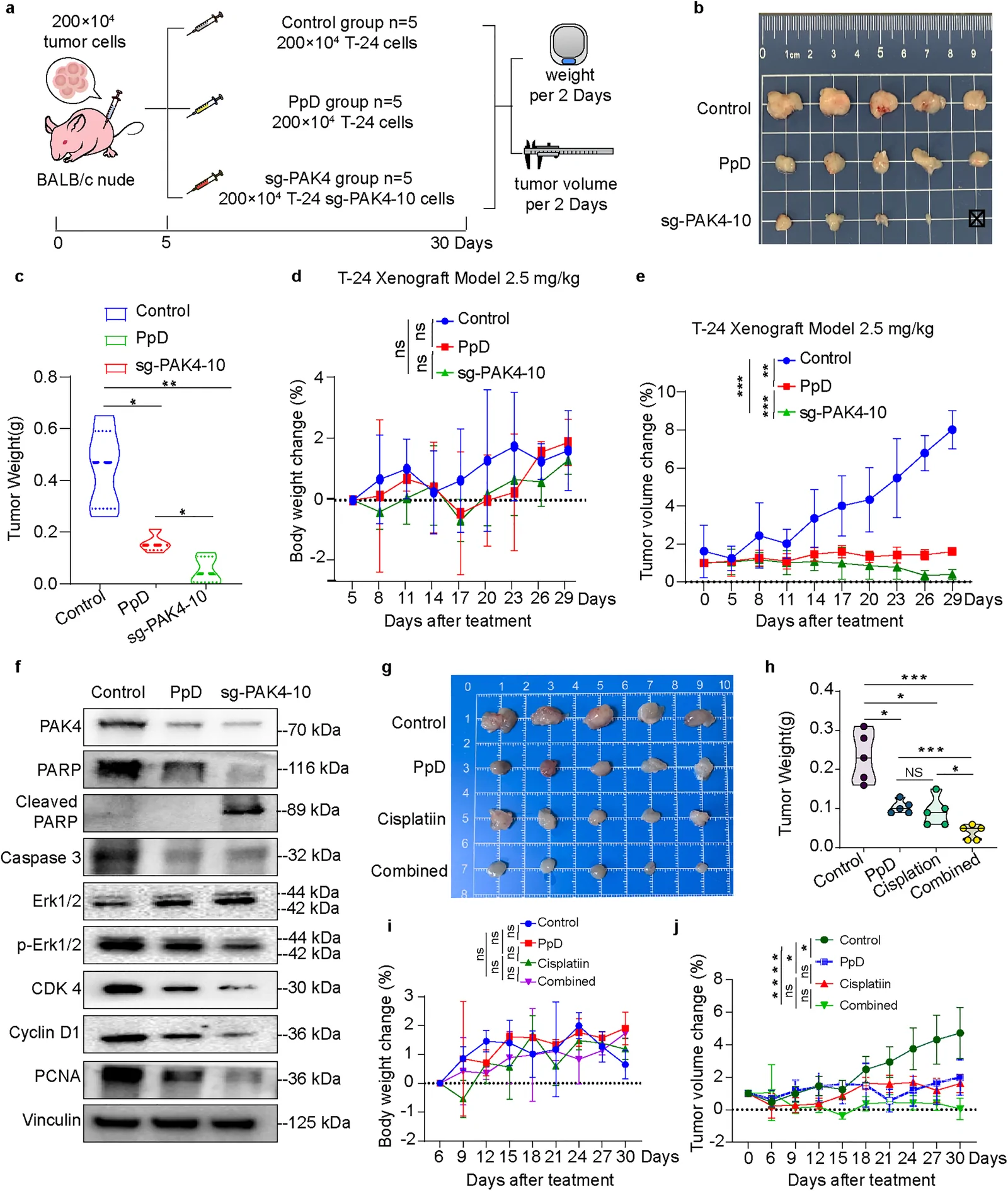
Pharmacological degradation of PAK4 effectively inhibited the growth of BLCA *in vivo.* (a) Schematic representation of tumor-bearing BALB/c mice treated with the indicated drugs. Ten BALB/c mice were subcutaneously injected with 200 × 10^4^ T-24 cells and randomly separated into 2 groups (the control group and the PpD treatment group). Five BALB/c mice were subcutaneously injected with 200 × 10^4^ T-24/sg-PAK4-10 cells. The control and T-24/sg-PAK4-10 group mice were treated with PBS every 3 days, and the PpD treatment group mice were treated with 2.5 mg/kg PpD every 3 days. The weights and tumor volumes of the BALB/c nude mice were recorded every 3 days. (b) Photograph of tumors excised at the end of the tumor-bearing BALB/c nude mouse experiment in the T-24 control group, PpD treatment group (with 2.5 mg/kg PpD treatment), and T-24/sg-PAK4-10 group. In the T-24/sg-PAK4-10 group, one mouse did not exhibit any tumors from the beginning to the end of the experiment, with an “×” symbol on the photo. (c) Tumors were excised from each group of mice at the end of the experiment and weighed. The data are presented as the means ± SDs. Statistical analysis of the data among the groups was performed via one-way ANOVA; * represents p < 0.05; ** represents p < 0.01. (d) Changes in the body weights of the animals were detected throughout the entire experiment. The mice were treated with the indicated amount of PBS or PpD. Statistical analysis of the data among the groups was performed via one-way ANOVA. Error bars indicate ± SDs; ns represents no statistical significance. (e) Tumor volume changes in the xenografted mice throughout the 30‑day experiment. Statistical analysis among the groups was performed using one‑way ANOVA. Error bars represent ± SD (n = 5). **p < 0.01, ***p < 0.001. (f) WB showed the expression levels of cleaved PARP, PAK4, PARP, Caspase 3, Cyclin D1, CDK 4, Erk1/2, PCNA, and Vinculin. Tissue lysates from the control, PpD-treated, and T-24 sg-PAK4-10 groups, respectively. (g) Representative photographs of excised tumors from BALB/c nude mice bearing tumors at the end of the experiment. in the T-24 control group, PpD treatment group (with 2.5 mg/kg PpD treatment), Cisplatilin treatment group (with 2.5 mg/kg PpD treatment), and PpD + Cisplatin combination group (alternating injection: PpD first, then Cisplatin). (h) At the end of the experiment, tumors from all groups were dissected and weighed. Values are shown as mean ± SD. One-way ANOVA was applied for statistical comparisons. *p < 0.05, ***p < 0.001. (i) Mouse body weight was measured throughout the experimental period. One-way ANOVA was used for statistical comparison between groups. Error bars show standard deviation (SD); ns denotes no statistical significance. (j) Tumor volume changes of xenografted mice during the 30-day experiment. Group differences were analyzed via one-way ANOVA. Error bars indicate ± SD n=5. *p < 0.05, ****p < 0.001.

**Fig 6:**
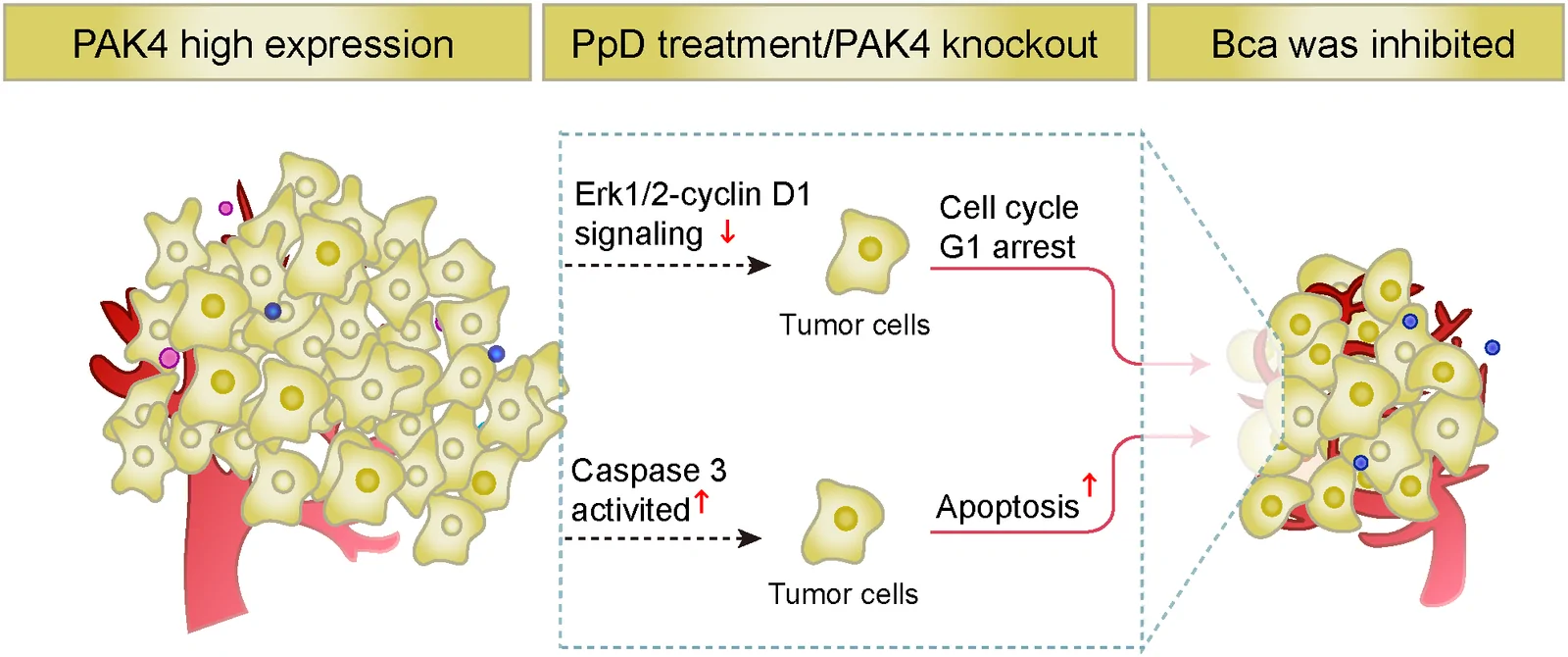
Schematic diagram of PpD for BLCA therapy. PpD, a highly potent and selective PAK4 degrader, targets and degrades the PAK4 protein in BLCA cells. Treatment with the PpD drug inhibits the ERK1/2-CDK4 signaling pathway, leading to an impediment in the cell cycle transition from the G1 phase to the S phase. Furthermore, it induces tumor cell apoptosis, thereby suppressing the proliferation and growth of BLCA tumor cells.

The inhibitory effect of PpD on tumor growth was assessed using a T-24 xenograft model. Nude mice were inoculated subcutaneously with either 2 × 10^6^ T-24 (Fig 5a, n = 5) or T-24/sg-PAK4–10 cells (Fig 5a, n = 5). A control group (2 × 10^6^ T-24, n = 5) received PBS injections, whereas the T-24 sg-PAK4–10 group (n = 5) served as a positive control, reflecting the inhibitory effect of PAK4 on BLCA progression. Based on our previous results, increasing the PpD dose to 5.0 mg/kg failed to enhance the antitumor effect in the renal cancer subcutaneous tumor model, indicating that 2.5 mg/kg represents the optimal dosage for maximal therapeutic benefit. Therefore, we selected the indicated dose of 2.5 mg/kg for the PpD group (2 × 10⁶ T-24 cells, n = 5). The efficacy of tumor growth suppression was evaluated based on both tumor volume and weight measurements. As depicted in Fig 5b, the PpD treated group exhibited a markedly reduced tumor volume in comparison to the control group. Similarly, tumor weights were significantly diminished in the PpD group when compared to the control group (Fig 5c, *p* < 0.05). However, the *in vivo* anti-proliferative impact of PpD on BLCA cells did not fully align with the effects observed in cell lines with a *PAK4* gene knockout (p < 0.05). Body weight and general health status of the mice were monitored at three-day intervals throughout the study. There was no significant difference in body weight loss between the PpD-treated group and the control group, as illustrated in Fig 5d. In Fig 5e, over the 30-day period the control group showed a steady increase (≈8% at day 30). The PpD treated group maintained tumor size near baseline (p < 0.05), while the sg-PAK4–10 group exhibited markedly suppressed tumor growth (p < 0.001), keeping the change at ~0% and even showing a slight regression after day 12. In Fig 5f, tumor tissues were collected from the xenograft experiment. WB analysis of tissue lysates from the control, PpD-treated, and T-24 sg-PAK4–10 groups showed the expression levels of cleaved PARP, PAK4, PARP, cleaved Caspase-3, Cyclin D1, CDK4, p-ERK1/2, total ERK1/2, PCNA, and Vinculin. Compared to the control group, both PpD treatment and PAK4 knockout reduced PAK4 protein levels, decreased ERK1/2 phosphorylation, and downregulated CDK4 expression. To compare the anti-tumor efficacy of PpD with cisplatin, a first-line clinical agent for BLCA, we evaluated monotherapies and combination therapy in a xenograft model. As illustrated in Fig 5g, treatment with PpD, or cisplatin led to a substantial decrease in tumor volume relative to the control. As showed in Fig 5h, tumor weights were significantly reduced in all treatment groups compared to control group (PpD, *p* < 0.05; cisplatin, *p* < 0.05; combined, *p* < 0.001), with the combination group showing significantly lower tumor weights than cisplatin monotherapy (*p* < 0.05), while no significant difference was observed between the two monotherapies. All mice were well-tolerated, with no significant body weight loss in any group throughout the study (Fig 5i). Consistent with the Fig 5h results, the control group exhibited progressive tumor growth over 30 days (Fig 5j), whereas all treatments markedly suppressed tumor volume expansion, with the combination therapy exerting the most potent inhibitory effect (*p* < 0.05, *p* < 0.001 compared with control). Collectively, our findings indicate that PpD effectively suppresses the in vivo growth of BLCA cells (Fig 6).

## Discussion

We engineered PpD to concurrently fulfill three distinct design criteria: 1) it circumvents the ATP-binding site of protein kinases, which exhibit high structural conservation in three dimensions and could compromise target specificity; 2) mutations within the PAK4 kinase domain do not impair target recognition; and 3) it addresses the challenge of PAK4 kinase activity being triggered by alternative domains [27]. Preclinical and clinical investigations of renal cell carcinoma have substantiated its marked suppression of tumor growth [24,27–29]. When coupled with other therapeutics, including those targeting the HIF/VEGF pathway and immune checkpoint inhibitors, PpD shows potential for the treatment of metastatic kidney cancer. Experts from the Memorial Sloan Kettering Cancer Center have lauded PpD in a dedicated review published in the journal *eBioMedicine*, highlighting its prospective clinical value in future combinatorial oncology treatments [30].

From the perspective of mechanistic rationale, inhibition of PAK4 activity may constrain the growth and progression of PAK4-high tumors. Combining the use of PAK4 inhibitors with targeted drugs or immune checkpoint inhibitors improved therapeutic outcomes [31–33]. In the present study, we verified that PAK4 is highly expressed in BLCA tissues and tends to correlate with unfavorable clinical outcomes. However, such immunotherapeutic synergies cannot be extrapolated to the current BLCA study due to the limitation of the animal model used. Analysis via the GEPIA tool revealed that the expression of PAK4 in tumor tissues was greater than that in normal tissues. Of note, high PAK4 expression showed only a trend toward association with increased disease progression risk in BLCA patients; notably, the Kaplan–Meier survival analysis failed to reach the conventional statistical significance threshold (p < 0.05) (Fig 1).

CDK4/6 inhibitors have been approved for clinical cancer treatment. The results of a Phase III clinical trial published in the New England Journal of Medicine revealed that the combination of CDK4/6 inhibitors with antiestrogen therapy can significantly improve the progression-free survival of patients with advanced breast cancer who are hormone receptor positive and human epidermal growth factor receptor 2 negative (27.6 months vs. 14.5 months), reducing the risk of progression by approximately 44% [34–36]. CDK4/6 inhibitors can also benefit patients with advanced small cell lung cancer [37]. In clinical studies of BLCA, CDK4/6 inhibitors have been confirmed to effectively kill BLCA cells [38–40]. Our RNA-seq results revealed that PAK4 regulates the cell cycle and apoptosis in BLCA cells (Fig 3). Knockout and PpD drug treatment confirmed that clearing the content of PAK4 in BLCA cells can induce apoptosis and cell cycle arrest (Fig 3-4). Our WB experiments confirmed that PpD inhibited the Erk1/2 pathway, reducing the expression of CDK4 and causing cell cycle arrest (Fig 4). The ablation or inhibition of PAK4 enhances the infiltration of tumor-killing cells. Consequently, compared with CDK4/6 inhibitors, PpD is expected to exhibit potential superior anti-tumor activity in the clinical extermination of BLCA cells. Based on RNA-seq enrichment analysis, we focused our mechanistic investigation on the ERK1/2/CDK4 pathway. Considering that PAK4 is a versatile signaling hub that modulates multiple biological cascades including STAT3, PI3K/AKT and Wnt/β-catenin, we further explored the regulatory effect of PpD on these pathways. Our previous findings results confirmed that PpD markedly inhibits the activation β-catenin [27]. This evidence demonstrates that the biological phenotypes induced by PpD are not merely localized alterations in cell cycle progression, but arise from the combined regulation of multiple PAK4 downstream signaling pathways. Future studies will systematically investigate the crosstalk between these signaling axes and their collective functions in PpD-mediated biological effects.

Research has reported that PAK4 knockout and the PAK4 inhibitor KPT-9274 both have excellent antitumor effects when combined with immune checkpoint inhibitors in *in vitro* and *in vivo* experiments. Notably, the complete remission rate of the PAK4 knockout combined with PD-1 treatment group was 42% (3 out of 7 mice experienced complete tumor disappearance), whereas KPT-9274 combined with PD-1 treatment inhibited only tumor growth. In our animal model study, both the T-24 sg-PAK4 group and the PpD drug treatment group effectively inhibited tumor cell growth (Fig 5). This finding was consistent with research reported by Trott, J.F., *et al*. [24]. However, given that our *in vivo* experiments were performed in BALB/c nude mice with deficient T-cell-mediated immune function, we cannot draw definitive conclusions regarding the regulatory effect of PAK4 ablation or inhibition on the infiltration of tumoricidal immune cells. Validation in immunocompetent syngeneic models is required in future work.

Here, we should mentioned that PAK4 inhibitors rely on binding to the ATP-binding site or specific conformations to block enzymatic activity; however, this approach merely suppresses PAK4 protein function without eliminating its physical presence [24,41,42]. In contrast, PpD can theoretically “eradicate” PAK4 from cells, thereby abrogating all pathogenic functions, including both enzymatic and non-enzymatic activities. The “clearance” mechanism of PROTACs enables the elimination of low-abundance or “undruggable” proteins (such as mutants or specific domains), which is typically challenging to achieve with conventional inhibitors [43,44]. Meanwhile, PpD exhibited a gradual time-dependent degradation profile in serum, the calculated serum half-life of PpD was 10.97 h, indicating that the nano-selenium delivery system substantially prolonged the apparent serum stability of PpD and protected it from immediate degradation in the serum environment (S2 Fig in S2 File). However, although PROTACs exhibit high degradation efficiency *in vivo*, achieving an appropriate pharmacokinetic balance remains a substantial challenge. The crux lies in reconciling the potent degradation capacity of PROTACs with favorable pharmacokinetic properties, while ensuring precise activity in specific tissues [45].

In conclusion, our study validates the anti-tumor role and therapeutic potential of PpD in BLCA, expands its applicable research scope, and provides a novel preclinical reference for combination treatment of BLCA. The present findings offer meaningful mechanistic insights and preclinical translational implications for the management of solid tumors.

## Supporting information

S1 File Raw images. (PDF)

S2 File Volcano plot displaying differentially expressed genes between T-24 control cells and PAK4-knockout T-24/sg-PAK4–10 cells. (a) The identified differentially expressed genes were showed by volcano plot between control group and sg-PAK4–10 group. 1506 up-expression genes and 647 down-expression genes in treatment group compared with control group. FDR ≤ 0.05 and expression fold-change ≥ 2 were set as the cut-off criterion of significant difference. Red dots, significantly upregulated genes Yellow dots, significantly downregulated genes. Blue dots, nondifferentially expressed genes. Serum degradation curve of PpD with calculated elimination half-life. (a) Time-dependent degradation profile of PpD in serum. The elimination half-life of PpD was calculated to be 10.97 h based on the residual drug percentage decay curve.
(DOCX)
