# Supplementary material for: PAK4-targeting PROTAC inhibits bladder cancer cell proliferation
Source: PLoS One. 2026 Sep 21;21(9):e0358561. doi: 10.1371/journal.pone.0358561 (PMC13592572; doi:10.1371/journal.pone.0358561)

PAK4-Targeting PROTAC Inhibits Bladder Cancer Cell Proliferation

Raw unadjusted, uncropped blot for Figure 2a

Protein: PAK4

Samples loaded order (left to right): marker, T-24 sg-control,T-24 sg-PAK4-10, T-24 sg-PAK4-15, T-24 sg-PAK4-18, marker

Lanes removed in final figure: Lane 1,6 (marked ×)

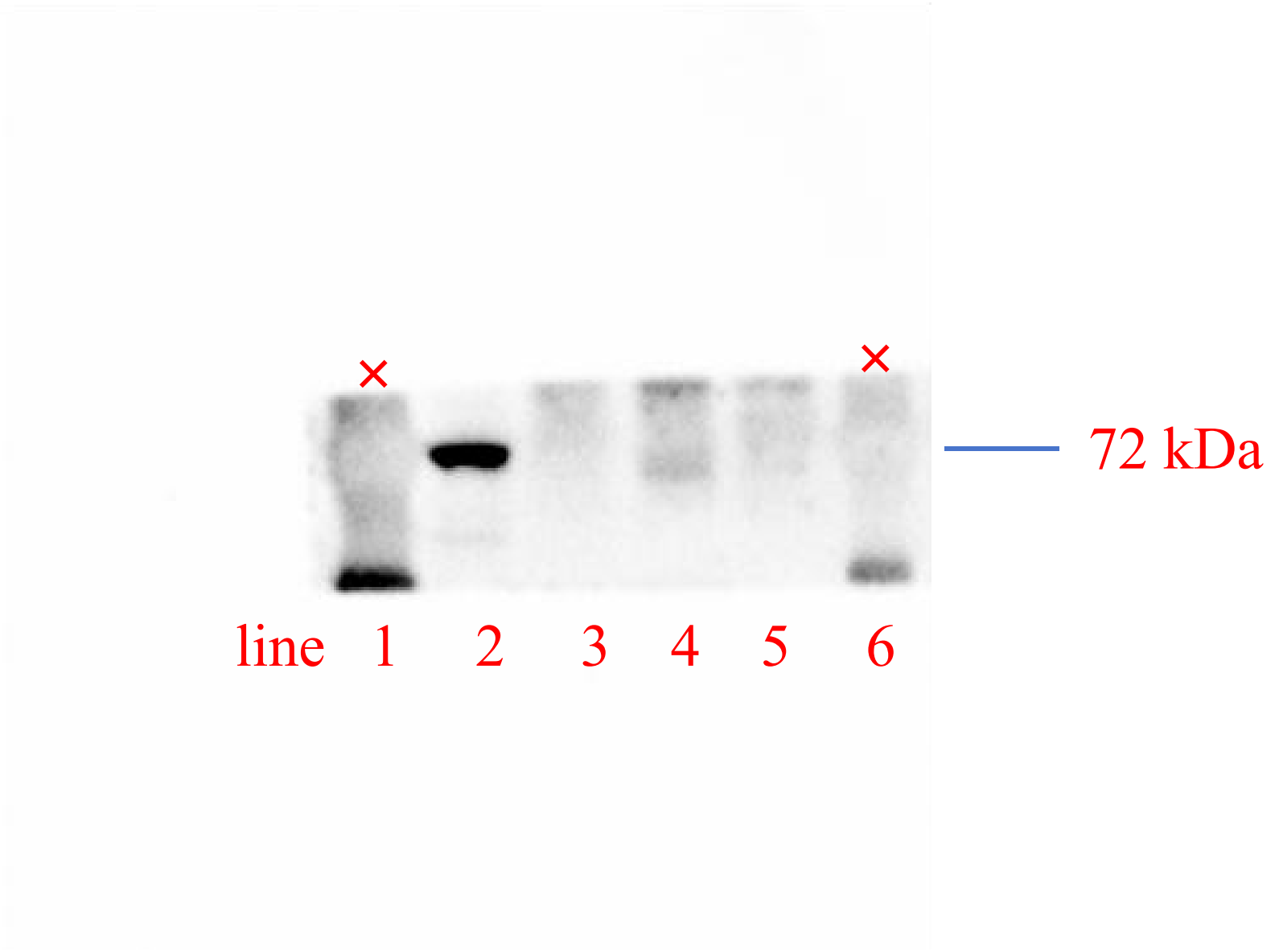

Raw unadjusted, uncropped blot for Figure 2a

Protein: β-actin

Samples loaded order (left to right): T-24 sg-control,T-24 sg-PAK4-10, T-24 sg-PAK4-15, T-24 sg-PAK4-18

Lanes removed in final figure: none

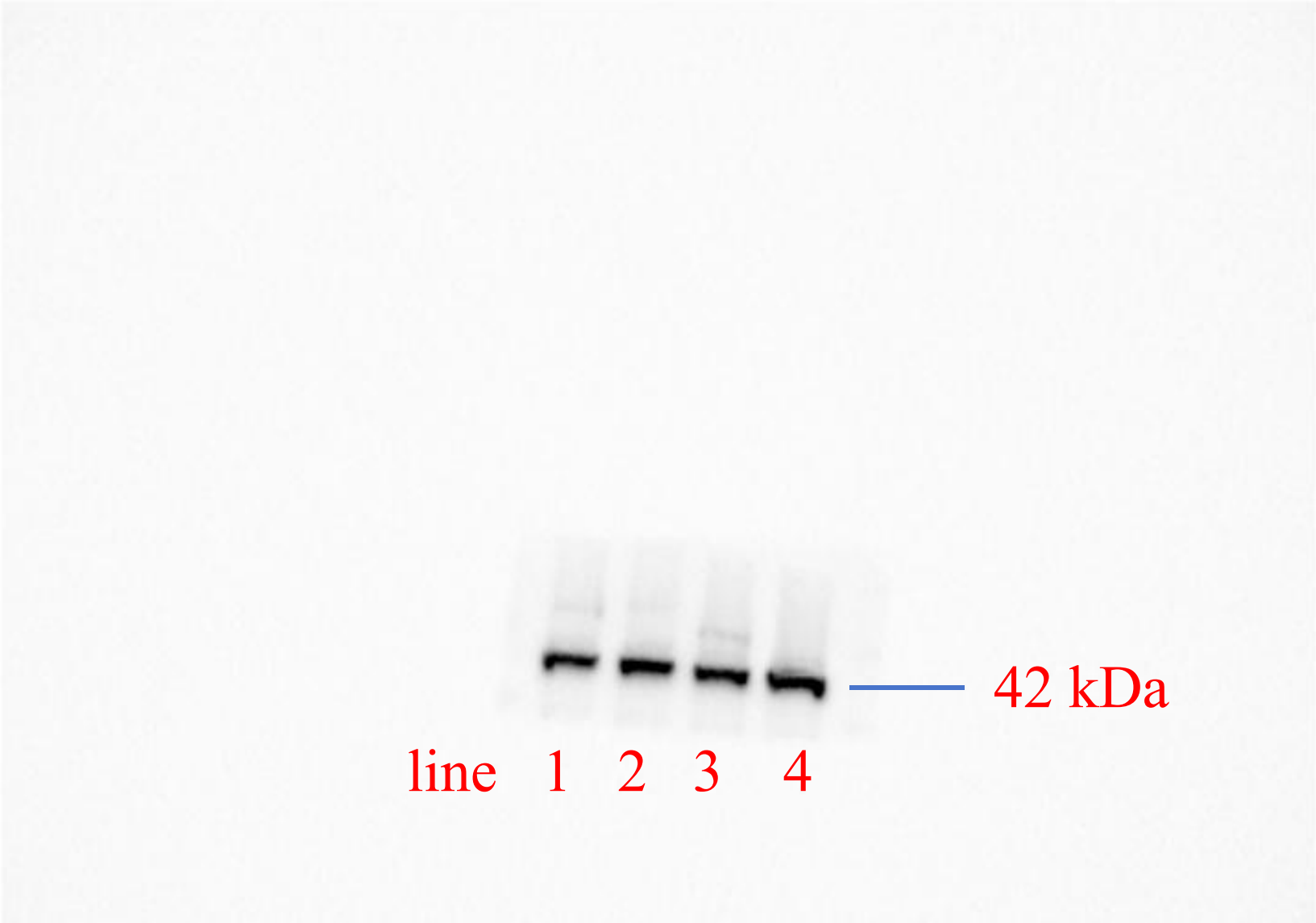

Raw unadjusted, uncropped blot for Figure 2a

Protein: PAK4

Samples loaded order (left to right): 5637 sg-control, 5637 sg-PAK4-33, 5637 sg-PAK4-33

Lanes removed in final figure: none

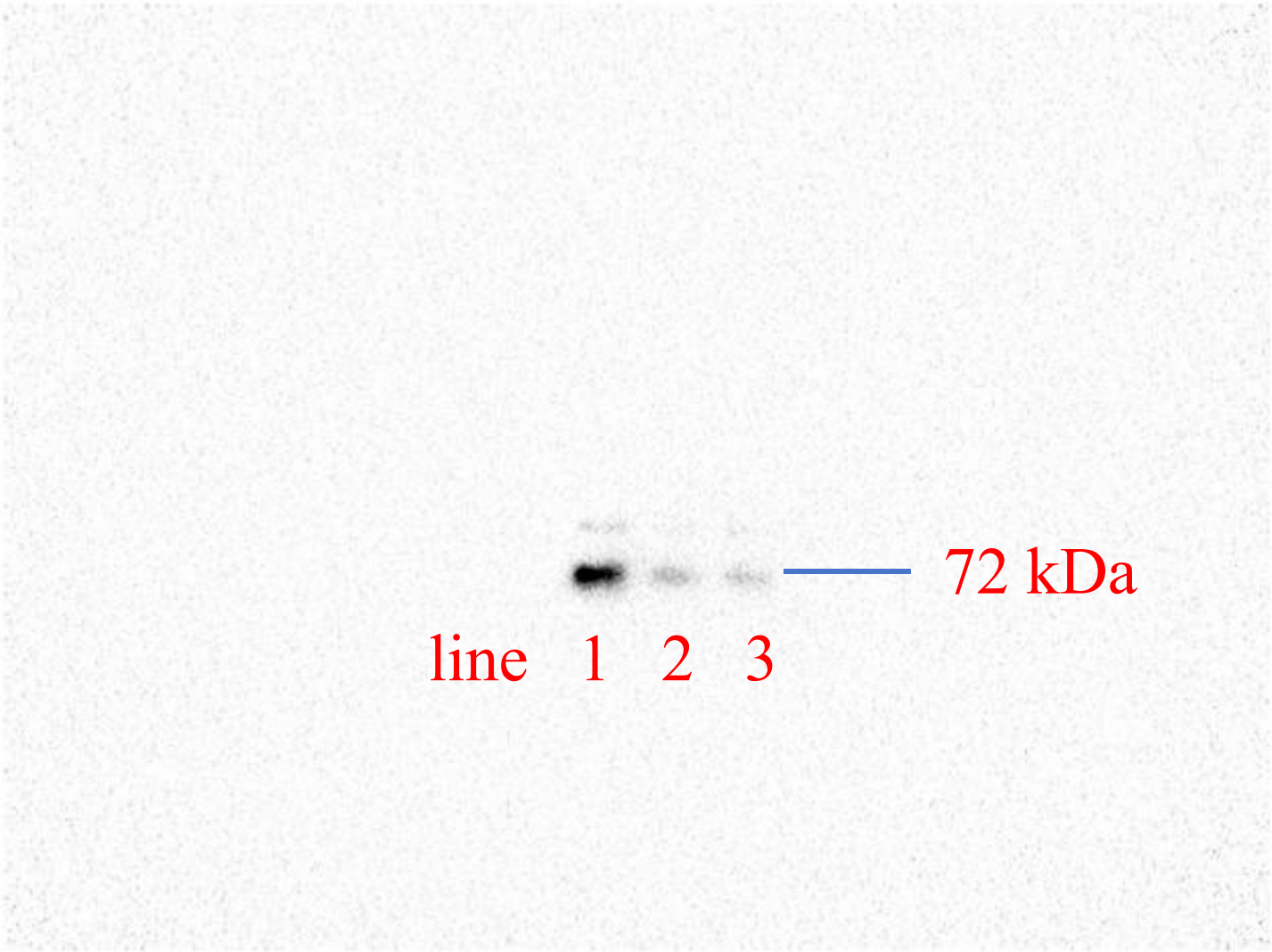

Raw unadjusted, uncropped blot for Figure 2a

Protein: β-actin

Samples loaded order (left to right): 5637 sg-control, 5637 sg-PAK4-33, 5637 sg-PAK4-33

Lanes removed in final figure: none

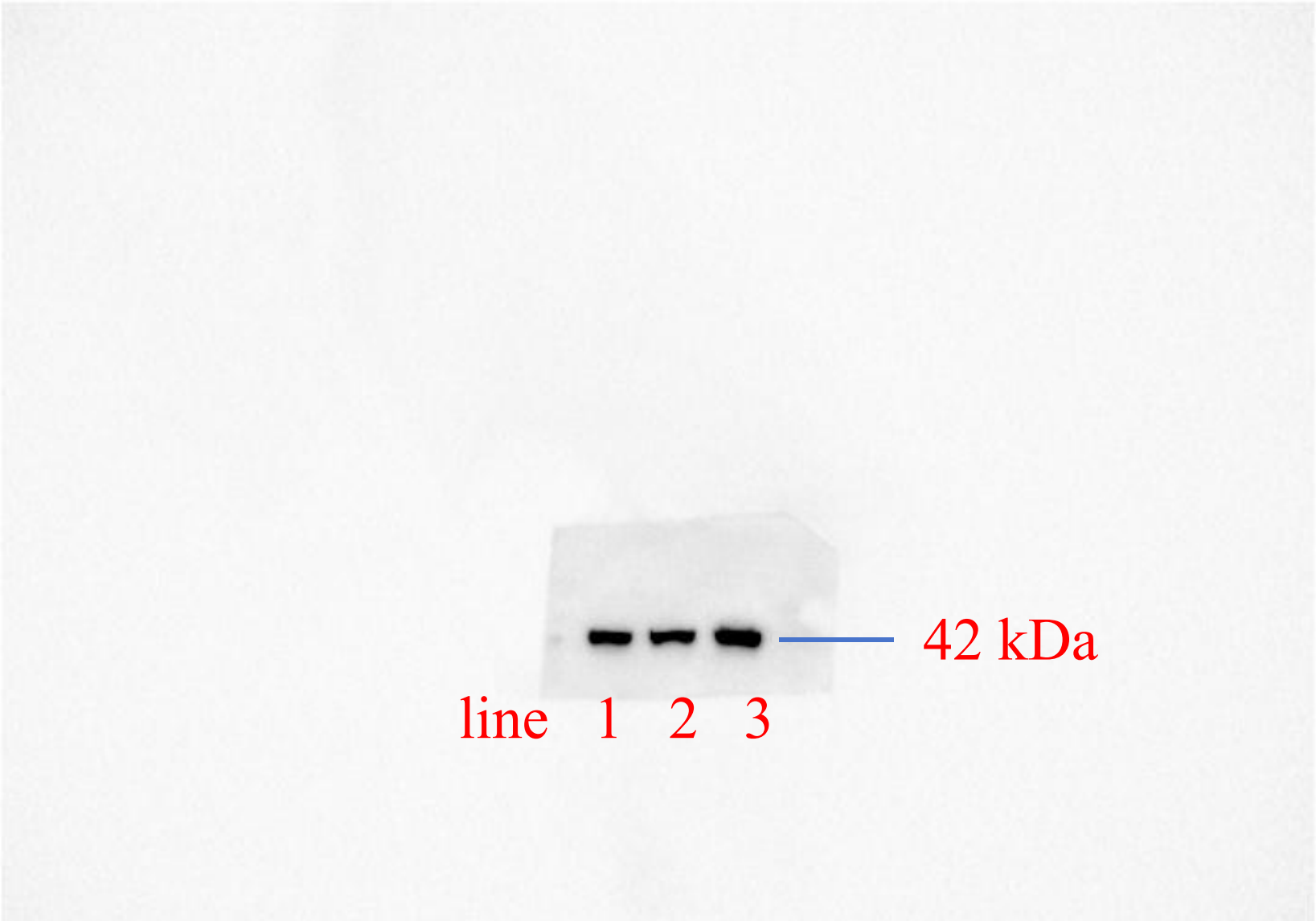

Raw unadjusted, uncropped blot for Figure 3c

Protein: PAK4

Samples loaded order (left to right):

T-24 PpD teatment control(0), 31.3, 62.5, 125, 250, 500, marker, 5637 PpD teatment control(0), 31.3, 62.5, 125, 250, 500

Lanes removed in final figure: Lane 7 (marked ×)

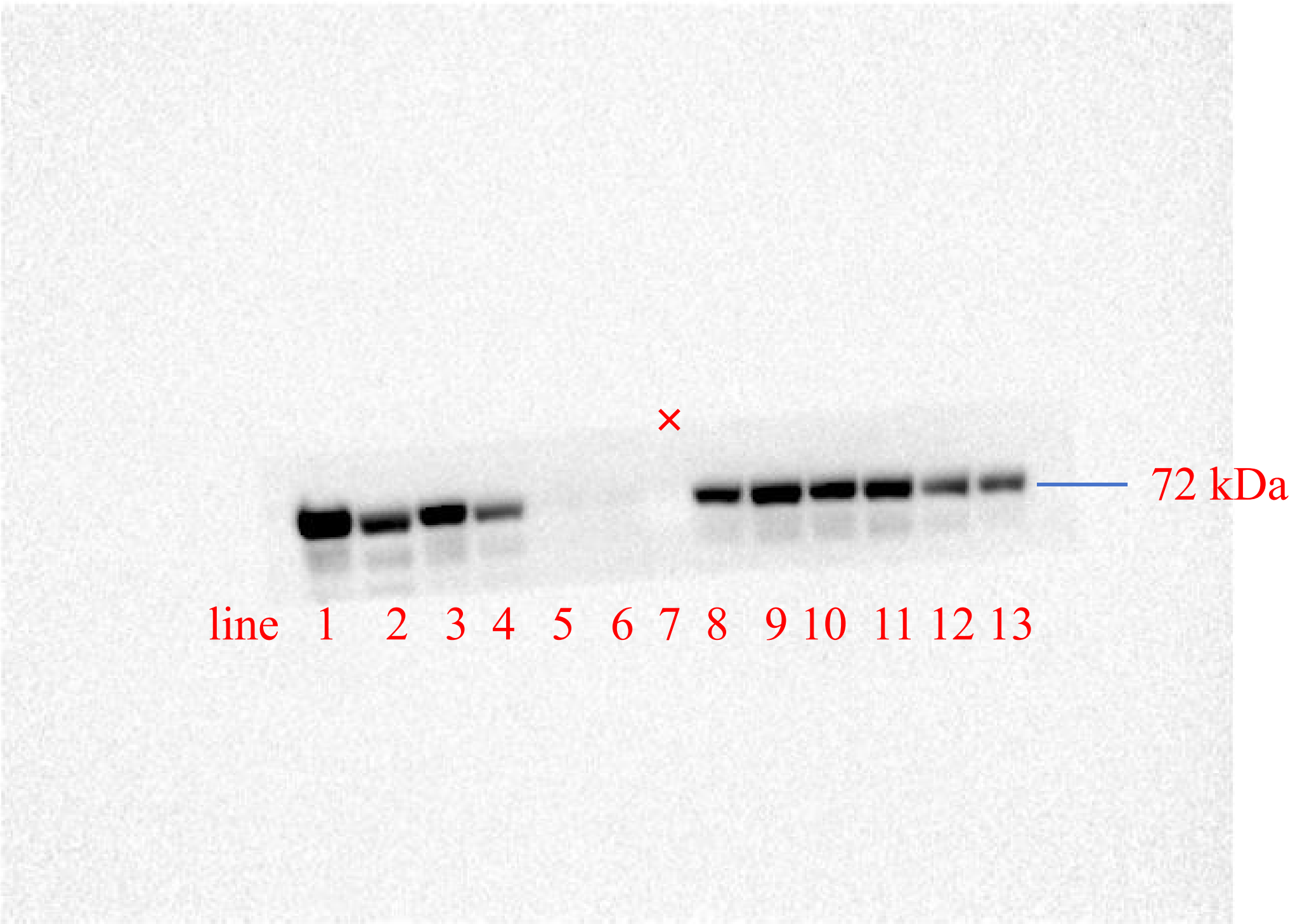

Raw unadjusted, uncropped blot for Figure 3c

Protein: β-actin

Samples loaded order (left to right):

T-24 PpD teatment control(0), 31.3, 62.5, 125, 250, 500, marker, 5637 PpD teatment control(0), 31.3, 62.5, 125, 250, 500

Lanes removed in final figure: Lane 7 (marked ×)

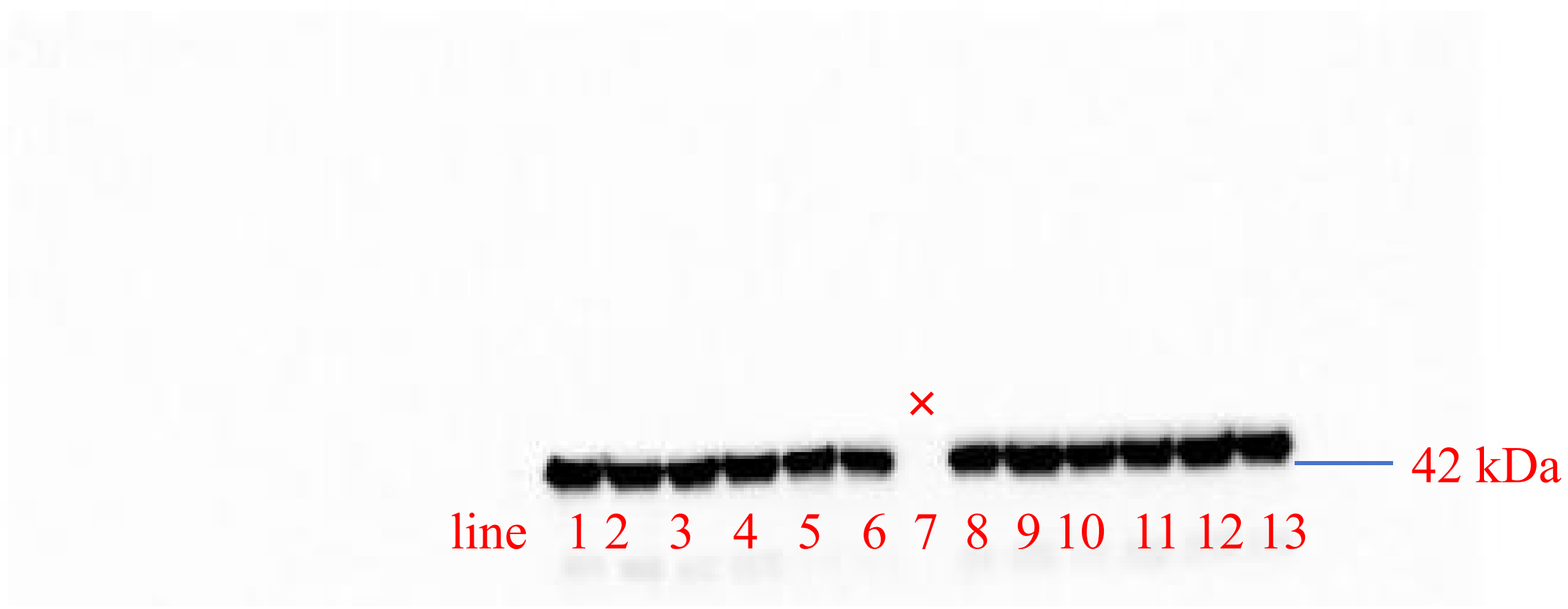

Raw unadjusted, uncropped blot for Figure 4a

Protein: PAK4

Samples loaded order (left to right): T-24 sg-control, T-24 sg-PAK4-10, marker, 5637 sg-control, 5637 sg-PAK4-33

Lanes removed in final figure: Lane 3 (marked ×)

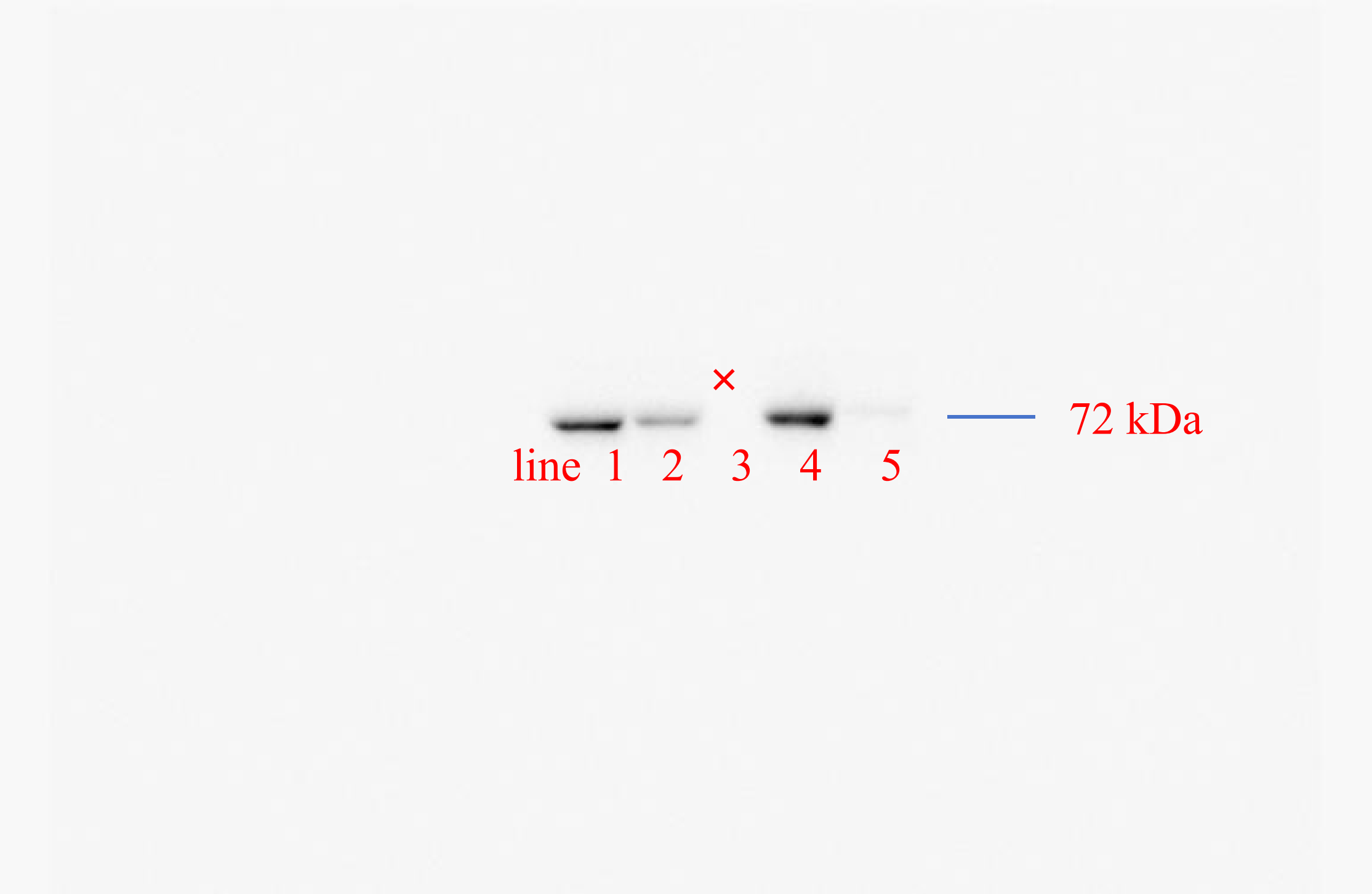

Raw unadjusted, uncropped blot for Figure 4a

Protein: PARP and cleaved PARP

Samples loaded order (left to right): T-24 sg-control, T-24 sg-PAK4-10

Lanes removed in final figure: none

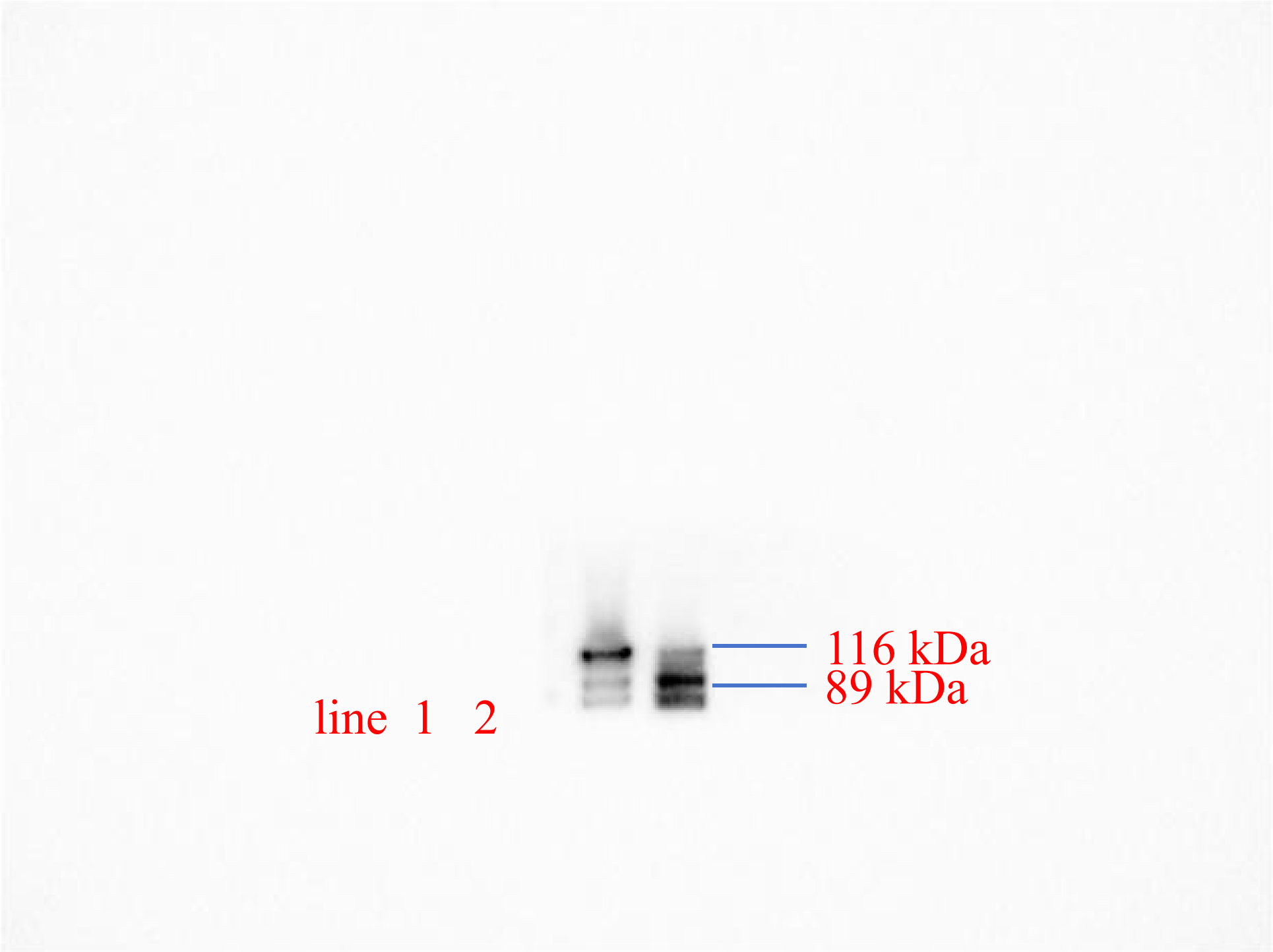

Raw unadjusted, uncropped blot for Figure 4a

Protein: PARP and cleaved PARP

Samples loaded order (left to right): 5637 sg-control, 5637 sg-PAK4-10

Lanes removed in final figure: none

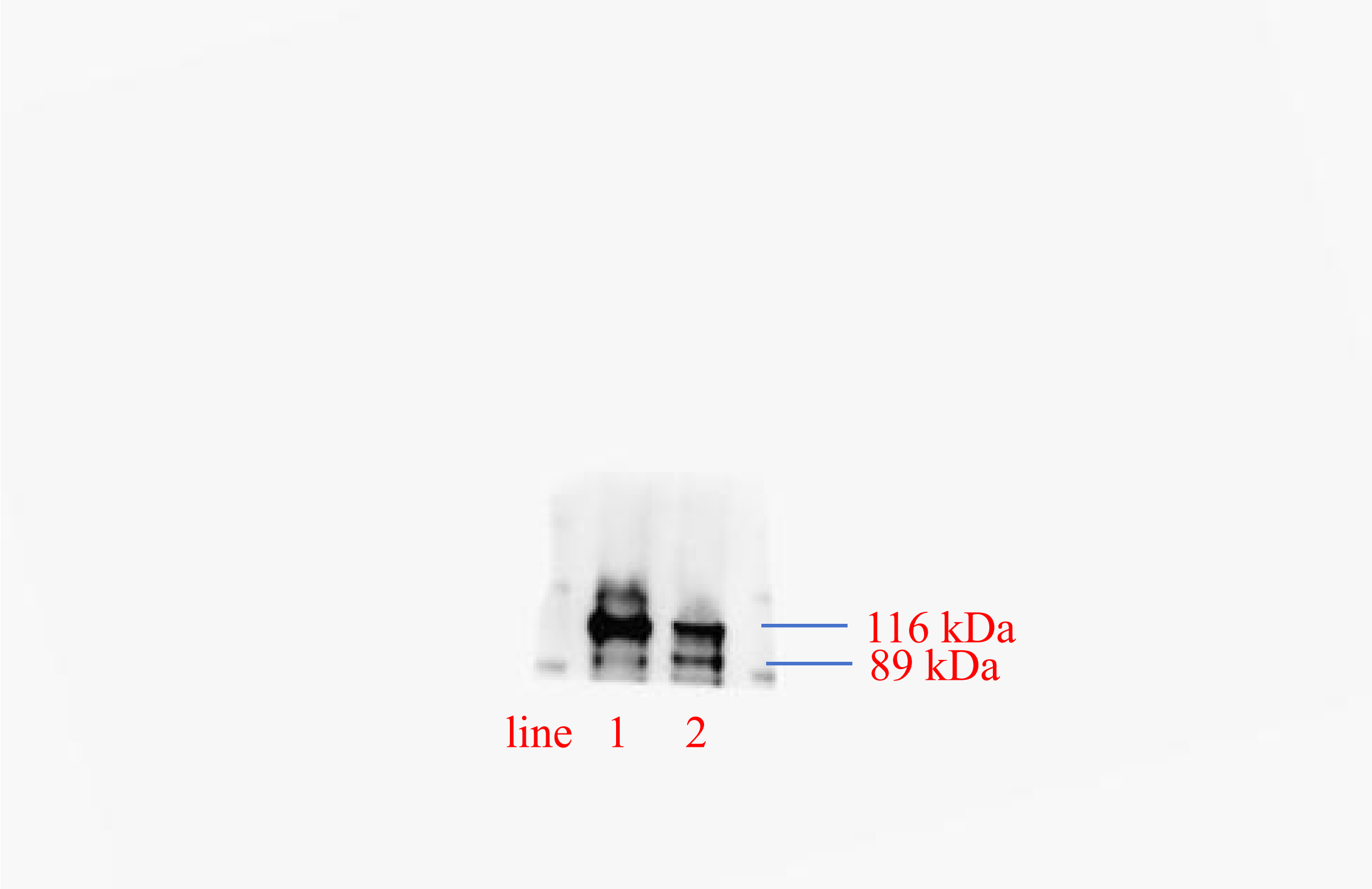

Raw unadjusted, uncropped blot for Figure 4a

Protein: caspase 3

Samples loaded order (left to right): T-24 sg-control, T-24 sg-PAK4-10, 5637 sg-control, 5637 sg-PAK4-33

Lanes removed in final figure: none

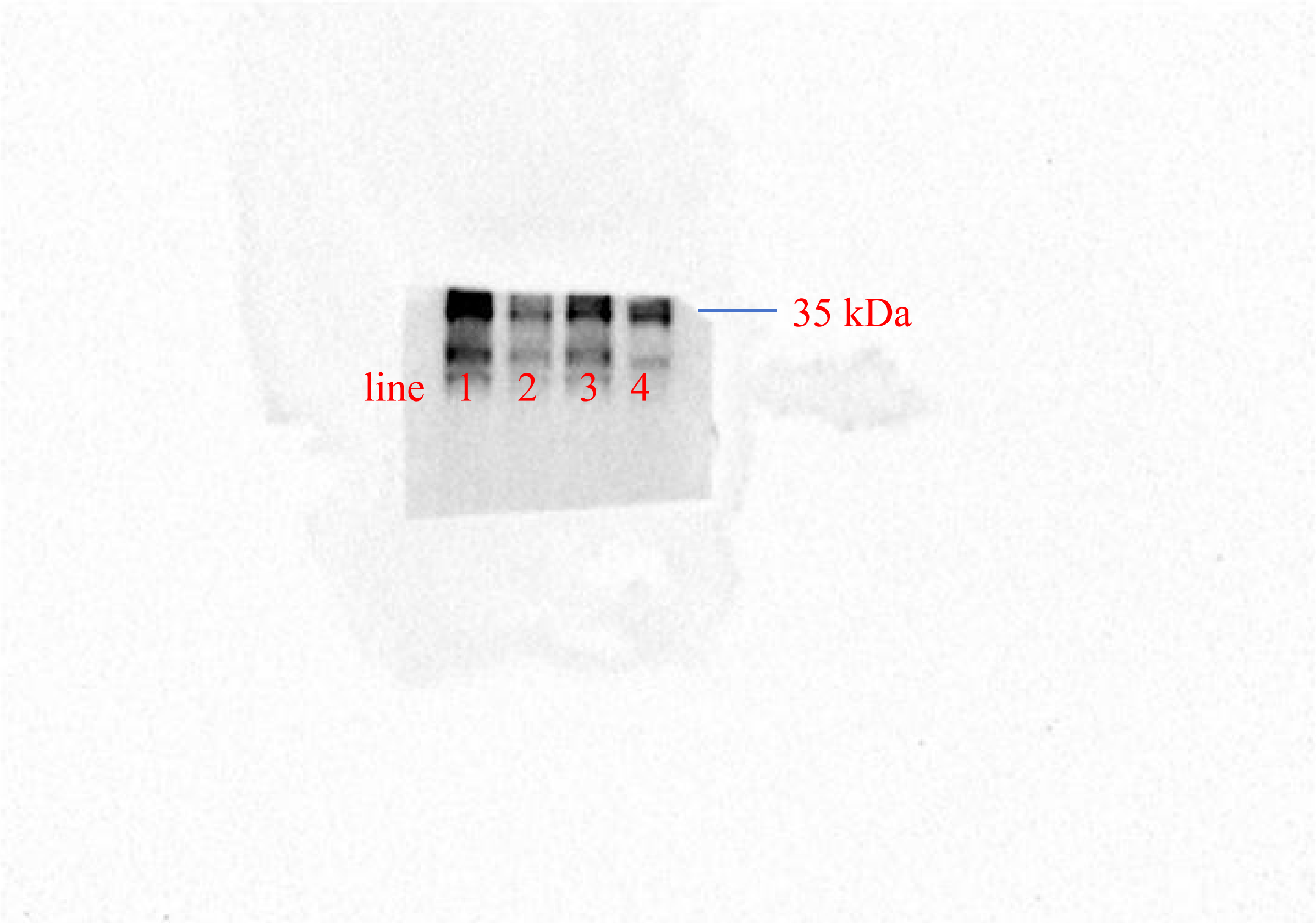

Raw unadjusted, uncropped blot for Figure 4a

Protein: Erk

Samples loaded order (left to right): T-24 sg-control, T-24 sg-PAK4-10, marker, 5637 sg-control, 5637 sg-PAK4-33

Lanes removed in final figure: Lane 3 (marked ×)

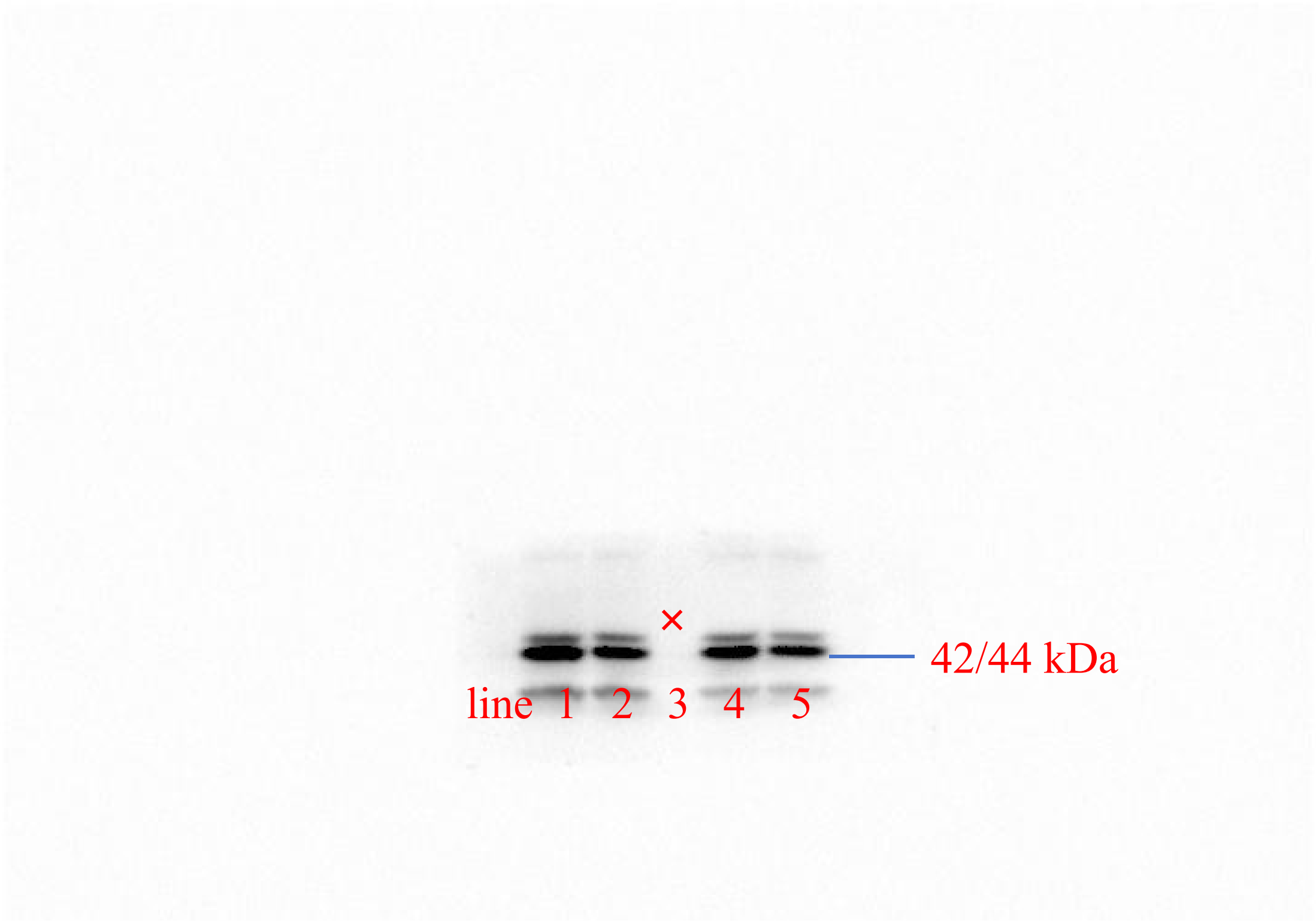

Raw unadjusted, uncropped blot for Figure 4a

Protein: p-Erk

Samples loaded order (left to right): T-24 sg-control, T-24 sg-PAK4-10, marker, 5637 sg-control, 5637 sg-PAK4-33

Lanes removed in final figure: Lane 3 (marked ×)

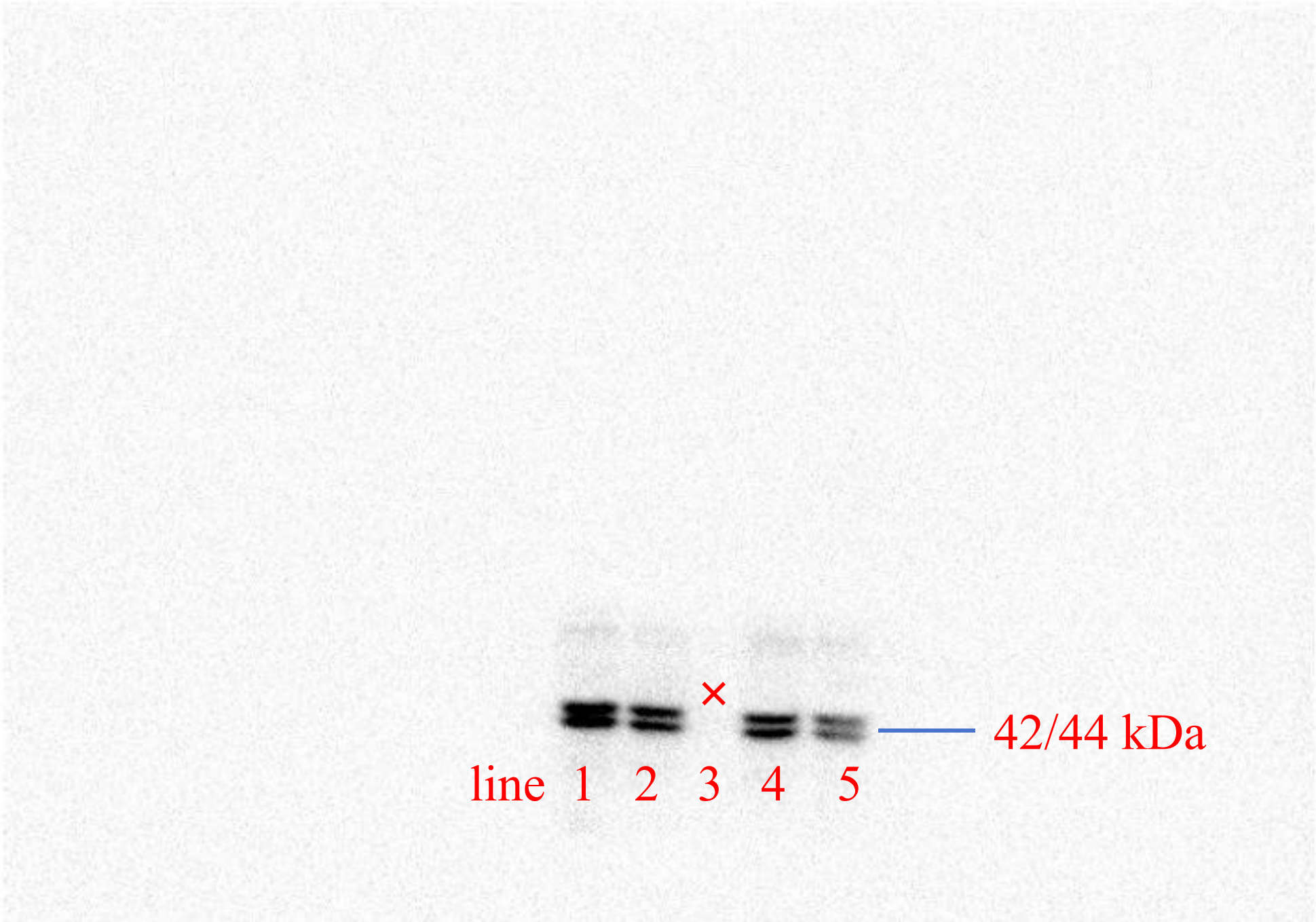

Raw unadjusted, uncropped blot for Figure 4a

Protein: CDK4

Samples loaded order (left to right): T-24 sg-control, T-24 sg-PAK4-10, marker, 5637 sg-control, 5637 sg-PAK4-33

Lanes removed in final figure: Lane 3 (marked ×)

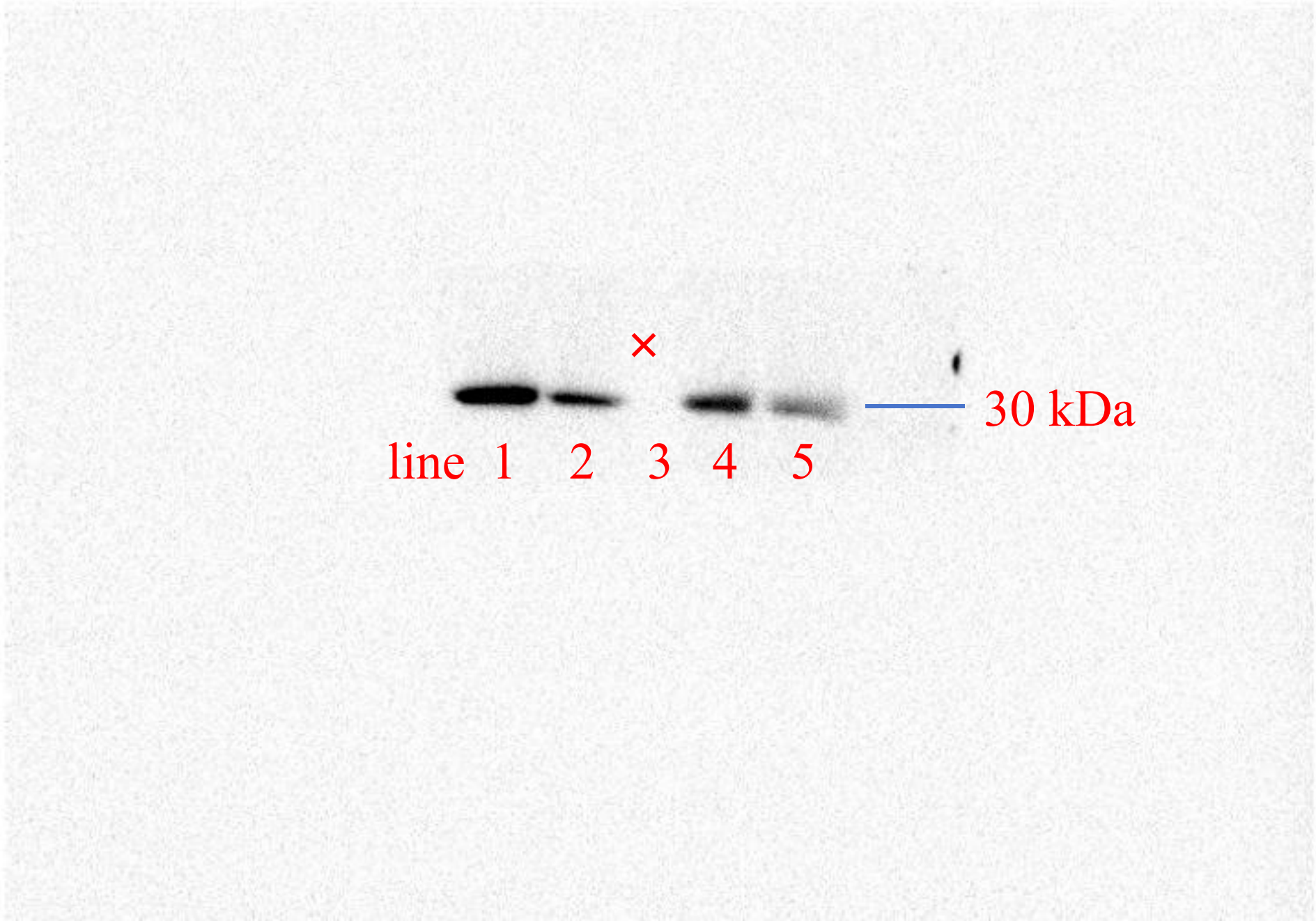

Raw unadjusted, uncropped blot for Figure 4a

Protein: cyclin D1

Samples loaded order (left to right): T-24 sg-control, T-24 sg-PAK4-10, marker, 5637 sg-control, 5637 sg-PAK4-33

Lanes removed in final figure: Lane 3 (marked ×)

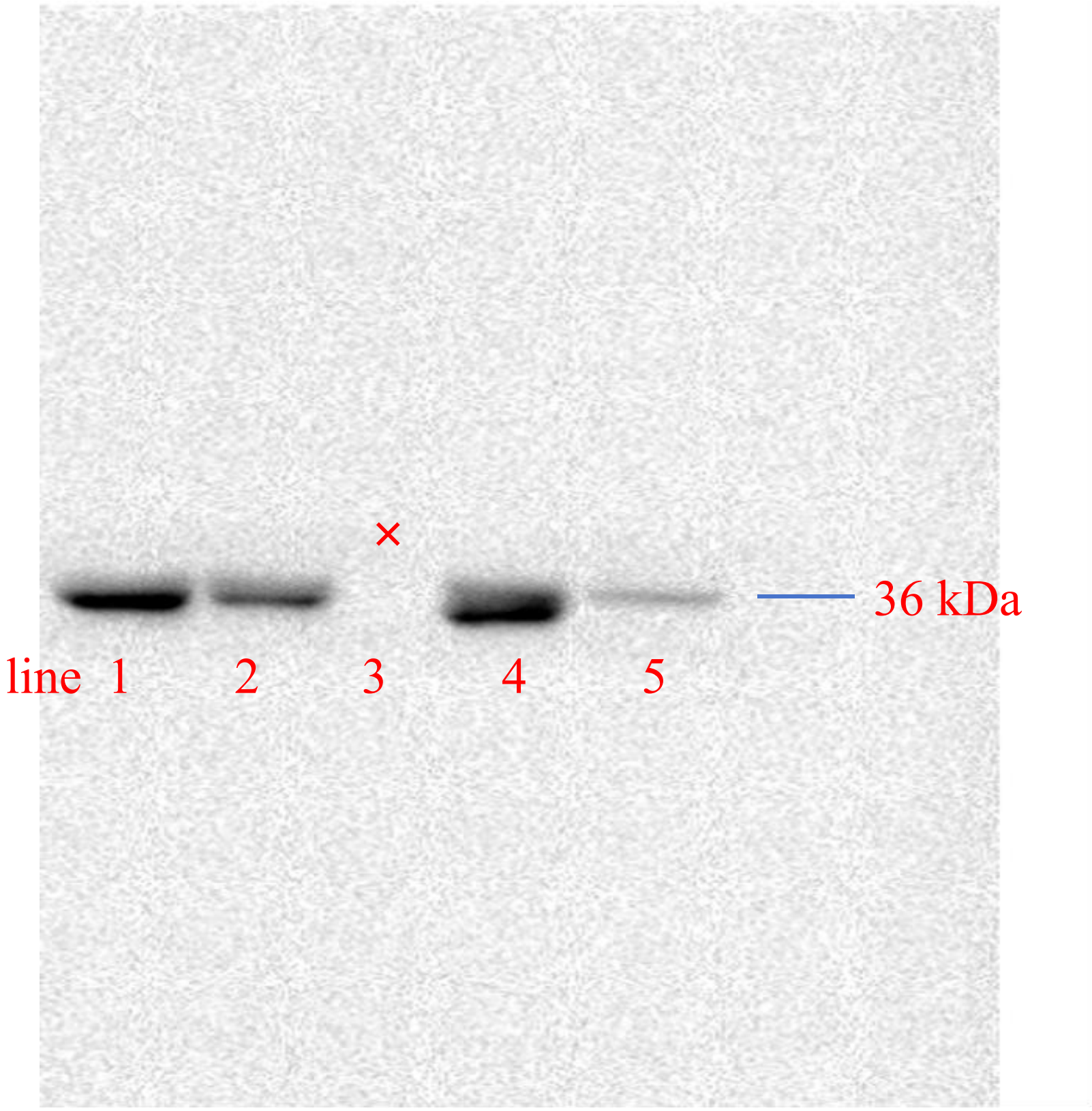

Raw unadjusted, uncropped blot for Figure 4a

Protein: PCNA

Samples loaded order (left to right): T-24 sg-control, T-24 sg-PAK4-10

Lanes removed in final figure: none

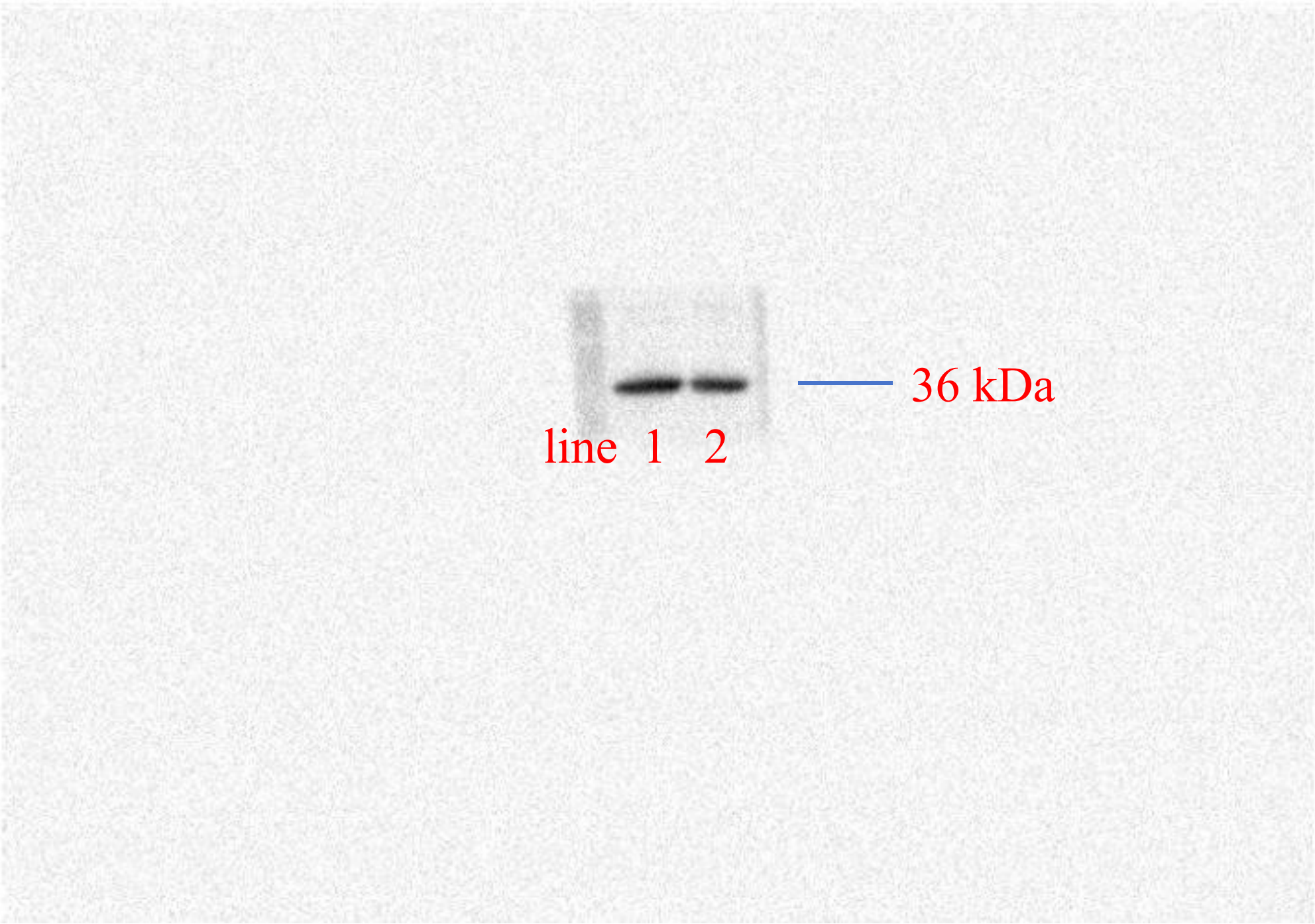

Raw unadjusted, uncropped blot for Figure 4a

Protein: PCNA

Samples loaded order (left to right): 5637 sg-control, 5637 sg-PAK4-33

Lanes removed in final figure: none

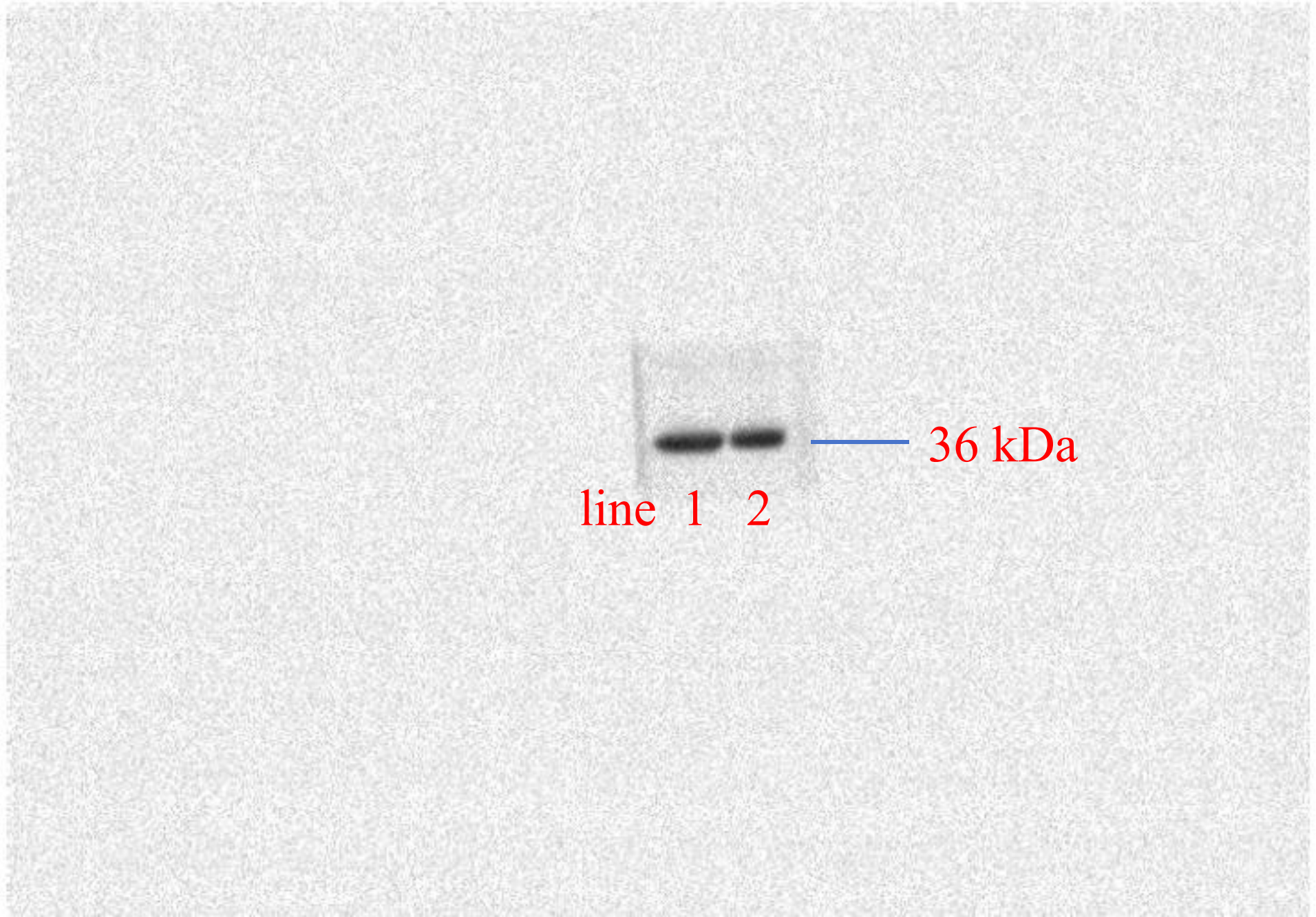

Raw unadjusted, uncropped blot for Figure 4a

Protein: vinculin

Samples loaded order (left to right): T-24 sg-control, T-24 sg-PAK4-10

Lanes removed in final figure: none

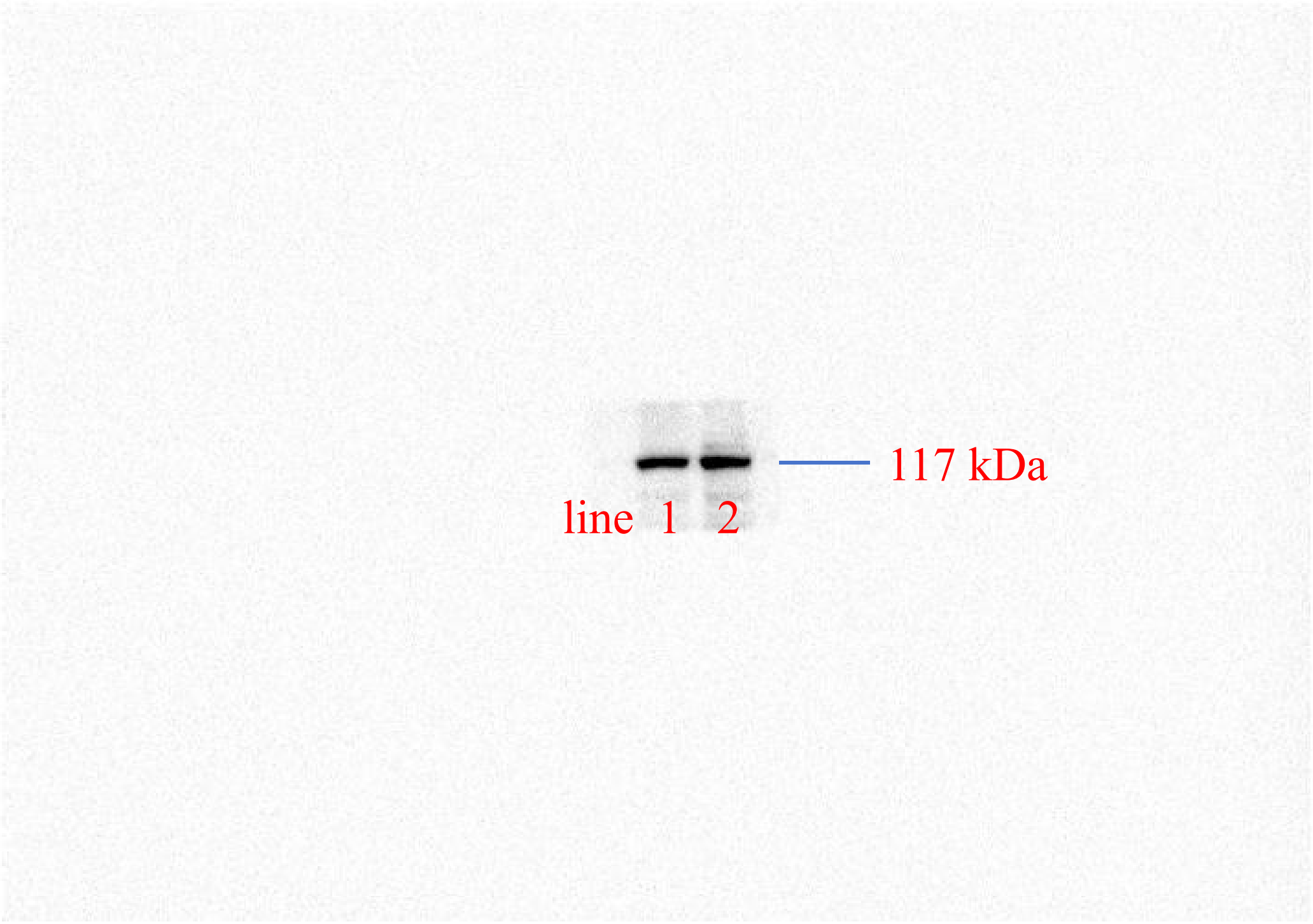

Raw unadjusted, uncropped blot for Figure 4a

Protein: vinculin

Samples loaded order (left to right): 5637 sg-control, 5637 sg-PAK4-33

Lanes removed in final figure: none

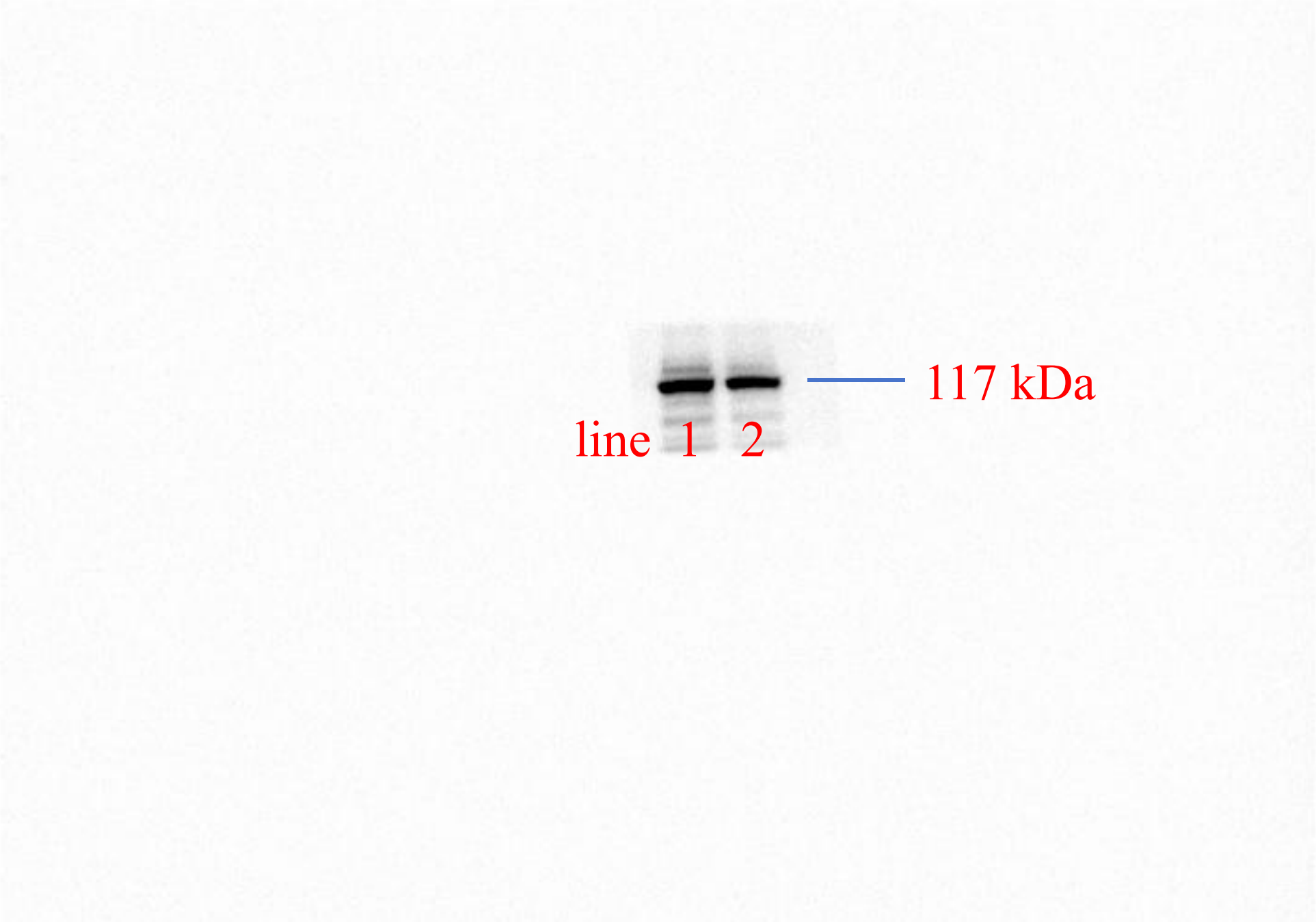

Raw unadjusted, uncropped blot for Figure 4b

Protein: PAK4

Samples loaded order (left to right):

T-24 PpD treatment: control (0), 62, 125, 250 nM (line 1-4)

Lanes removed in final figure: none

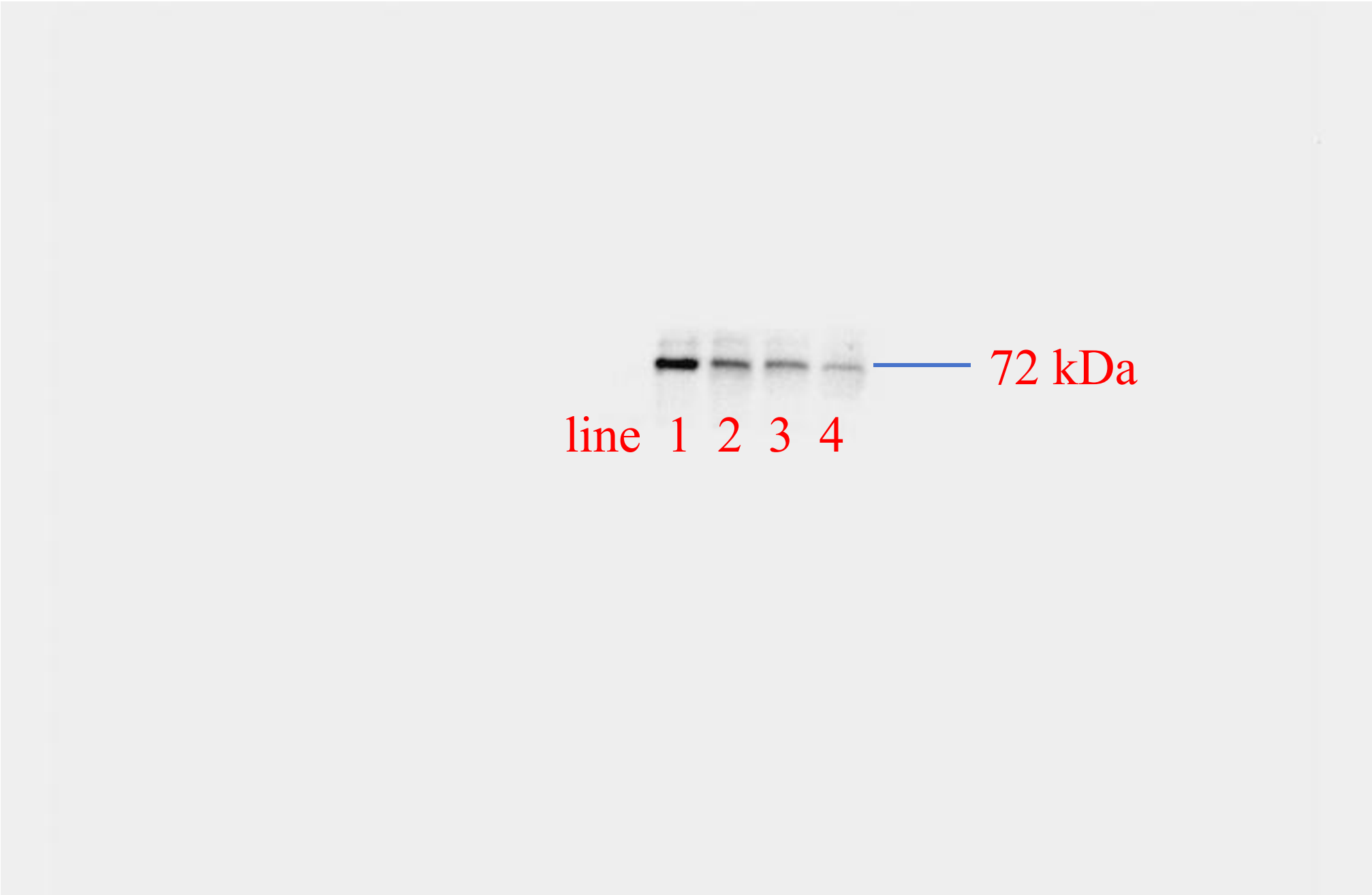

Raw unadjusted, uncropped blot for Figure 4b

Protein: PAK4

Samples loaded order (left to right):

5637 PpD treatment: control (0), 62, 125, 250 nM (line 1-4)

Lanes removed in final figure: none

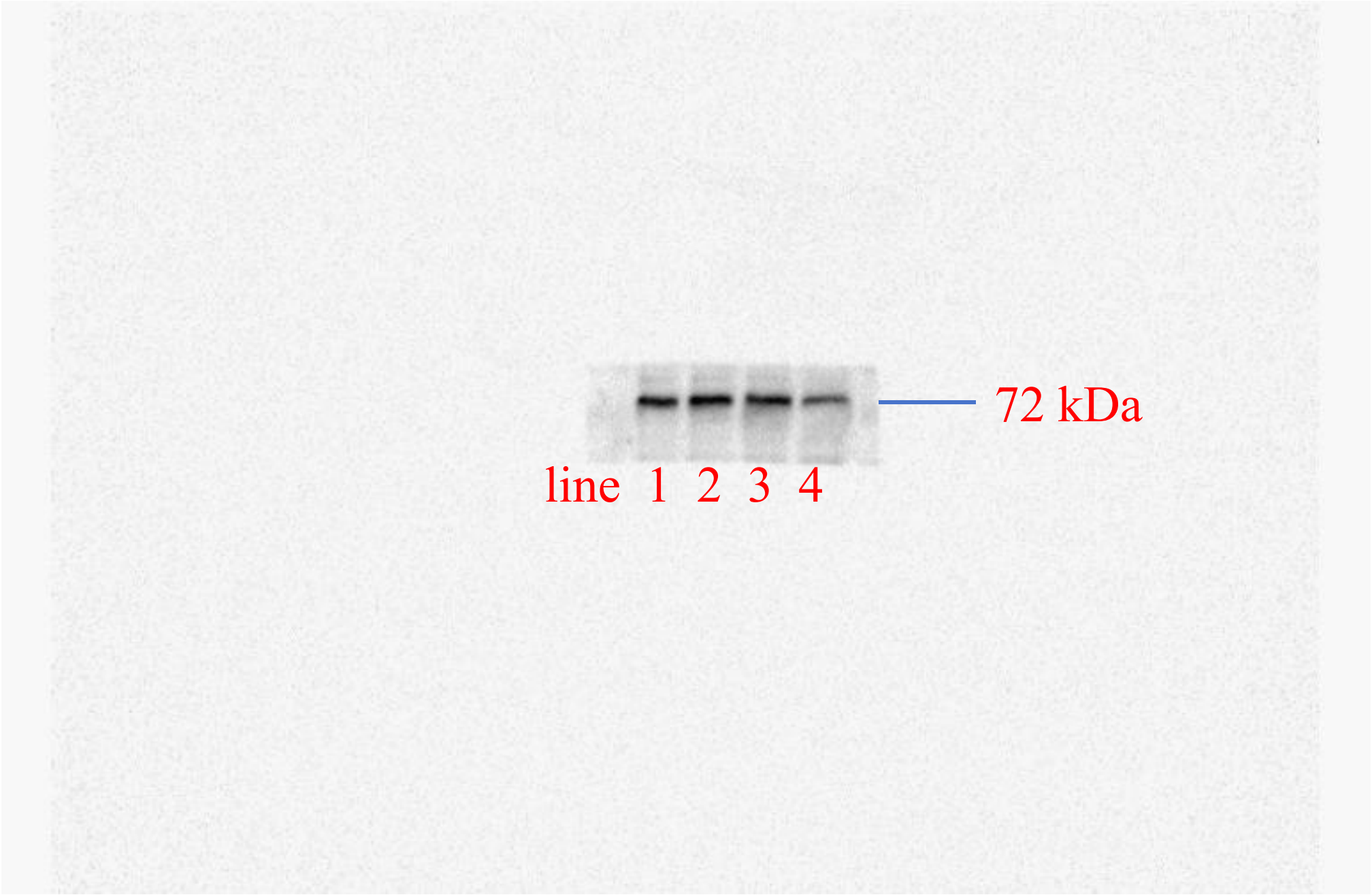

Raw unadjusted, uncropped blot for Figure 4b

Protein: PARP

Samples loaded order (left to right):

T-24 PpD treatment: control (0), 62, 125, 250 nM (line 1-4)

Lanes removed in final figure: none

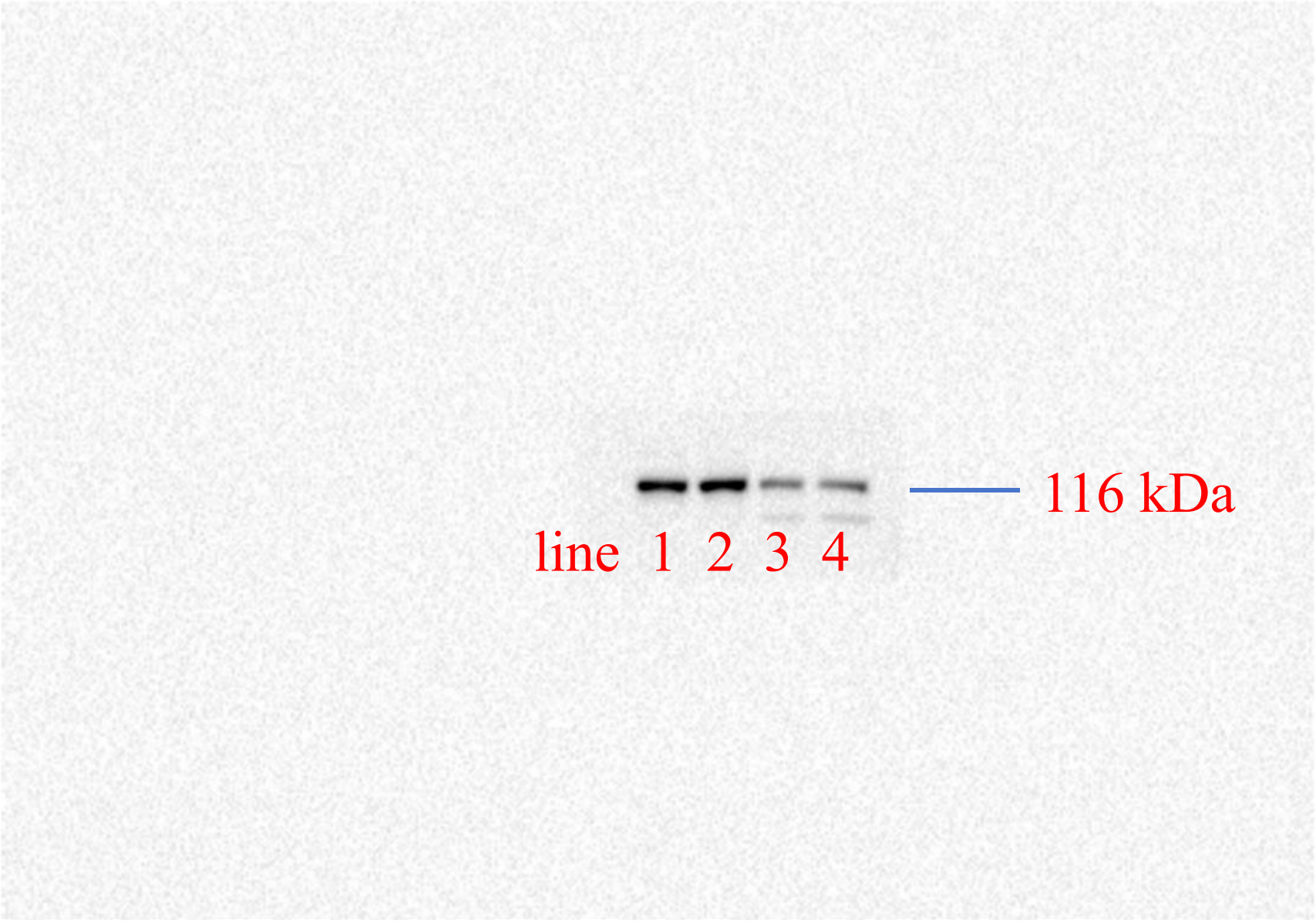

Raw unadjusted, uncropped blot for Figure 4b

Protein: PARP

Samples loaded order (left to right):

5637 PpD treatment: control (0), 62, 125, 250 nM (line 1-4)

Lanes removed in final figure: none

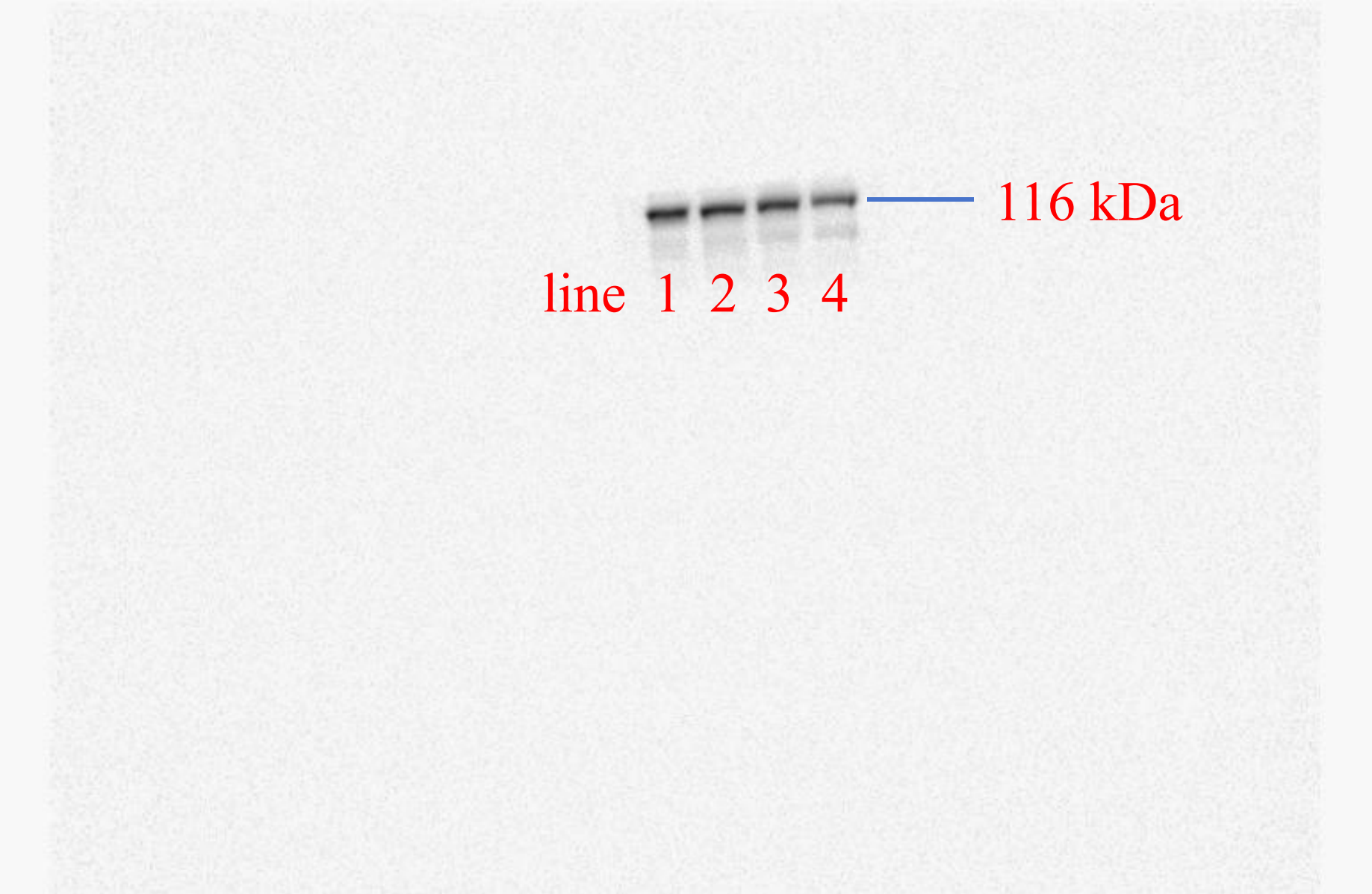

Raw unadjusted, uncropped blot for Figure 4b

Protein: cleaved PARP

Samples loaded order (left to right):

T-24 PpD treatment: control (0), 62, 125, 250 nM (line 1-4)

Lanes removed in final figure: none

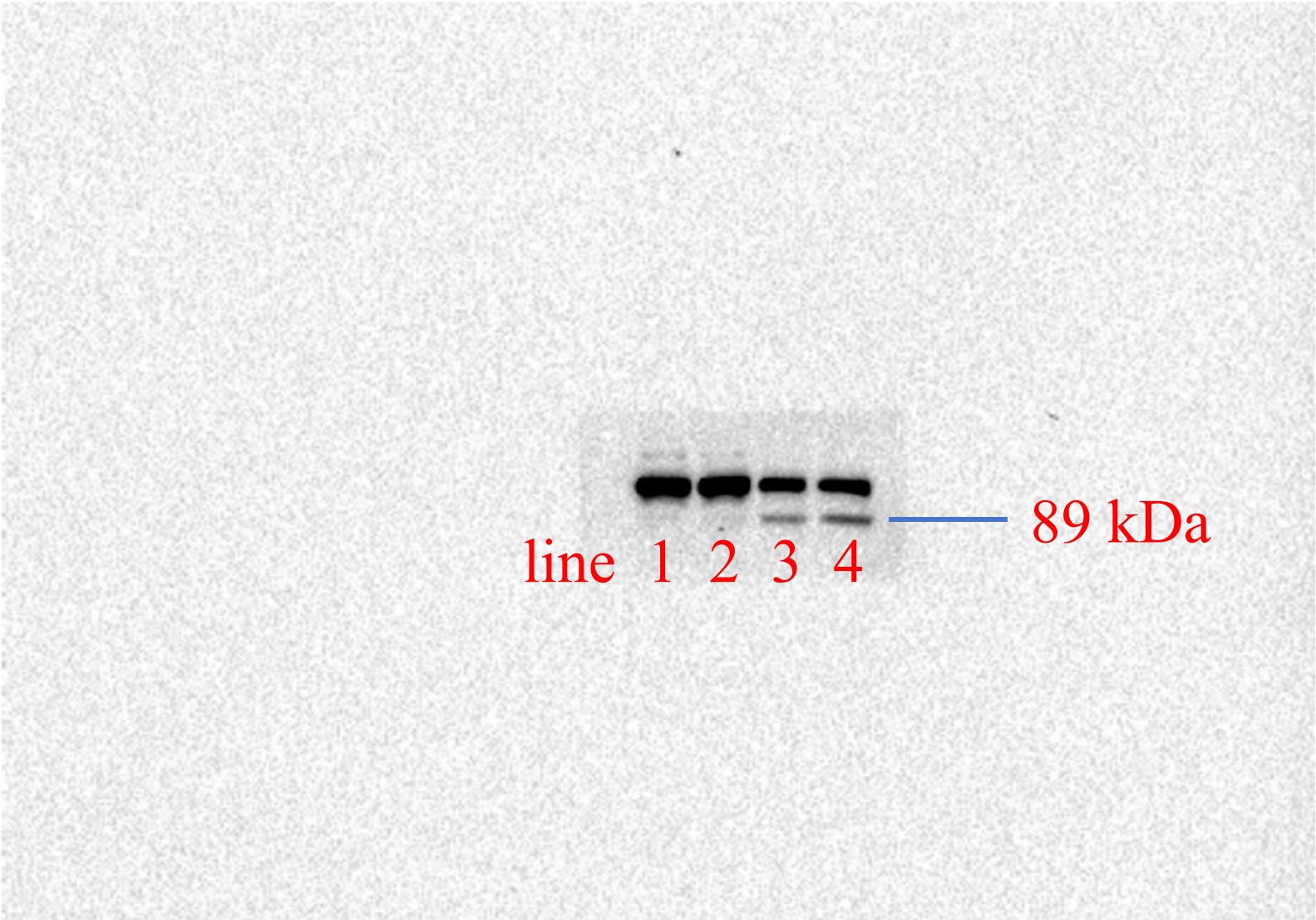

Raw unadjusted, uncropped blot for Figure 4b

Protein: cleaved PARP

Samples loaded order (left to right):

5637 PpD treatment: control (0), 62, 125, 250 nM (line 1-4)

Lanes removed in final figure: none

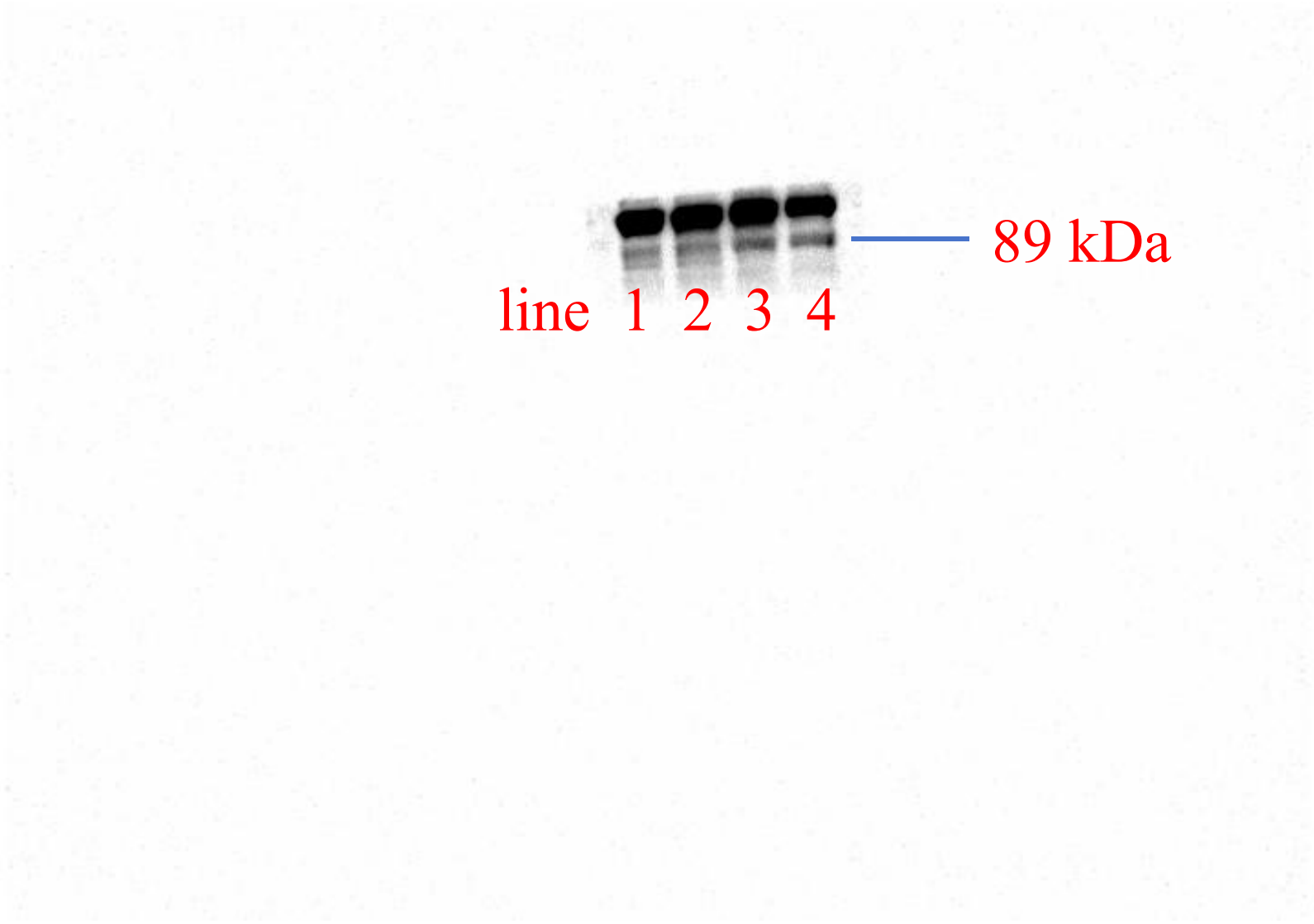

Raw unadjusted, uncropped blot for Figure 4b (T-24)

Protein: Caspase 3

Samples loaded order (left to right):

5637 PpD treatment: control (0), 62, 125, 250 nM, marker, T-24 PpD treatment: control (0), 62, 125, 250 nM (line 1-9)

Lanes removed in final figure: Lane 1-5 (marked ×)

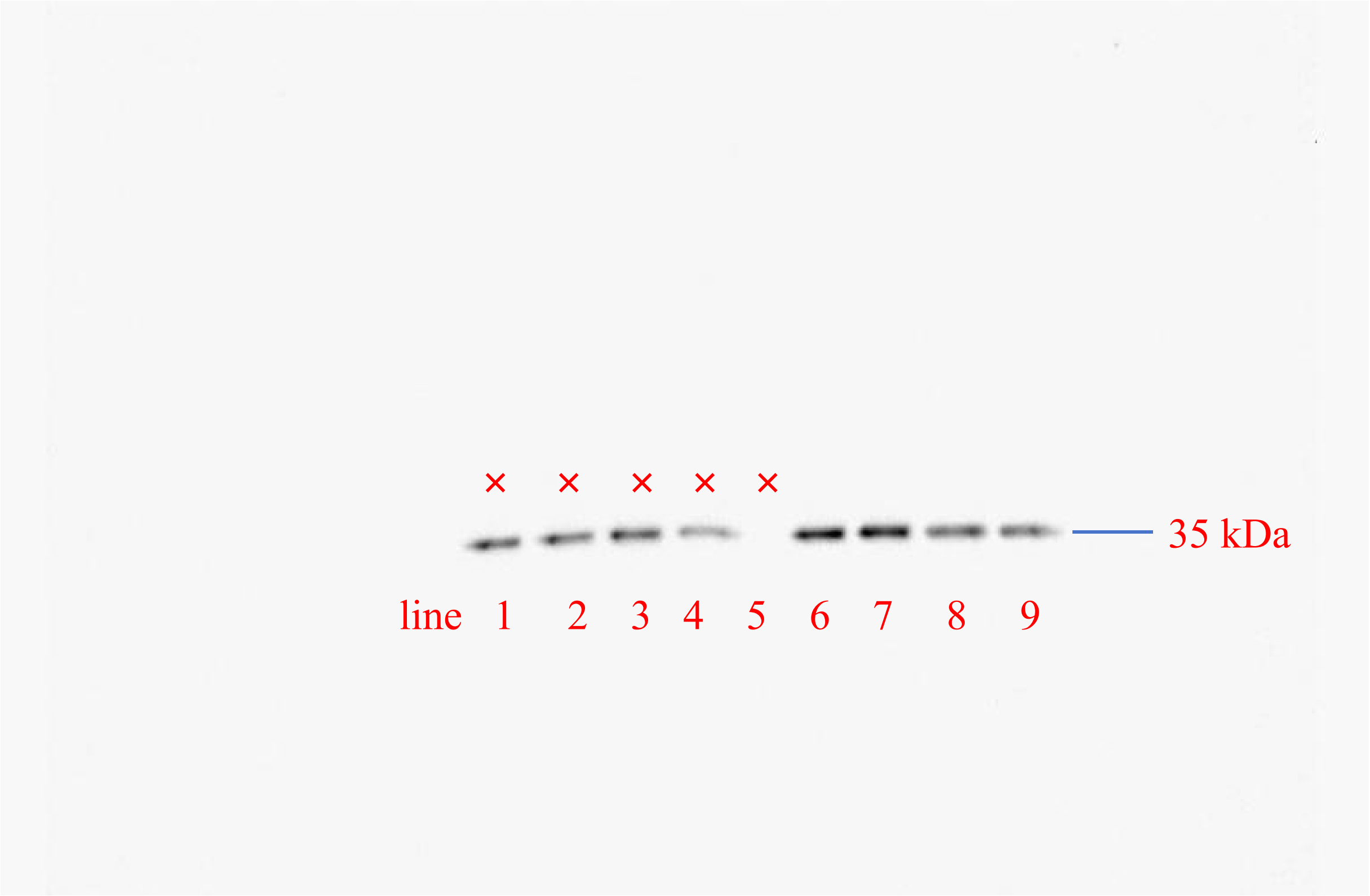

Raw unadjusted, uncropped blot for Figure 4b (5637)

Protein: Caspase 3

Samples loaded order (left to right):

5637 PpD treatment: control (0), 62, 125, 250 nM, marker, T-24 PpD treatment: control (0), 62, 125, 250 nM (line 1-9)

Lanes removed in final figure: Lane 5-9 (marked ×)

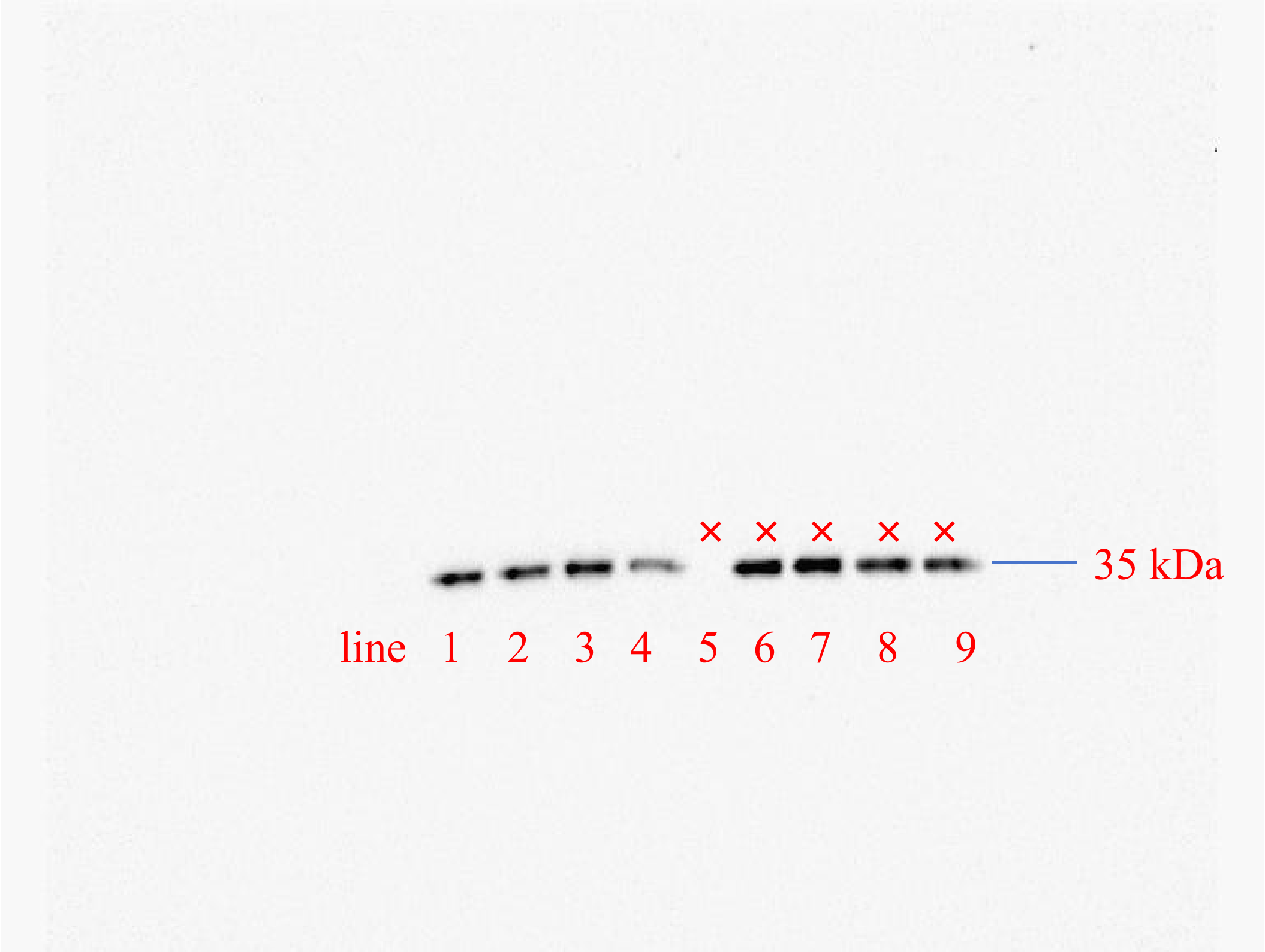

Raw unadjusted, uncropped blot for Figure 4b (T-24)

Protein: Erk

Samples loaded order (left to right):

T-24 PpD treatment: control (0), 62, 125, 250 nM (line 1-4)

Lanes removed in final figure: None.

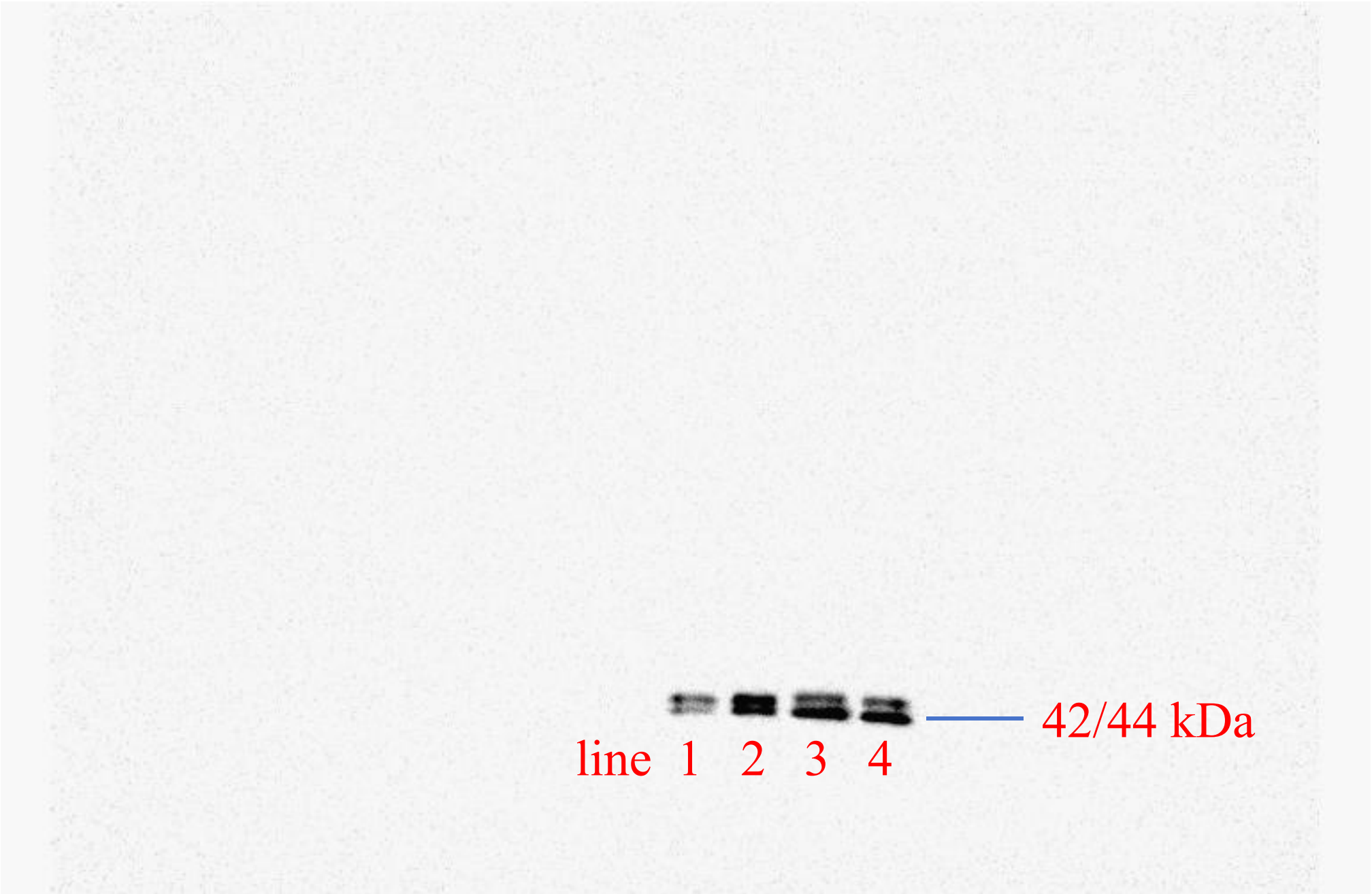

Raw unadjusted, uncropped blot for Figure 4b (5637)

Protein: Erk

Samples loaded order (left to right):

5637 PpD treatment: control (0), 62, 125, 250 nM (line 1-4)

Lanes removed in final figure: None.

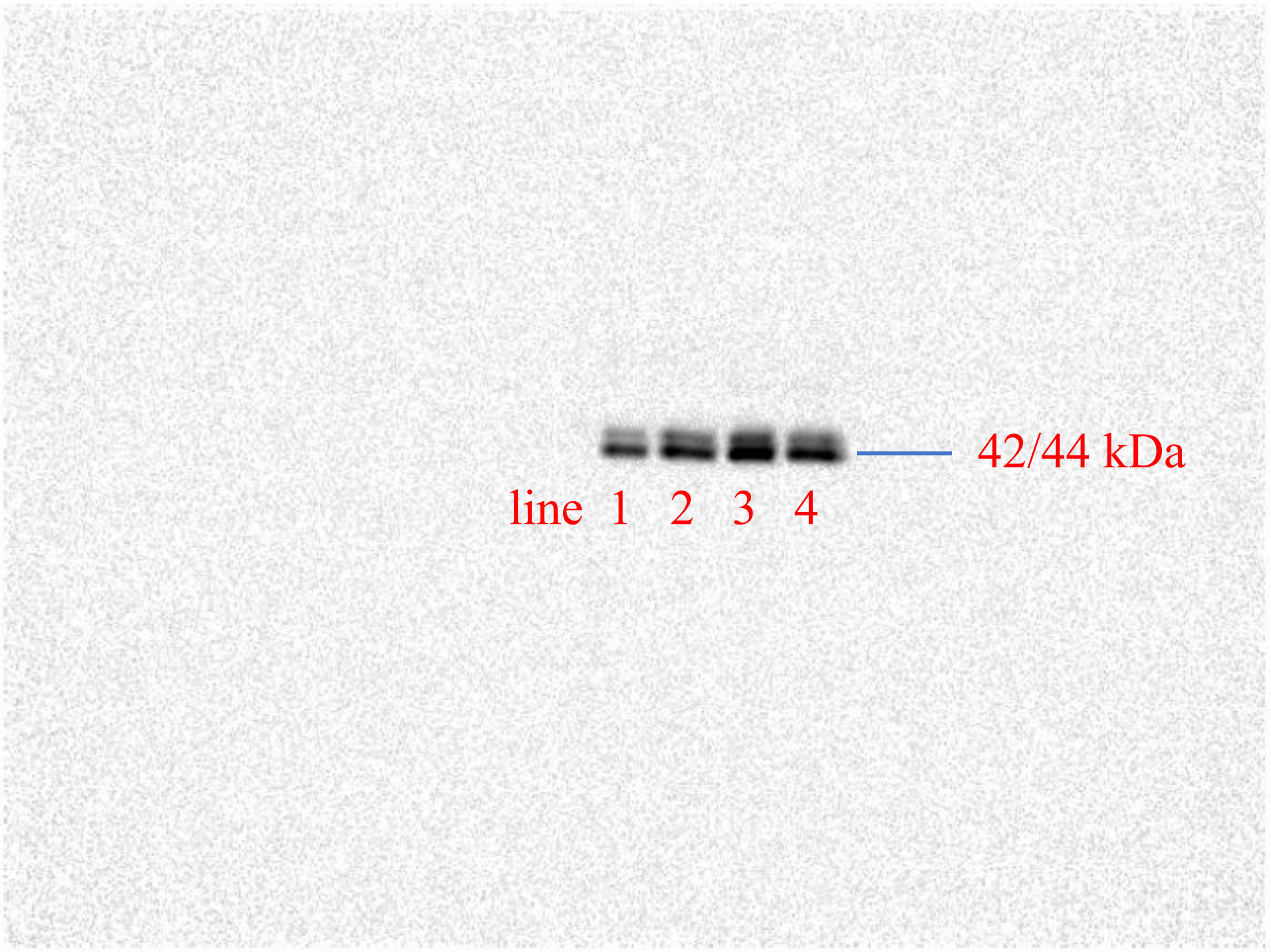

Raw unadjusted, uncropped blot for Figure 4b (T-24)

Protein: p-Erk

Samples loaded order (left to right):

5637 PpD treatment: control (0), 62, 125, 250 nM, marker, T-24 PpD treatment: control (0), 62, 125, 250 nM (line 1-9)

Lanes removed in final figure: Lane 1-5 (marked ×)

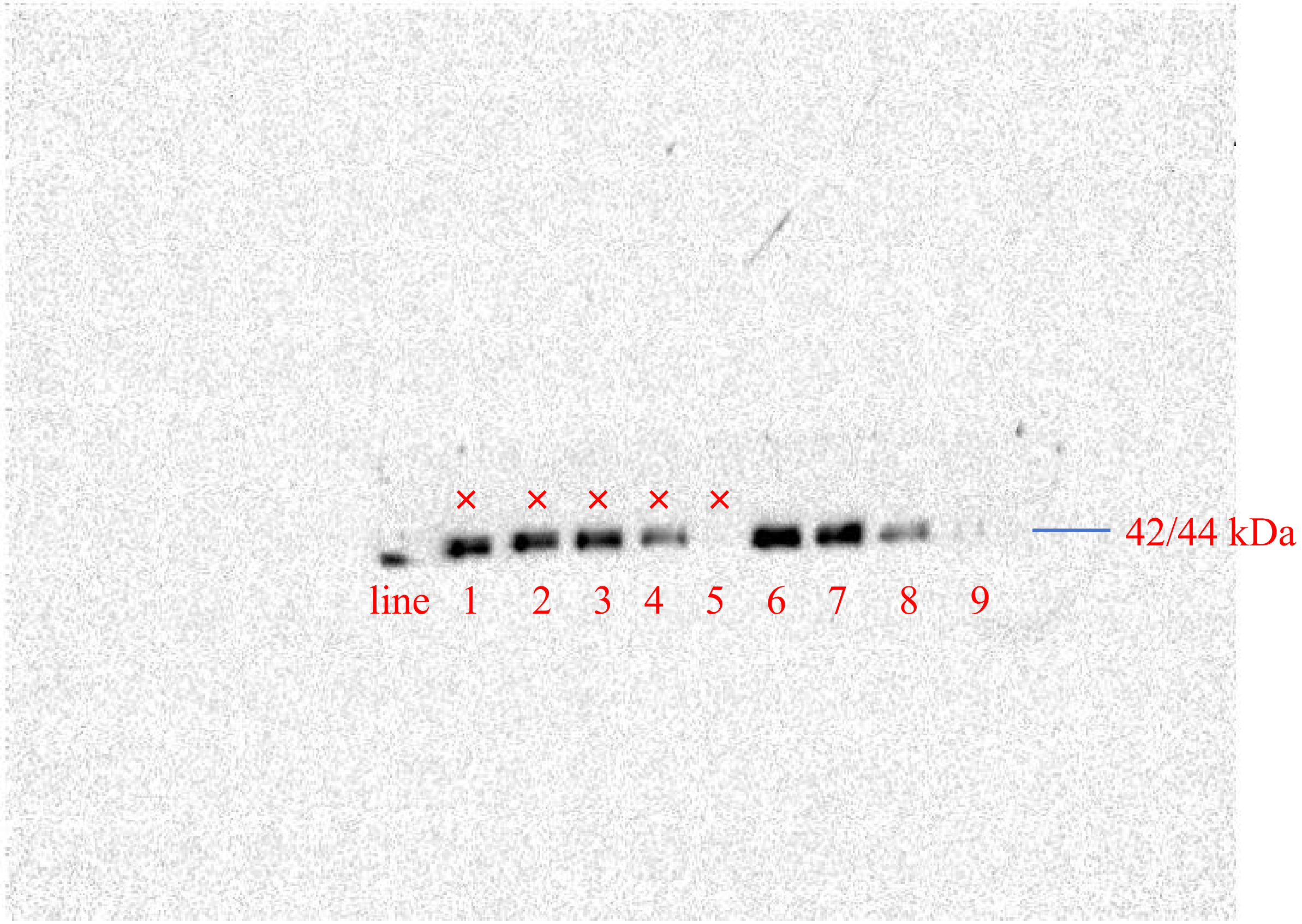

Raw unadjusted, uncropped blot for Figure 4b (T-24)

Protein: p-Erk

Samples loaded order (left to right):

5637 PpD treatment: control (0), 62, 125, 250 nM, marker, T-24 PpD treatment: control (0), 62, 125, 250 nM (line 1-9)

Lanes removed in final figure: Lane 5-9 (marked ×)

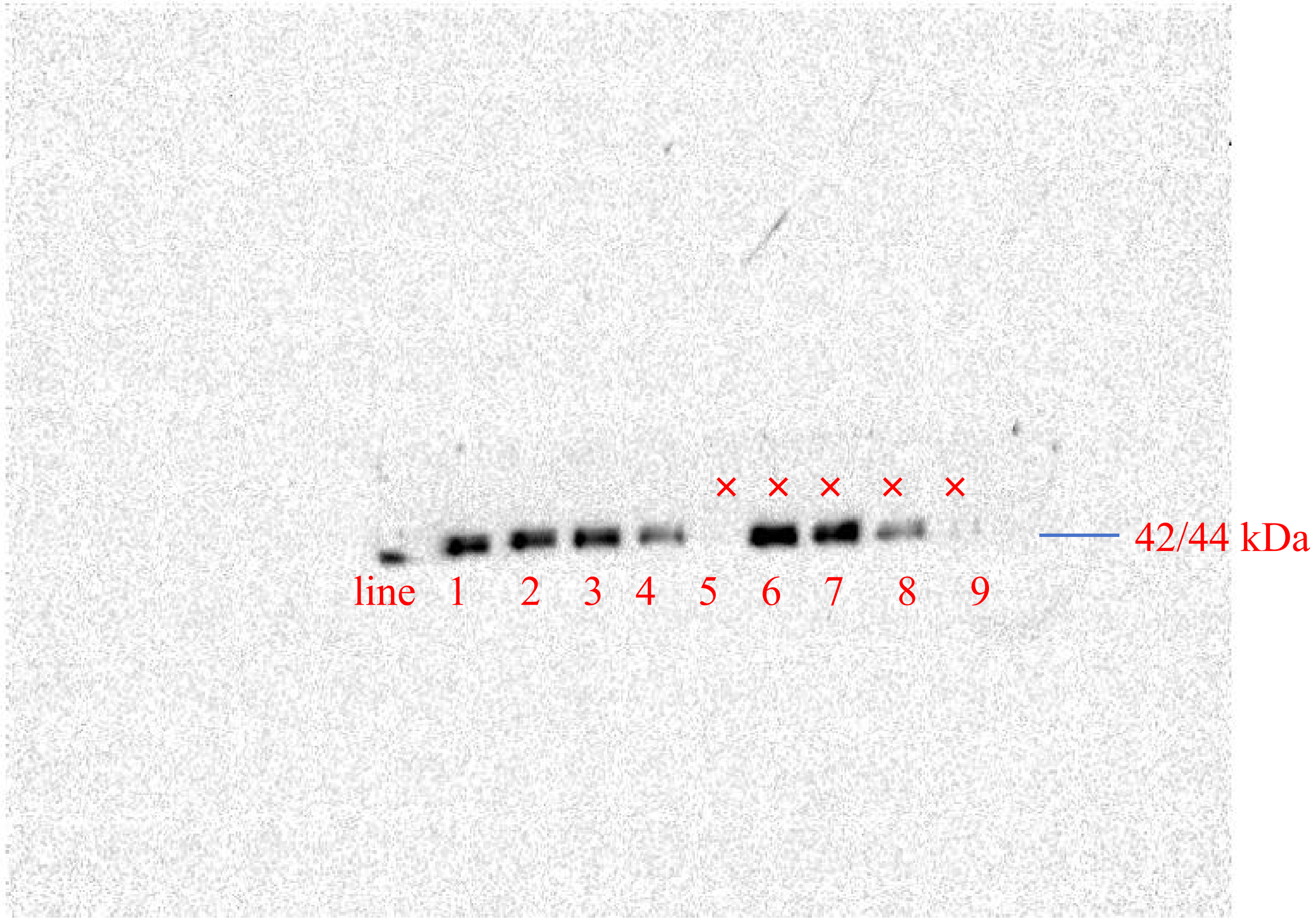

Raw unadjusted, uncropped blot for Figure 4b (T-24)

Protein: CDK4

Samples loaded order (left to right):

T-24 PpD treatment: control (0), 62, 125, 250 nM (line 1-4)

Lanes removed in final figure: None.

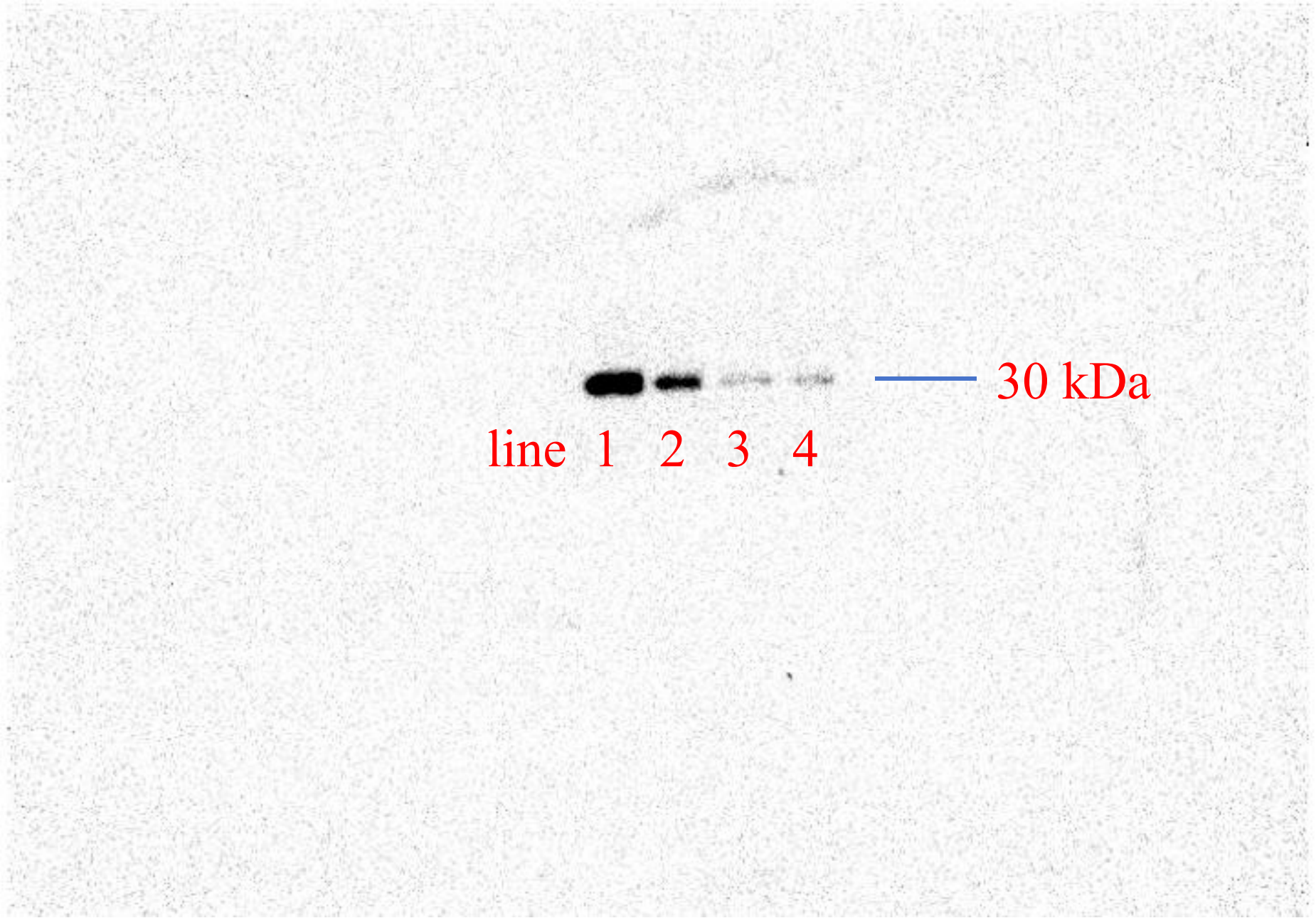

Raw unadjusted, uncropped blot for Figure 4b (5637)

Protein: CDK4

Samples loaded order (left to right):

5637 PpD treatment: control (0), 62, 125, 250 nM (line 1-4)

Lanes removed in final figure: None.

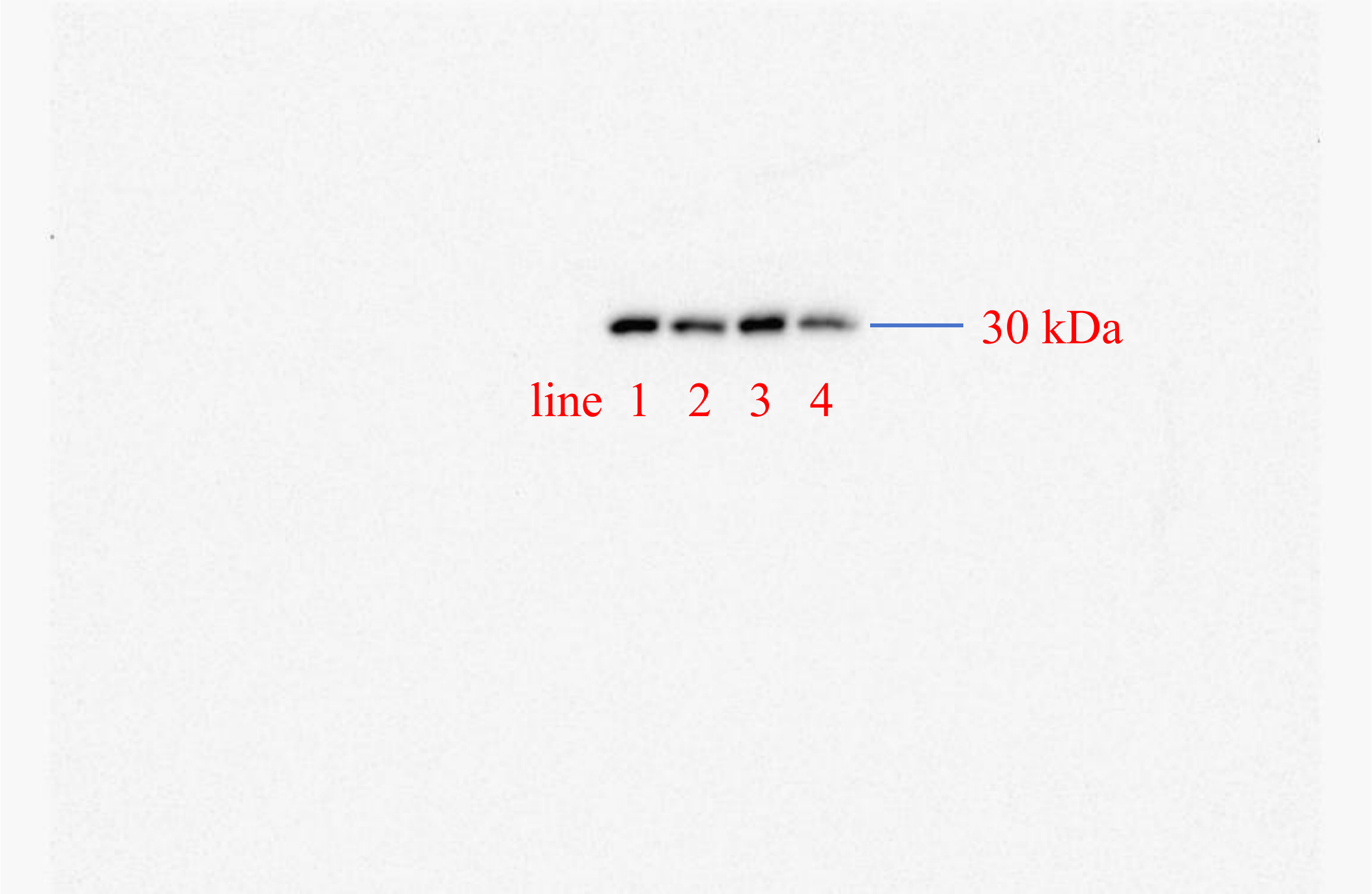

Raw unadjusted, uncropped blot for Figure 4b (T-24)

Protein: Cyclin D1

Samples loaded order (left to right):

T-24 PpD treatment: control (0), 62, 125, 250 nM (line 1-4)

Lanes removed in final figure: None.

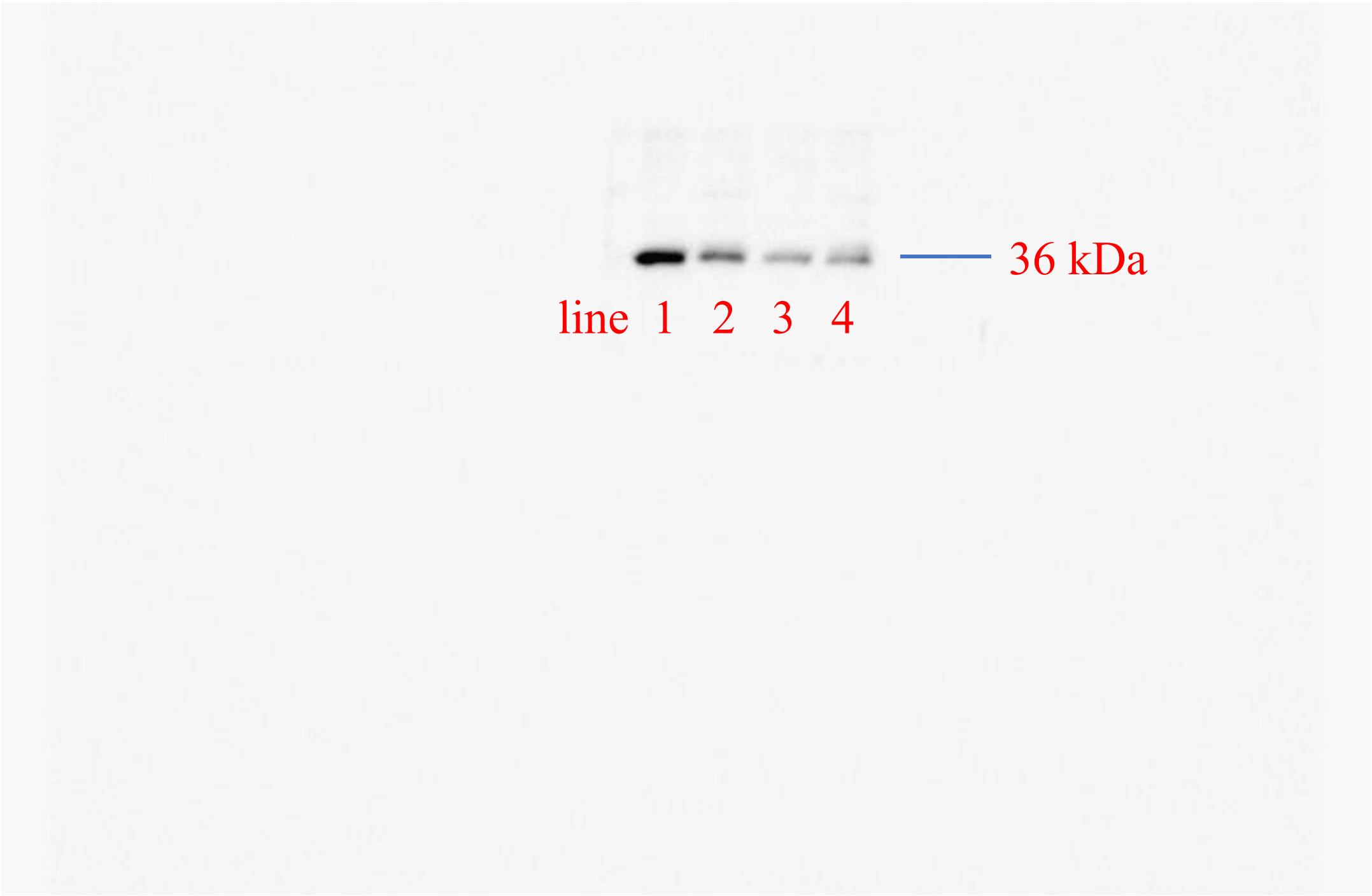

Raw unadjusted, uncropped blot for Figure 4b (5637)

Protein: Cyclin D1

Samples loaded order (left to right):

5637 PpD treatment: control (0), 62, 125, 250 nM (line 1-4)

Lanes removed in final figure: None.

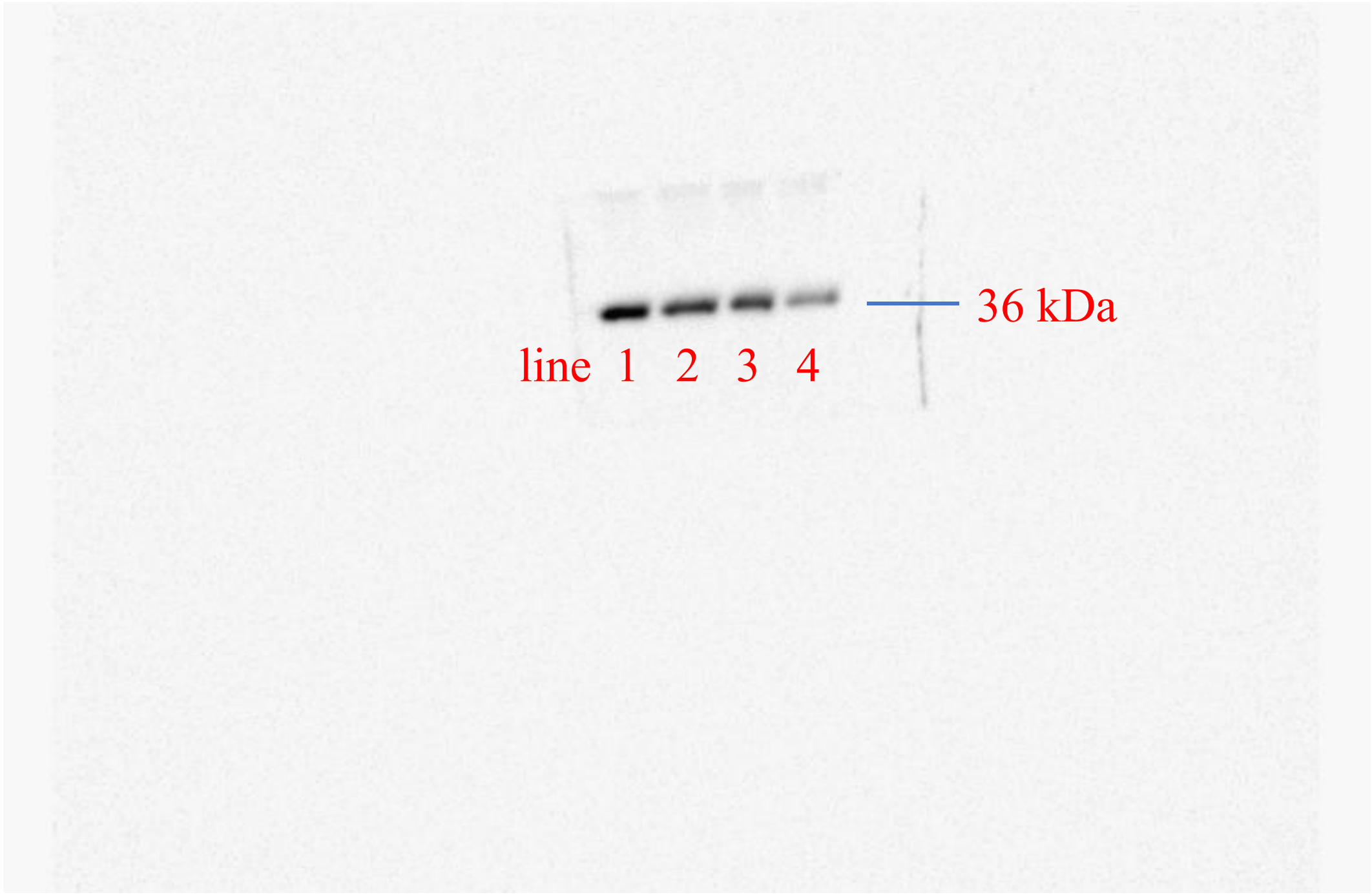

Raw unadjusted, uncropped blot for Figure 4b (T-24)

Protein: PCNA

Samples loaded order (left to right):

T-24 PpD treatment: control (0), 62, 125, 250 nM (line 1-4)

Lanes removed in final figure: None.

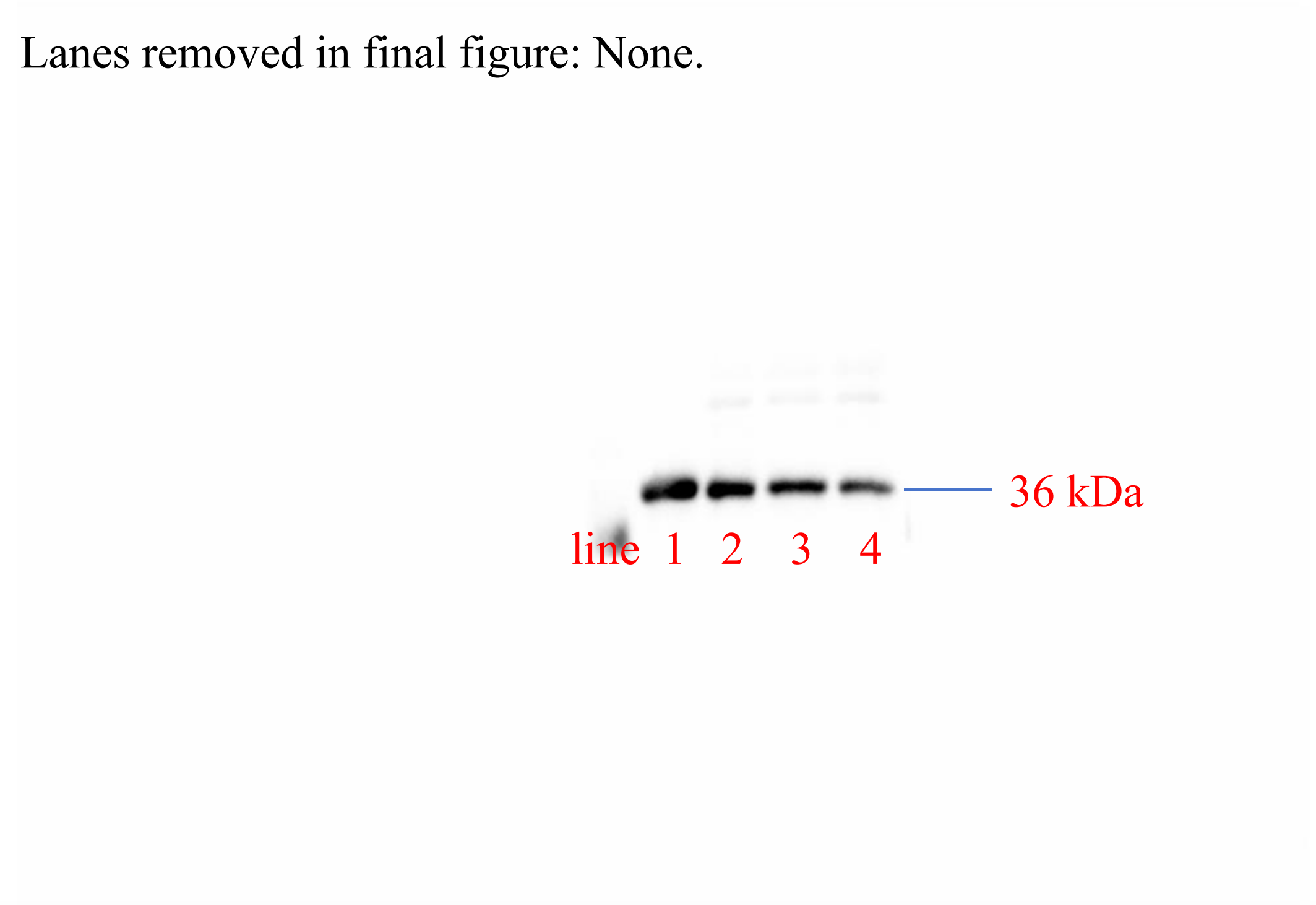

Raw unadjusted, uncropped blot for Figure 4b (5637)

Protein: PCNA

Samples loaded order (left to right):

5637 PpD treatment: control (0), 62, 125, 250 nM (line 1-4)

Lanes removed in final figure: None.

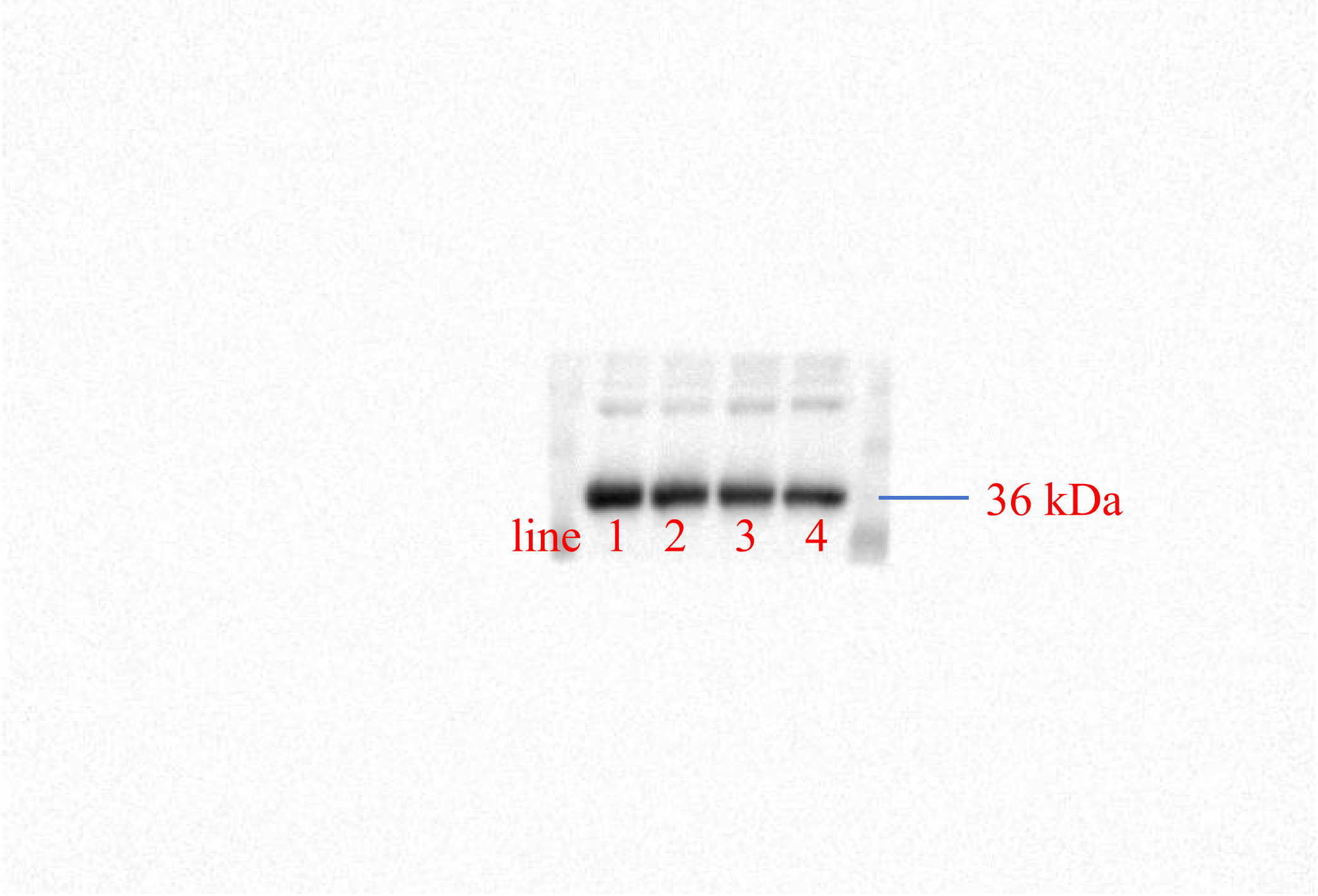

Raw unadjusted, uncropped blot for Figure 4b (T-24)

Protein: vinculin

Samples loaded order (left to right):

T-24 PpD treatment: control (0), 62, 125, 250 nM (line 1-4)

Lanes removed in final figure: None.

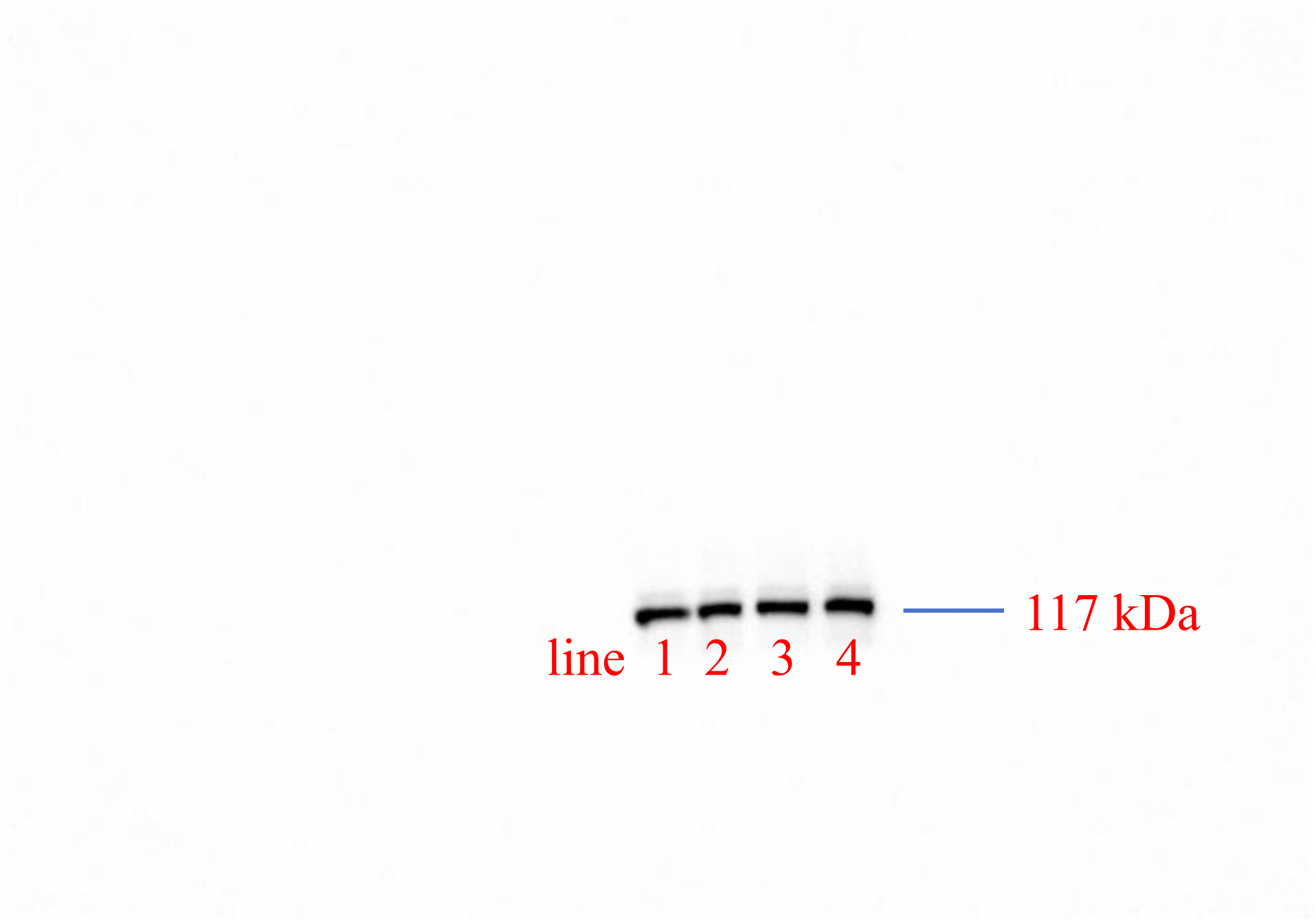

Raw unadjusted, uncropped blot for Figure 4b (5637)

Protein: vinculin

Samples loaded order (left to right):

5637 PpD treatment: control (0), 62, 125, 250 nM (line 1-4)

Lanes removed in final figure: None.

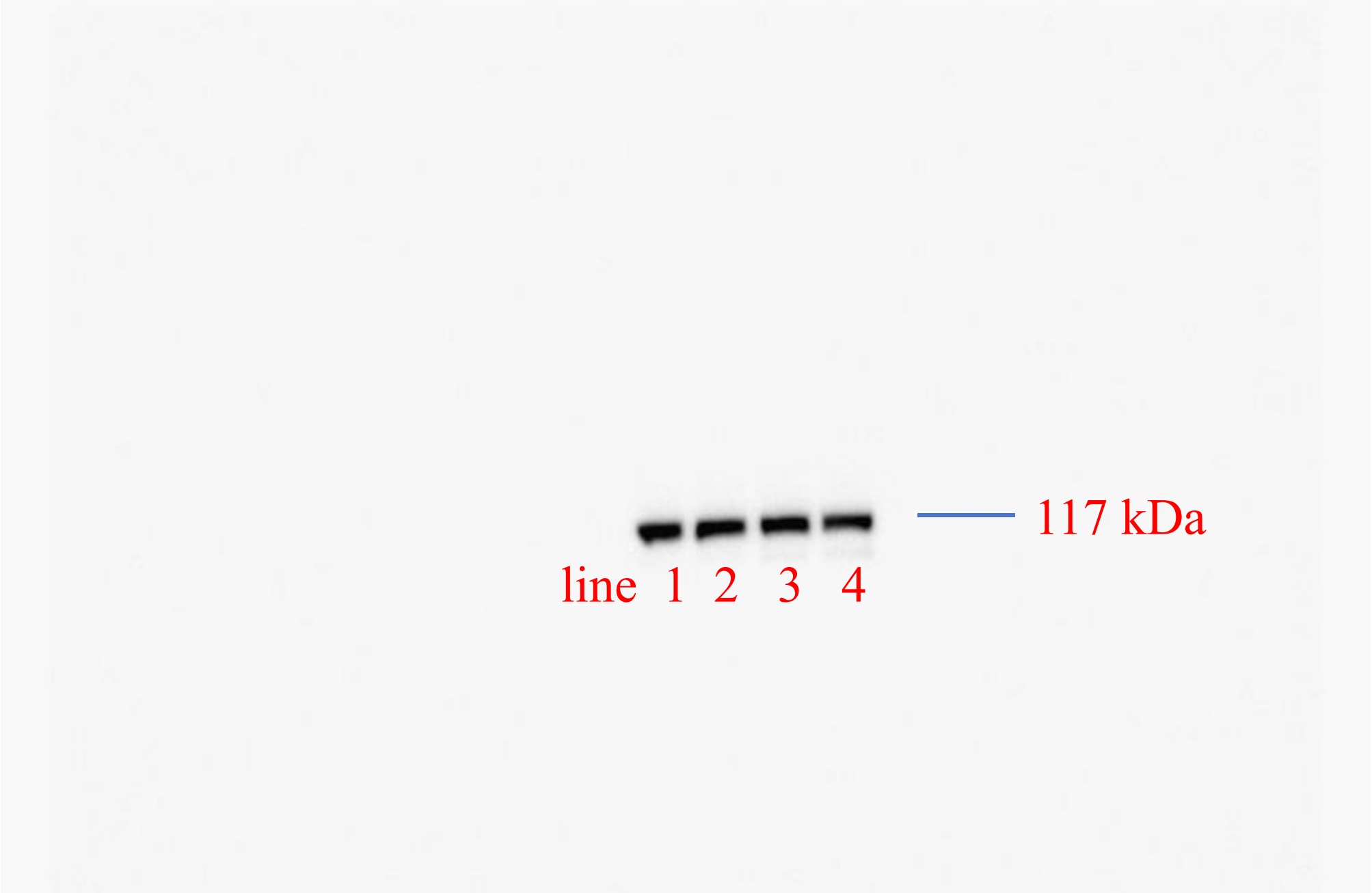

Raw unadjusted, uncropped blot for Figure 4c

Protein: CDK4

Samples loaded order (left to right): T-24 control, T-24 CDK4 OE, marker, 5637 control, 5637 CDK4 OE,

Lanes removed in final figure: Lane 3 (marked ×)

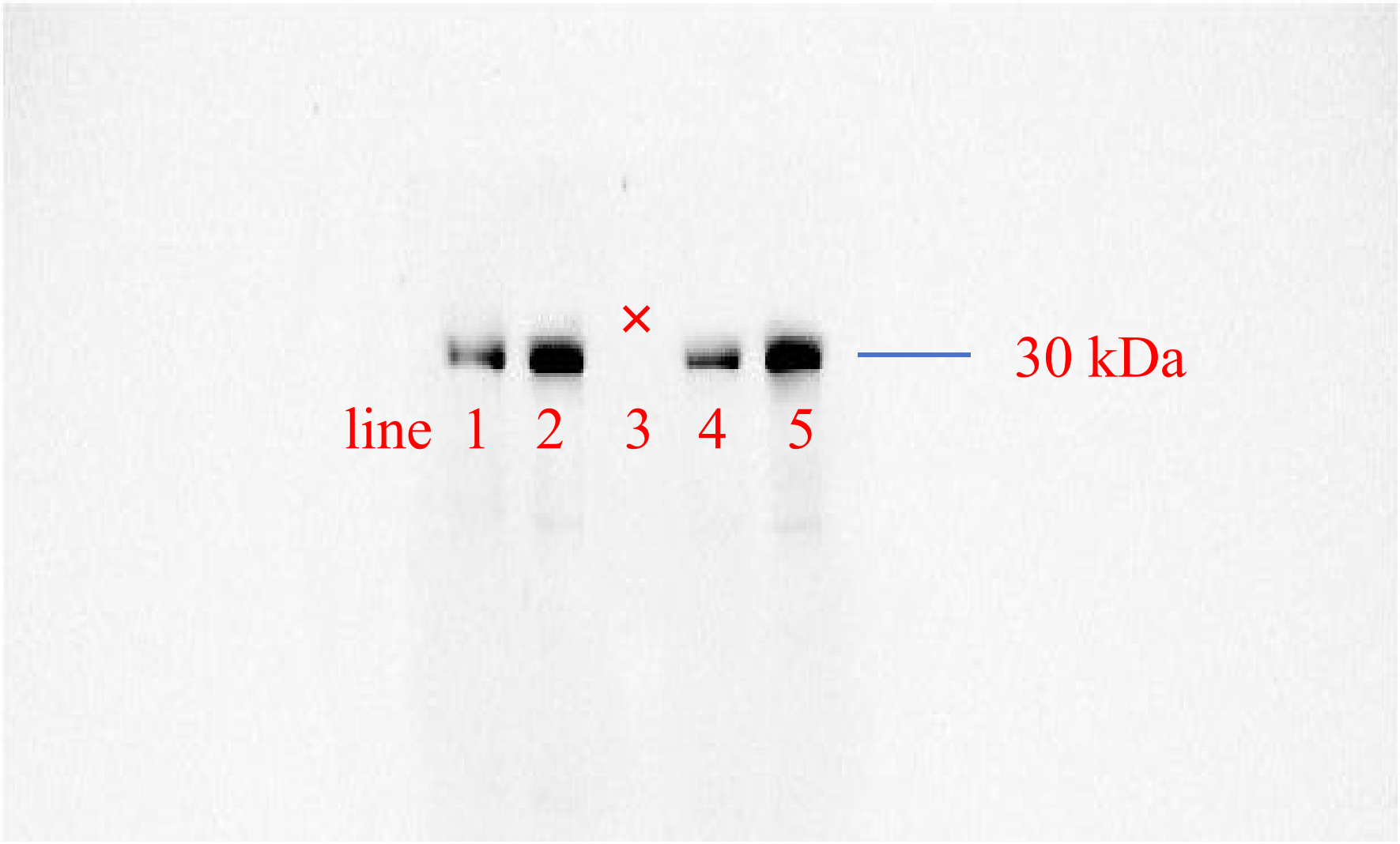

Raw unadjusted, uncropped blot for Figure 4c

Protein: vinculin

Samples loaded order (left to right): T-24 control, T-24 CDK4 OE, 5637 control, 5637 CDK4 OE,

Lanes removed in final figure: none

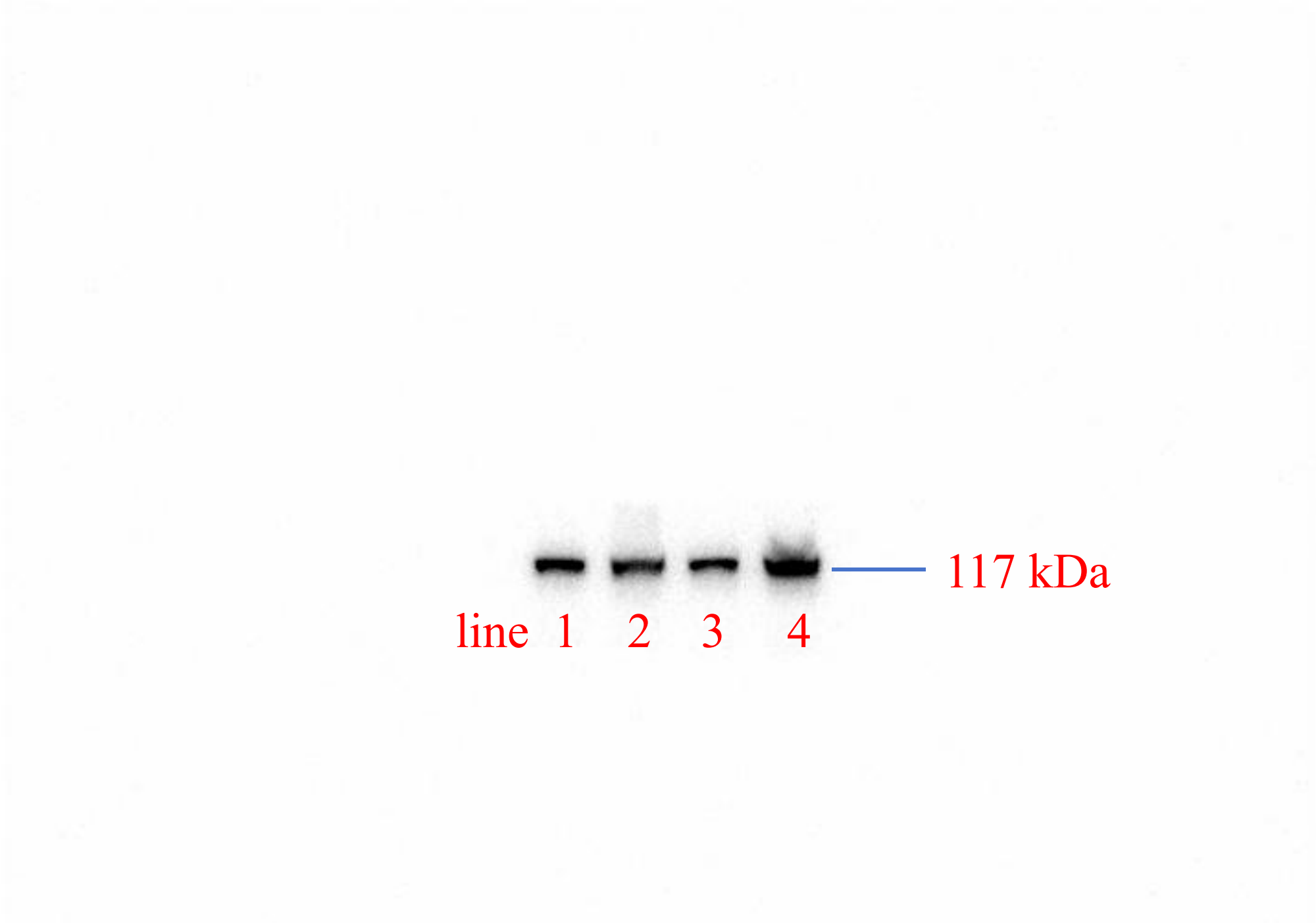

Raw unadjusted, uncropped blot for Figure 5e

Protein: PAK4

Samples loaded order (left to right): control, PpD, sg-PAK4-10

Lanes removed in final figure: None.

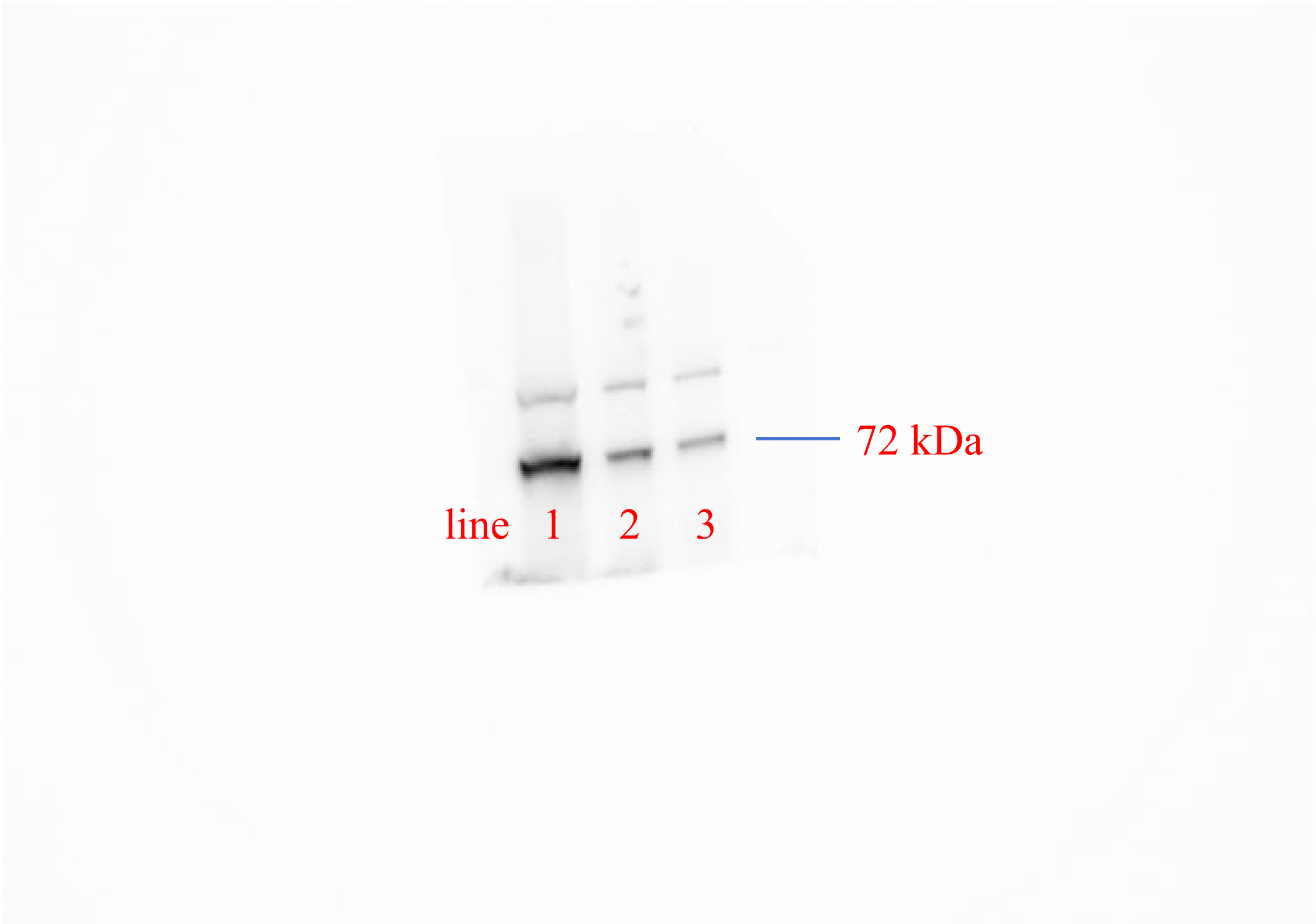

Raw unadjusted, uncropped blot for Figure 5e

Protein: PARP and cleaved PARP

Samples loaded order (left to right): control, PpD, sg-PAK4-10

Lanes removed in final figure: None.

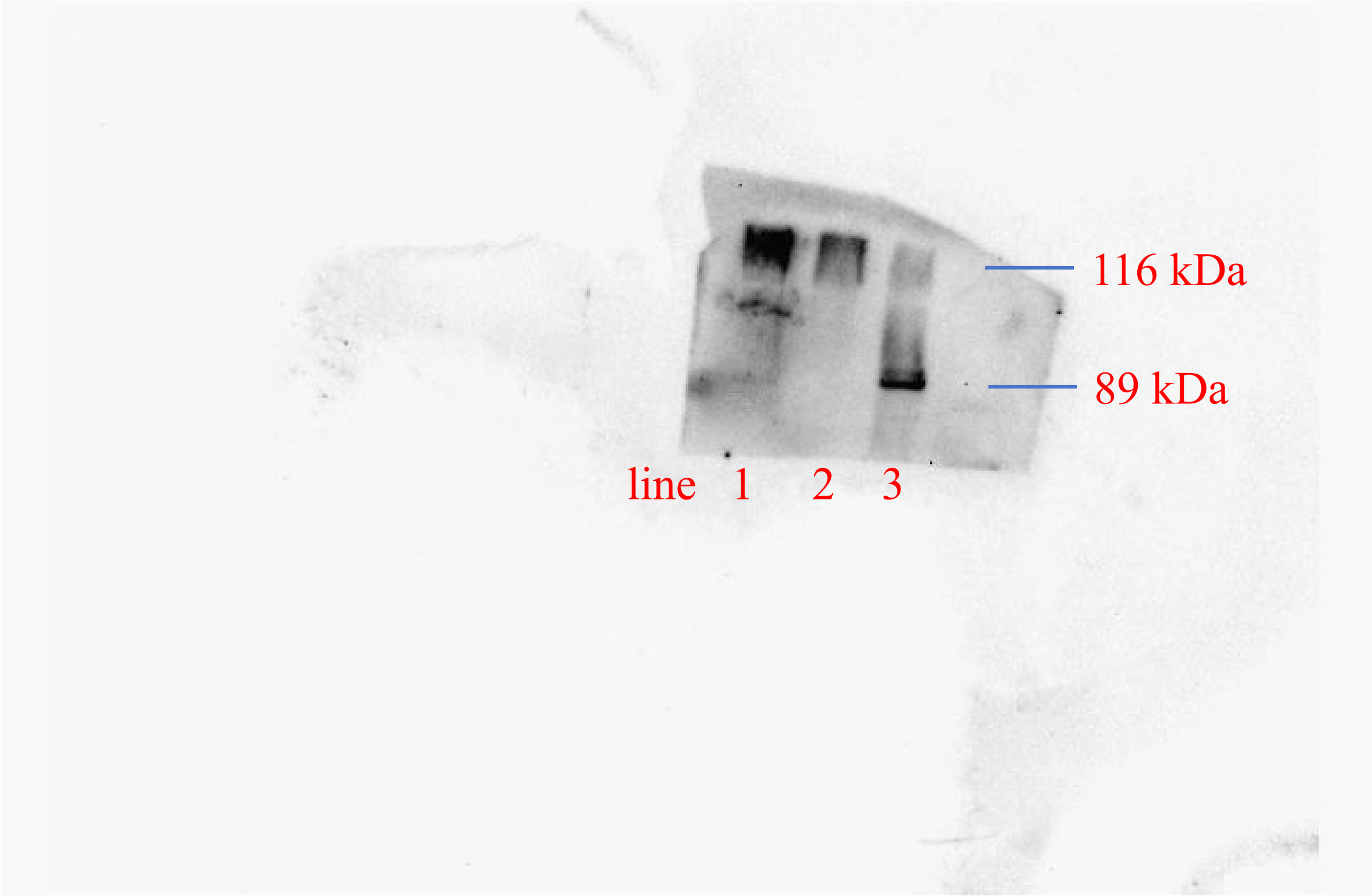

Raw unadjusted, uncropped blot for Figure 5e

Protein: Caspase 3

Samples loaded order (left to right): control, PpD, sg-PAK4-10

Lanes removed in final figure: None.

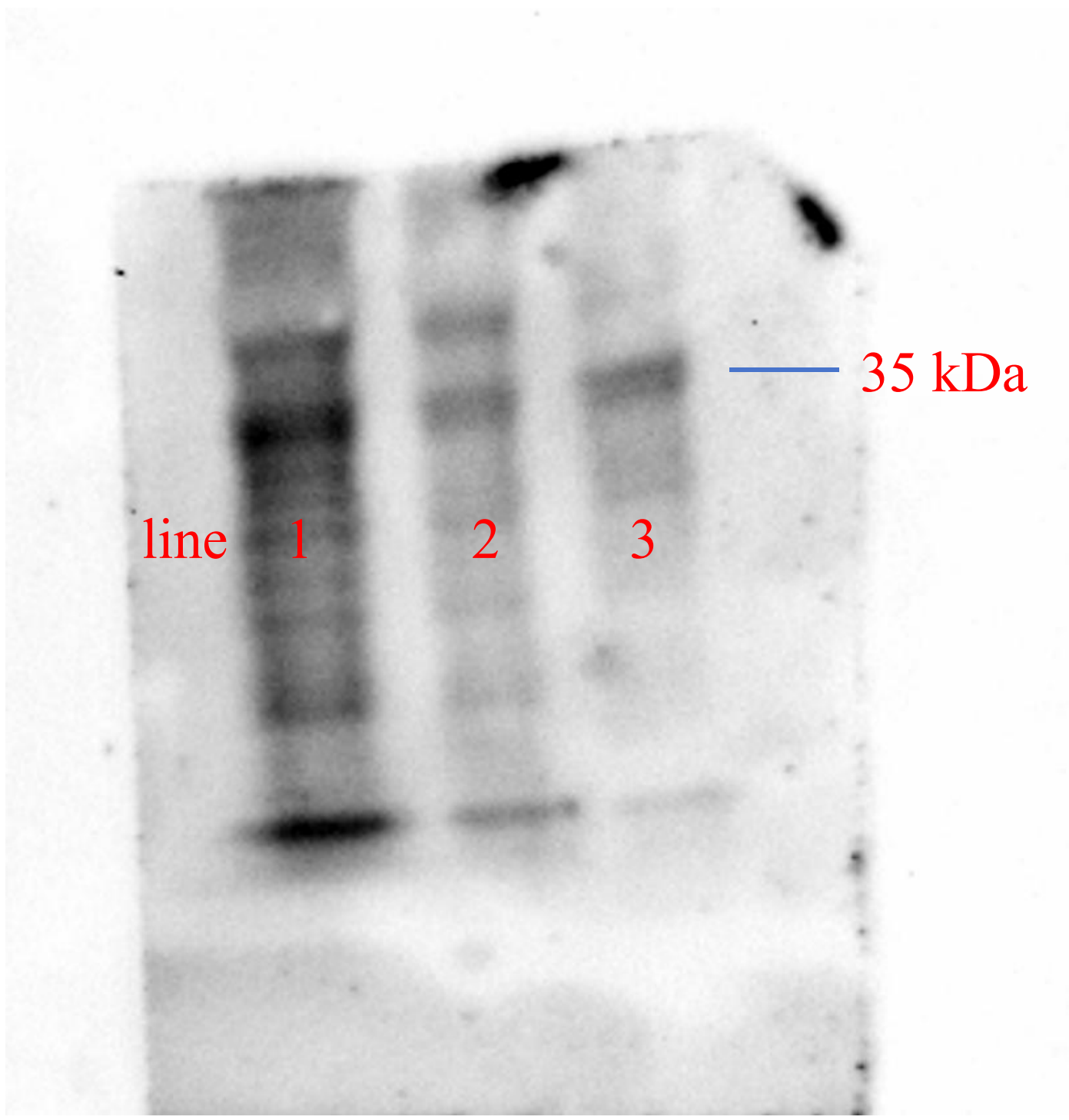

Raw unadjusted, uncropped blot for Figure 5e

Protein: ErK

Samples loaded order (left to right): control, PpD, sg-PAK4-10

Lanes removed in final figure: None.

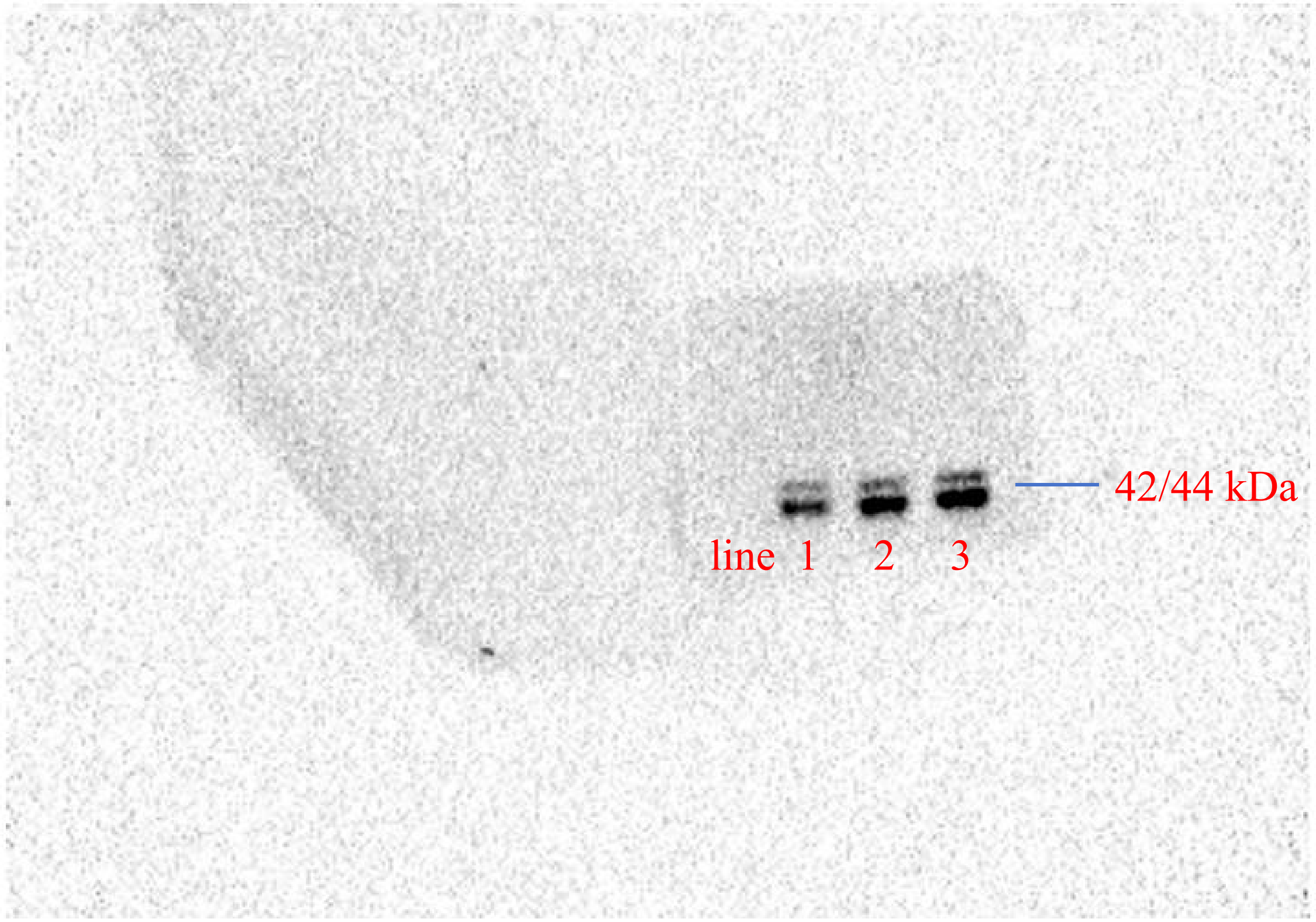

Raw unadjusted, uncropped blot for Figure 5e

Protein: p-ErK

Samples loaded order (left to right): control, PpD, sg-PAK4-10

Lanes removed in final figure: None.

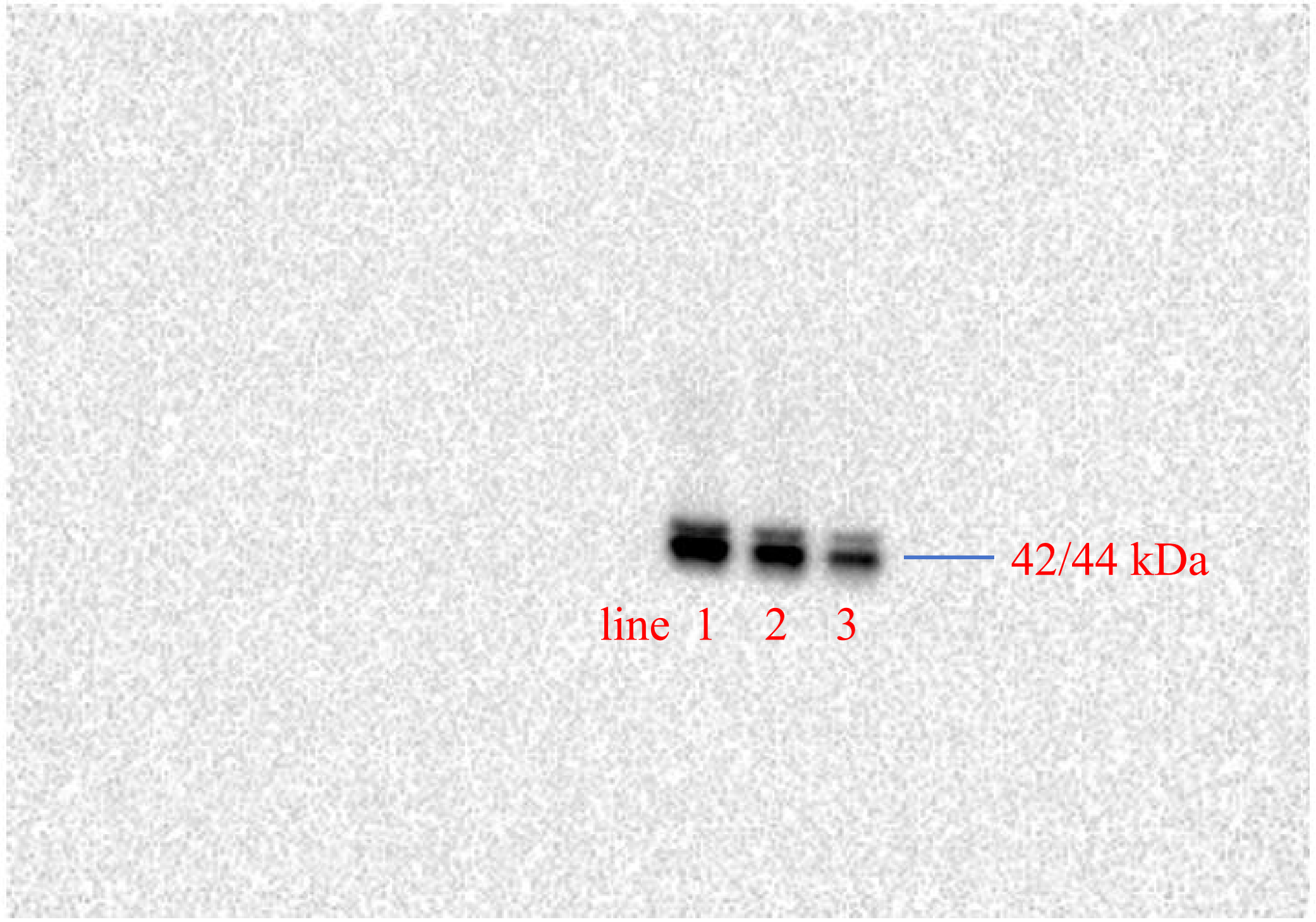

Raw unadjusted, uncropped blot for Figure 5e

Protein: CDK4

Samples loaded order (left to right): control, PpD, sg-PAK4-10

Lanes removed in final figure: None.

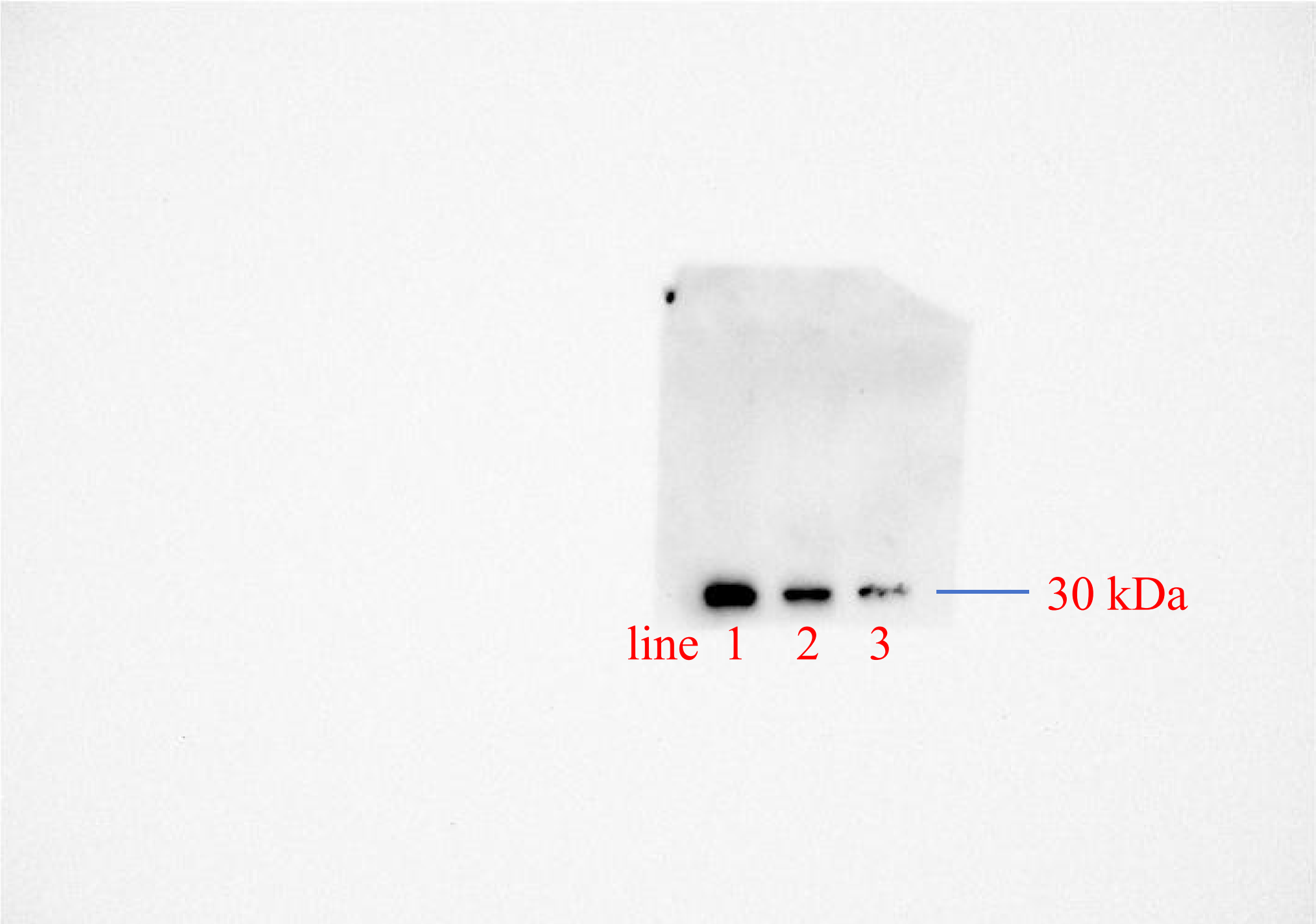

Raw unadjusted, uncropped blot for Figure 5e

Protein: Cyclin D1

Samples loaded order (left to right): control, PpD, sg-PAK4-10

Lanes removed in final figure: None.

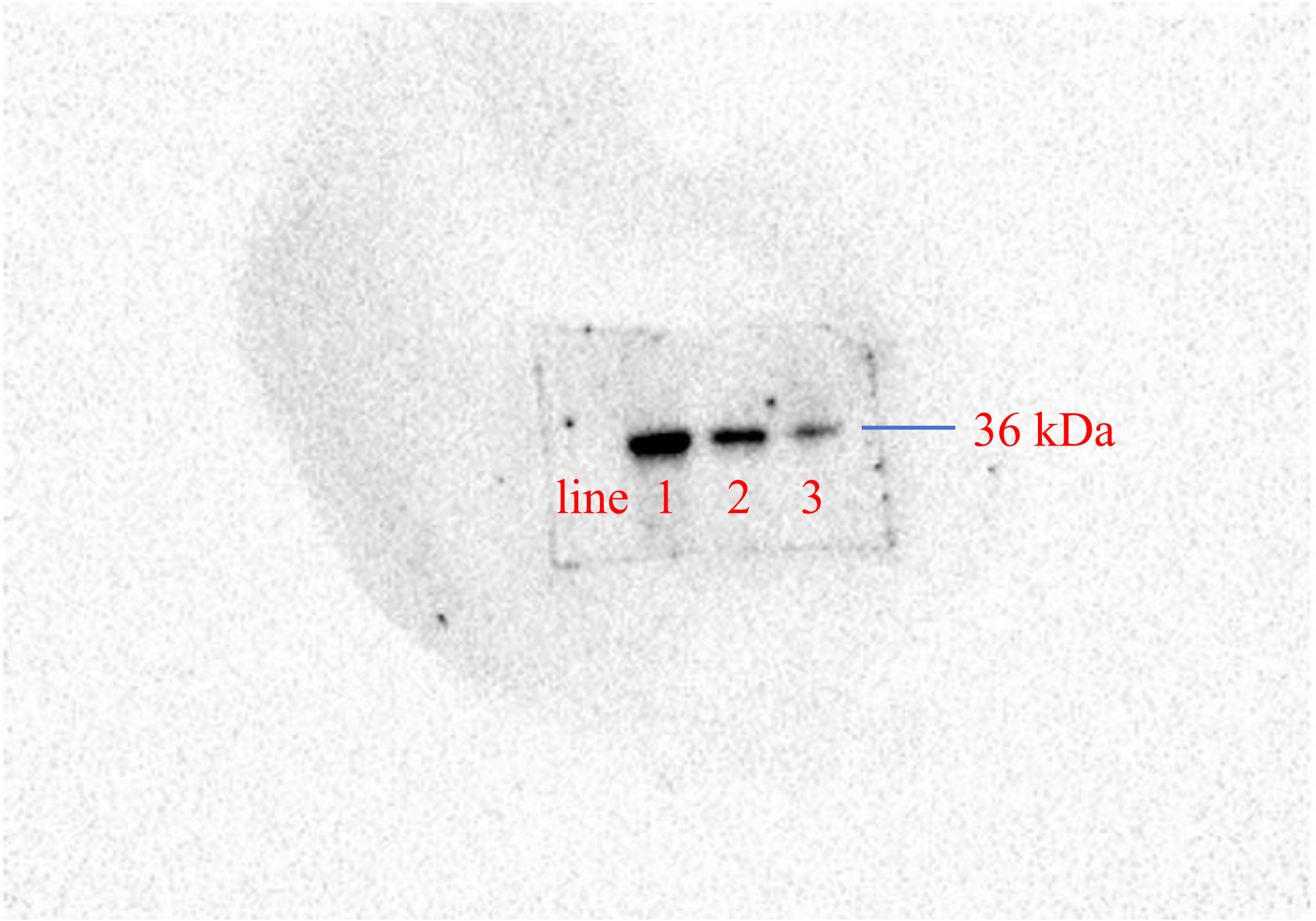

Raw unadjusted, uncropped blot for Figure 5e

Protein: PCNA

Samples loaded order (left to right): control, PpD, sg-PAK4-10, marker, marker

Lanes removed in final figure: Lane 4-5 (marked ×).

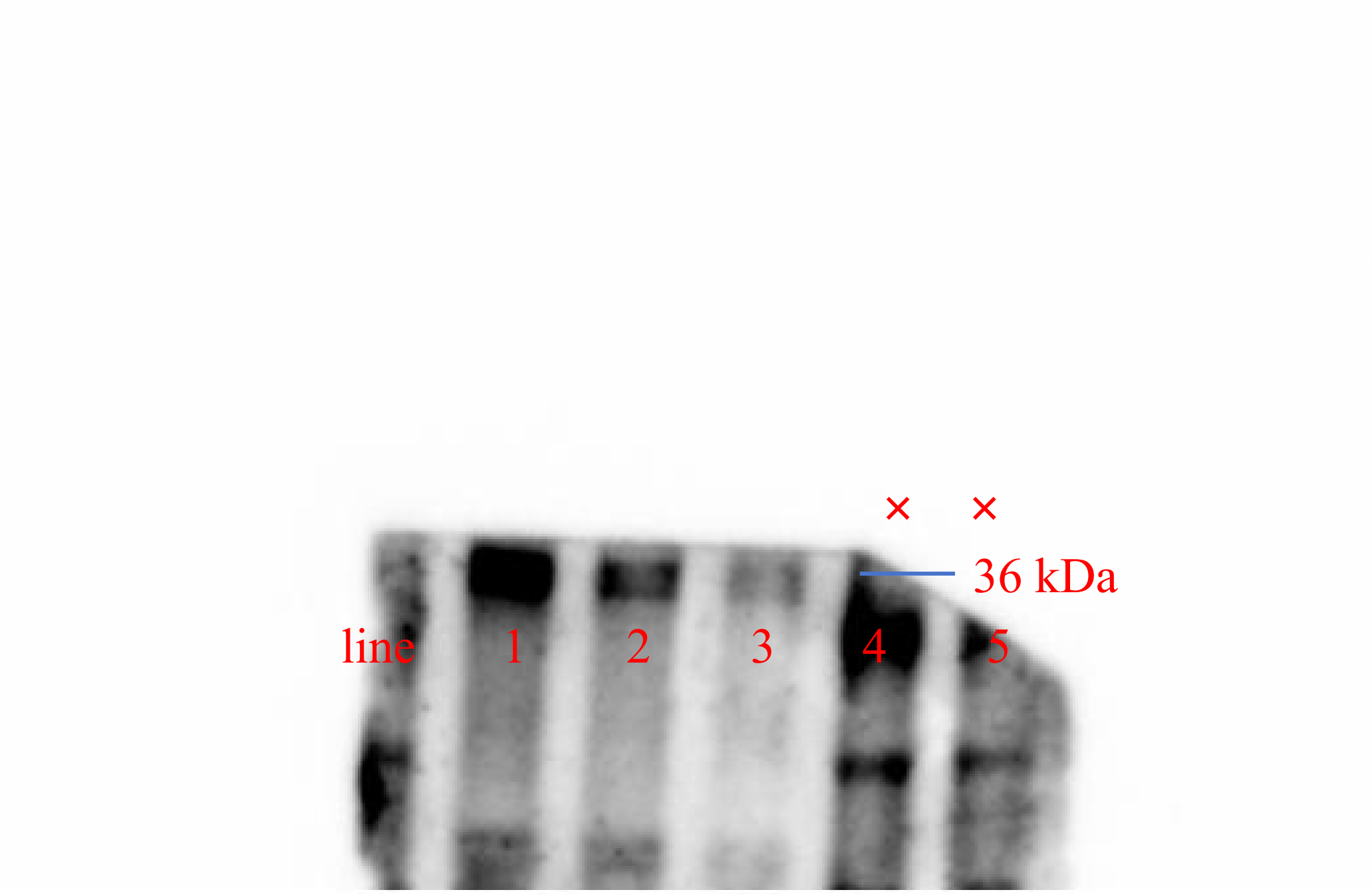

Raw unadjusted, uncropped blot for Figure 5e

Protein: vinculin

Samples loaded order (left to right): control, PpD, sg-PAK4-10

Lanes removed in final figure: None.

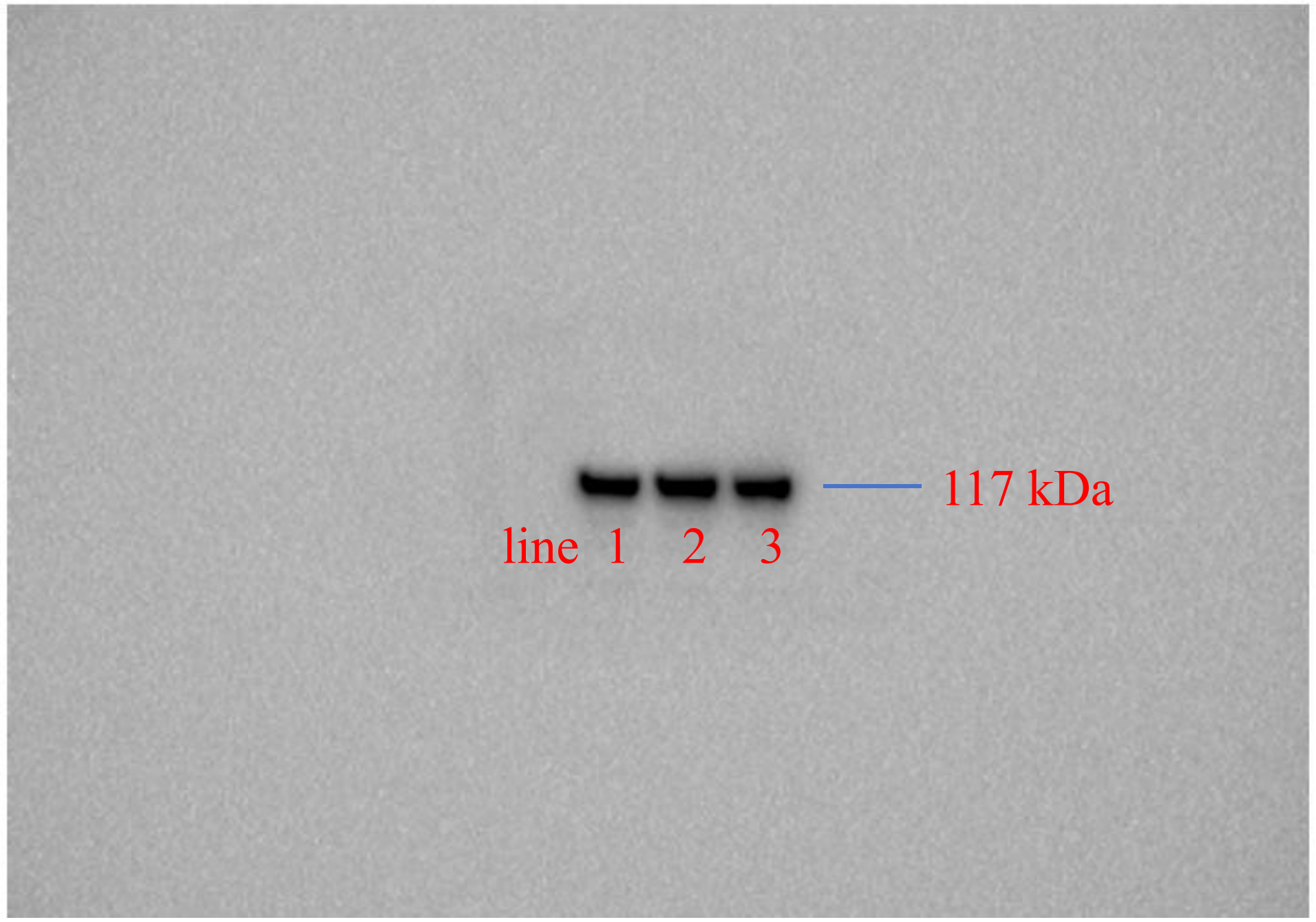

Supplement: Supplementary file 1 [file pone.0358561.s001.pdf]
