# Supplementary material for: PAK4-targeting PROTAC inhibits bladder cancer cell proliferation
Source: PLoS One. 2026 Sep 21;21(9):e0358561. doi: 10.1371/journal.pone.0358561 (PMC13592572; doi:10.1371/journal.pone.0358561)
Supplement: Supplementary file 2 [file pone.0358561.s002.docx]

**PAK4-Targeting PROTAC** **Inhibits Bladder Cancer Cell Proliferation**

**Shan Xu^1,2^*, Hongjun Xie^1,2^, Zhao Yang^1,2^, Zhenkun Ma^1,2^, Xiaojing Bai^1,2^, Xiaoyu Feng^1,2^, Jiale An^1,2^, Bohan Ma^1,2^*,**

^1^ Department of Urology, The First Affiliated Hospital of Xi’an Jiaotong University, ^#^277 Yanta West Road, Xi’an, 710061, China.

^2^ Key Laboratory for Tumor Precision Medicine of Shaanxi Province, The First Affiliated Hospital of Xi'an Jiaotong University, ^#^277 Yanta West Road, Xi'an, 710061, China.

*****Corresponding author: Bohan Ma, ^#^277 Yanta West Road, Shaanxi, China, 710061, [bohanma@xjtu.edu.cn](mailto:bohanma@xjtu.edu.cn);

Shan Xu, ^#^277 Yanta West Road, Shaanxi, China, 710061, shanhuxs1109@xjtu.edu.cn.

**S1 Fig.**

**S1 Fig.** (**a**) The identified differentially expressed genes were showed by volcano plot between control group and sg-PAK4-10 group. 1506 up-expression genes and 647 down-expression genes in treatment group compared with control group. FDR ≤ 0.05 and expression fold-change ≥ 2 were set as the cut-off criterion of significant difference. Red dots, significantly upregulated genes Yellow dots, significantly downregulated genes. Blue dots, nondifferentially expressed genes.

**S2 Fig.**

**S2 Fig.** (**a**) Time-dependent degradation profile of PpD in serum. The elimination half-life of PpD was calculated to be 10.97 h based on the residual drug percentage decay curve.
