# Supplementary material for: PAK4-targeting PROTAC inhibits bladder cancer cell proliferation
Source: PLoS One. 2026 Sep 21;21(9):e0358561. doi: 10.1371/journal.pone.0358561 (PMC13592572; doi:10.1371/journal.pone.0358561)
Supplement: Supplementary file 3 [file pone.0358561.s004.docx]

**Journal Requirements:**

1. When submitting your revision, we need you to address these additional requirements.

Please ensure that your manuscript meets PLOS ONE's style requirements, including those for file naming. The PLOS ONE style templates can be found at

https://journals.plos.org/plosone/s/file?id=wjVg/PLOSOne_formatting_sample_main_body.pdf and

<https://journals.plos.org/plosone/s/file?id=ba62/PLOSOne_formatting_sample_title_authors_affiliations.pdf>

Ans: Thank you for this reminder. We revised the manuscript formatting and file naming to meet PLOS ONE requirements.

2. To comply with PLOS One submissions requirements, in your Methods section, please provide additional information regarding the experiments involving animals and ensure you have included details on (a) methods of sacrifice, (b) methods of anesthesia and/or analgesia, and (c) efforts to alleviate suffering.

Ans: We thank our editor for raising these questions.We have added the following details to the Methods section to comply with PLOS ONE animal research requirements, in manuscript R2 as listed below.

Changes in the text:

---All mice were raised in the Animal Centre of Xi’an Jiaotong University Health Science Centre in an SPF environment. In whole study, euthanasia was performed by cervical dislocation by trained personnel, and no anesthesia was required for our study as procedures did not involve surgery or cause significant pain. Mice were humanely euthanized by spinal dislocation immediately when mice tumor’s side length close to 2 cm. In our study, mice were monitored daily for signs of distress, pain, or illness. If mice exhibited severe morbidity or unexpected weight loss (>20 % of baseline) will be removed from the study and euthanized spinal dislocation immediately. All handling and injections were performed by trained personnel using gentle restraint techniques to reduce stress.**(see page 9, line 180-189.)**

3. Thank you for stating in your Funding Statement:

This work was supported by the National Natural Science Foundation of China [82373103, Shan Xu] and Research Funds on Social Development from the Department of Science and Technology of Shaanxi Province of China (grant numbers 2024SF-YBXM-192, Xiaojing Bai).

Please provide an amended statement that declares *all* the funding or sources of support (whether external or internal to your organization) received during this study, as detailed online in our guide for authors at http://journals.plos.org/plosone/s/submit-now. Please also include the statement “There was no additional external funding received for this study.” in your updated Funding Statement.

Please include your amended Funding Statement within your cover letter. We will change the online submission form on your behalf.

Ans: Thank you for this reminder. We revised Funding Statement in R2 and cover letter. Changes in the text:

---There was no additional external funding received for this study.**(see page 25, line 528-529.)**

4. Thank you for stating the following financial disclosure:

This work was supported by the National Natural Science Foundation of China [82373103, Shan Xu] and Research Funds on Social Development from the Department of Science and Technology of Shaanxi Province of China (grant numbers 2024SF-YBXM-192, Xiaojing Bai).

Please state what role the funders took in the study. If the funders had no role, please state: "The funders had no role in study design, data collection and analysis, decision to publish, or preparation of the manuscript."

If this statement is not correct you must amend it as needed.

Please include this amended Role of Funder statement in your cover letter; we will change the online submission form on your behalf.

thics statement entered into the online submission form will not be published alongside your manuscript.

Ans: Thank you for this reminder. We have stated “The funders had no role in study design, data collection and analysis, decision to publish, or preparation of the manuscript.” in our cover letter.

7. Please include captions for your Supporting Information files at the end of your manuscript, and update any in-text citations to match accordingly. Please see our Supporting Information guidelines for more information: http://journals.plos.org/plosone/s/supporting-information.

Ans: Thank you for this reminder. We included our supporting Information files at the end of your manuscript, and cited in R2.

8. PLOS ONE now requires that authors provide the original uncropped and unadjusted images underlying all blot or gel results reported in a submission’s figures or Supporting Information files. This policy and the journal’s other requirements for blot/gel reporting and figure preparation are described in detail at https://journals.plos.org/plosone/s/figures#loc-blot-and-gel-reporting-requirements and https://journals.plos.org/plosone/s/figures#loc-preparing-figures-from-image-files. When you submit your revised manuscript, please ensure that your figures adhere fully to these guidelines and provide the original underlying images for all blot or gel data reported in your submission. See the following link for instructions on providing the original image data: https://journals.plos.org/plosone/s/figures#loc-original-images-for-blots-and-gels.

In your cover letter, please note whether your blot/gel image data are in Supporting Information or posted at a public data repository, provide the repository URL if relevant, and provide specific details as to which raw blot/gel images, if any, are not available. Email us at plosone@plos.org if you have any questions.

Ans: Thank you for reminder. We uploaded the original uncropped and unadjusted images underlying all blot or gel results reported in a submission’s figures or Supporting Information files.

9. If the reviewer comments include a recommendation to cite specific previously published works, please review and evaluate these publications to determine whether they are relevant and should be cited. There is no requirement to cite these works unless the editor has indicated otherwise.

Ans: Thank you for reminder.

**Reviewer #1:** The study presents a promising investigation into a novel PROTAC-based therapeutic strategy for bladder cancer (BCa). The research question is relevant, and the experimental design is generally logical, progressing from target validation to in vivo proof-of-concept. The manuscript is clearly written. However, several major concerns must be addressed to strengthen the scientific rigor, mechanistic insight, and overall impact of the work before it can be considered for publication. My primary recommendations are outlined below.

Ans: Thank you for your positive comment.

1. In Figure 1c and 1d, the authors highlight Hazard Ratios (HR=1.6 and 1.2) as "statistically significant" for linking high PAK4 expression to poor survival. However, the presented Kaplan-Meier curves and associated log-rank p-values do not appear to reach statistical significance. The claim of a direct link to poor prognosis cannot stand without statistically robust support.

Ans: We appreciate the reviewer's concern regarding the statistical robustness of our prognosis association. We changed the conclusion and inaccurate description in our manuscript R1.

Changes in the text:

---PAK4 was highly expressed in Bca and predicted poor prognosis.**(see page 10, line 209-210.)**

---The HR values (1.6 and 1.2, respectively). **(see page 11, line 222.)**

2. To establish causality, key experiments are needed. For example, does constitutive activation of MEK/ERK or overexpression of Cyclin D1/CDK4 rescue the cell cycle arrest and growth inhibition caused by PpD treatment or PAK4 knockout?

Ans: Thank you very much for reminding this point to us. To establish causality, we have performed the requested rescue experiments. CDK4 were co-overexpressed in T-24 and 5637 cells prior to PpD treatment. Fig 4d demonstrates that forced expression of CDK4 attenuated PpD-induced growth inhibition.

Changes in the text:

---To establish CDK4-overexpressing BLCA cell models, T-24 and 5637 cells were transfected with HA-tagged human CDK4 plasmids (MiaoLing, Hubei, China) using Lipofectamine 2000 (#11668-019, Invitrogen, USA) according to the manufacturer's protocol. **(see page 6, line 121--124.)**

---To further verify whether cyclin D1-CDK4 affects the effect of PpD on cell apoptosis, we overexpressed CDK4 in T-24 and 5637 cells (Fig 4c). Colony formation assay showed that CDK4-overexpressing cells proliferated significantly faster than control cells both in T-24 and 5637 cells (Fig 4d). Importantly, Ppd treatment inhibited cell proliferation in both control and CDK4-overexpressing cells. **(see page 18, line 381--385.)**

---(c) Cell lysates from T-24 cells, 5637 cells, and the CDK4 overexpression cells were analyzed by immunoblotting. The expression levels of cleaved CDK 4 and Vinculin were assessed. (d) Colony formation images of T-24 and 5637 cells overexpressing CDK4 or treated with PpD. Cells were seeded in 6-well dishes, cultured for 7 days, and stained with crystal violet, respectively. The cells were seeded in 6-well dishes, cultured for 7 days and stained with crystal violet. **(see page 19, line 392--397.)**

3. The study lacks a comparator group treated with a standard-of-care agent (e.g., cisplatin) or a known PAK4 inhibitor. This makes it impossible to assess the relative efficacy of PpD.

Ans: We appreciate the reviewer for raising these questions. We have addressed this concern in our PpD previous work. We conducted comparative experiments using a commercially available PAK4 inhibitor (PKT-9274 and PF-3758309). In our animal models (Subcutaneous tumor model, PDO, and mini-PDX), our developed compound demonstrated superior efficacy compared to the inhibitors at the same concentration. At the cellular level, our PpD exhibited markedly enhanced degradative activity against PAK4. These findings are detailed in our prior publication **[reference 27].**

The previously results (Response figure1 and 2, given below):

Response figure1: PpD has a highly selective PAK4 protein degradation than PAK4 inhibitor. (A) Western blotting analysis of PAK1, PAK2, PAK3, and PAK4 in WCLs from 786-O, ACHN, and Caki-1 cells after treatment with 0, 100, and 200 μM of KPT-9274 for 48 hours. (B) Specificity of PpD towards PAK4 in RCC cell lines. After treatment with 0, 250, or 500 nM PpD for 48 h, western blotting analyses of PAK1, PAK2, PAK3, and PAK4 in whole cell lysates (WCLs) from 786-O and Caki-1 cells were performed.

Response figure 2: PpD demonstrates potency in renal cancer Subcutaneous tumor model, mini patient-derived xenograft (PDX) and patient-derived organoid (PDO) models.

(A) Photos of ACHN tumors excised at the end of the experiment after different drug. Tumor-bearing BALB/c nude mice treated with indicated drugs. 20 BALB/c nude mice were randomly separated into 4 groups and treated with Control, PBS, n=5, Se (Se-mutation peptide nano particles, n = 5), PpD (2.5 mg/kg, n=5), or PF-3758309 ( 2.5 mg/kg, n=5) every 3 days. The tumor volume and mice weight of BALB/c nude mice were measured and recorded every 3 days until sacrifice. (B) Representative pictures of clear cell ccRCC PDO cells after treated with 1 μM of PpD, 1 μM of KPT-9274, and 1 μM of PF-3758309 for 48 h, respectively. (C) Mini PDX assay showing the anti-tumor effect of PpD, KPT-9274, and PF-3758309 2.5 mg/kg *in vivo.*
4. Only a single dose of PpD (2.5 mg/kg) was tested. A dose-ranging study is needed to determine the optimal effective dose and potential toxicity.

Ans: We appreciate the reviewer for raising these questions. In our previous studies, we indeed tested various drug concentrations and found that high-dose PpD (5.0 mg/kg, the red rectangle ) did not exhibit concentration-dependent inhibitory effects in vivo; therefore, we selected the dose of 2.5 mg/kg for subsequent experiments.

The previously results (Response figure3, given below):

Response figure 3: 5.0 mg/kg PpD did not confer additional inhibition in the renal cancer subcutaneous tumor model. (A, B) Photos of 786-O tumors excised at the end of the experiment after different drug. Tumor-bearing BALB/c nude mice treated with indicated drugs. 25 BALB/c nude mice were randomly separated into 5 groups and treated with Control, Se (Se-mutation peptide nano particles, n = 5), KPT-9274 ( 2.5 mg/kg, n=5), PpD (2.5 mg/kg, n=5), or PpD (5.0 mg/kg, n=5) every 3 days.

Changes in the text:

---Based on our previous results, increasing the PpD dose to 5.0 mg/kg failed to enhance the antitumor effect in the renal cancer subcutaneous tumor model, indicating that 2.5 mg/kg represents the optimal dosage for maximal therapeutic benefit. Therefore, we selected the indicated dose of 2.5 mg/kg for the PpD group (2×10⁶ T-24 cells, n = 5). **(see page 19, line 403--407.)**

5. Please provide immunohistochemistry or western blot data from excised tumors to show PAK4 degradation, reduced p-ERK/CDK4, and increased apoptosis (e.g., cleaved caspase-3 staining) in the PpD-treated group compared to the control.

Ans: We are immensely grateful for the reviewers’ comments. We have performed the requested analyses on excised tumor tissues from our xenograft study.

Changes in the text:

---In Fig. 5f, tumor tissues were collected from the xenograft experiment. WB analysis of tissue lysates from the control, PpD-treated, and T-24 sg-PAK4-10 groups showed the expression levels of cleaved PARP, PAK4, PARP, cleaved Caspase-3, Cyclin D1, CDK4, p-ERK1/2, total ERK1/2, PCNA, and Vinculin. Compared to the control group, both PpD treatment and PAK4 knockout reduced PAK4 protein levels, decreased ERK1/2 phosphorylation, and downregulated CDK4 expression. Collectively, our findings indicate that PpD effectively suppresses the in vivo growth of BLCA cells (Fig 5g). **(see page 20, line 421--428.)**

--- (f) WB showed the expression levels of cleaved PARP, PAK4, PARP, Caspase 3, Cyclin D1, CDK 4, Erk1/2, PCNA, and Vinculin. Tissue lysates from the control, PpD-treated, and T-24 sg-PAK4-10 groups, respectively. (g) PpD, a highly potent and selective PAK4 degrader, targets and degrades the PAK4 protein in BLCA cells. Treatment with the PpD drug inhibits the ERK1/2-CDK4 signaling pathway, leading to an impediment in the cell cycle transition from the G1 phase to the S phase. Furthermore, it induces tumor cell apoptosis, thereby suppressing the proliferation and growth of BLCA tumor cells. **(see page 21, line 449-456.)**

6. The abbreviation for bladder cancer is inconsistently used (“BLCA” in figures and database contexts, “Bca” in the text). Please choose one (e.g., BCa) and use it consistently throughout the manuscript for clarity.

Ans: We appreciate your reminding and apologize for two abbreviation for bladder cancer. In our manuscript R1, we have changed “Bca” to “BLCA”, the text all in red.

7. More critically discuss the potential advantages and challenges of the PROTAC approach compared to traditional PAK4 kinase inhibitors.

Ans: We extend our sincere gratitude to the reviewer for their suggestion, and we have meticulously re-written the discussion part.

Changes in the text:

---Here, we should mentioned that PAK4 inhibitors rely on binding to the ATP-binding site or specific conformations to block enzymatic activity; however, this approach merely suppresses PAK4 protein function without eliminating its physical presence[24, 41, 42]. In contrast, PpD can theoretically "eradicate" PAK4 from cells, thereby abrogating all pathogenic functions, including both enzymatic and non-enzymatic activities. The "clearance" mechanism of PROTACs enables the elimination of low-abundance or "undruggable" proteins (such as mutants or specific domains), which is typically challenging to achieve with conventional inhibitors[43, 44]. However, although PROTACs exhibit high degradation efficiency in vivo, achieving an appropriate pharmacokinetic balance remains a substantial challenge. The crux lies in reconciling the potent degradation capacity of PROTACs with favorable pharmacokinetic properties, while ensuring precise activity in specific tissues[45].**(see page 24, line 505--517.)**

**Reviewer #2:** The pivotal role of P21-activated kinase (PAK) in tumorigenesis and progression has garnered increasing attention. Among these, PAK4 mediates multiple biological behaviors at the cellular level, including tumor initiation, proliferation, metastasis, drug resistance, and immune evasion. PAK4 serves as a predictive, effective, and broadly applicable biomarker and therapeutic target for tumors. Several PAK4 inhibitors have demonstrated therapeutic efficacy in animal studies.

The paper focuses on PpD, a peptide-protein hydrolysis-targeted chimeric drug independently developed by the authors' team. Through cellular and animal models, it validates PpD's role in degrading PAK4 and its therapeutic efficacy in bladder tumors.

The paper presents a logically closed framework with detailed and appropriate methodology, employing validation across multiple levels and perspectives to yield credible conclusions. It may offer new therapeutic options for bladder tumors in the future.

The paper is now in a highly mature state. The following is intended solely for discussion among peers and does not affect the overall evaluation of the full paper.

Ans: We thank the reviewer for the kind comments.

1. Figure 1a indicates that PAK4 mRNA expression levels are higher in many other tumor tissues than in BLCA. The study currently employs only T-24 and 5637 cells as cellular models. Therefore, would it be more comprehensive to add renal carcinoma cells as positive controls and smooth muscle cells as negative controls, as suggested in the introduction?

If further experimental conditions are constrained or effective working time is limited, does PpD's effect on PAK4 degradation observed in Figure 1a also hold true in tumor cells beyond bladder tumors? What is the current status of research progress?

In other words, what is the distribution pattern of PAK4 in normal tissues? If PpD were to be applied clinically, what potential effects might it have on these numerous normal tissues containing the target? What is the authors' perspective on this?

Ans: We thank the reviewer for the insightful suggestions. We have addressed this concern in our PpD previous work. We conducted comparative experiments using HK-2 cells (proximal tubule epithelial cells), and renal cancer cells. At the cellular level, PpD selectively targets tumor cells, with malignant cells demonstrating significantly lower IC_50_ values relative to proximal tubule epithelial cells (**Response figure 4**). These findings are detailed in our prior publication **[reference 27]**.

We thank the reviewer for this insightful comment. As you mentioned: does PpD's effect on PAK4 degradation observed in Figure 1a also hold true in tumor cells beyond bladder tumors? What is the current status of research progress? Our unpublished data indicated that PpD also promotes PAK4 degradation in pancreatic, lung, and breast tumor cell lines. PpD was developed by our group and reported in ***eBioMedicine*** in 2024 **[reference 27]**. We are currently undertaking efforts to validate its biological functions across tumor types, as exemplified by the present investigation in bladder cancer.

We appreciate the reviewer for raising this important safety consideration regarding potential on-target toxicities in normal tissues. In this research, in Fig 1a, the PAK4 expression was evaluated in tumors relative to adjacent non-malignant tissues, PAK4 expression was higher in tumor tissues. We wish to clarify that our observation of heightened PpD sensitivity in tumor cells—consistent with our prior findings in renal cell carcinoma (**Response figure 4**). Our data indicating selective PpD sensitivity in tumor cells suggest a favorable therapeutic window; however, we agree that functional consequences of PAK4 degradation in non-malignant tissues require thorough preclinical evaluation.

The previously results (Response figure 4, given below):

Response figure 4: Tumor cells exhibit heightened sensitivity to PpD. (A, B, C) Western blotting analysis of PAK4 in WCLs from HK-2, ACHN, 786-O and Caki-1 cells after treatment with indicated concentration of PpD.

2. Figure 2f，T24 sg-control group: 4.22% + 5.12% + 90.03% + 0.45% = 99.82%，

Figure 3g，5637 62 nM grou: 3.74% + 7.05% + 0.94% + 88.5% = 100.23%，

If this is not a clerical error, what could cause such discrepancies? Does this indicate abnormal cell status within this batch during experimentation? What is the author's perspective on this?

Ans: We thank the reviewer for their careful examination of our data. Fig 2.f, T24 sg-control group: The correct values should be 4.22% + 5.33% + 90.21% + 0.45% = 100% (the original 90.03% was a typographical error in the figure preparation and has been corrected to 90.21%). Figure 3g, 5637 62 nM group: The correct values should be 3.47% + 7.05% + 0.94% + 88.54% = 100.00% (the original 3.74% was a typographical error in the figure preparation and has been corrected to 3.47%). The raw flow cytometry data and original gating strategies remain unchanged and are available upon request.

3. Line 269: “this effect was concentration dependent.”

However, as shown in Figure 3f, the proportion of cells in the G1 phase peaked at 125 nM PpD for both cell types, particularly in the 5637 cells.

Ans: We thank the reviewer for the insightful suggestions. We have re-examined the cell cycle data (Fig 3f) and revised the inaccurate descriptions accordingly. Based on the cell cycle assay results (Fig 3f), the effect of PpD on cell apoptosis was concentration-dependent, while no concentration-dependent G1 arrest was detected in the cell cycle. We have revised our description about the results in revised manuscript.

Changes in the text:

---To achieve this goal, we employed flow cytometry to assess the impact of PpD on the cell cycle in BLCA cells. As shown in Fig 3f, PpD increased the proportion of BLCA cells in the G1 phase of the cell cycle. Meanwhile, we found a small fraction of cells undergoing apoptosis in cell cycle assay. In further to test the PpD’s effect on BLCA cells popotosis, we designed cell apopotosis assay, under PpD drug treatment at 250 nM, T-24 cells presented a significant increase in the proportion of apoptosis cells, from 4.26% to 37.53%. Similar phenomena of apoptosis (from 10.0% to 42.08%) were observed in 5637 cells treated with PpD (Fig 3f). T24 and 5637 cell apoptosis significantly increased in a PpD concentration-dependent manner (Fig 3g). This shift in the cell cycle distribution and cell apoptosis underscores the effects of PpD on cell growth and death mechanisms. **(see page 16, line 329--340.)**

4. Line 306: “However, we did not detect any changes in caspase-3 or the cleavage of the PARP protein in the T-24 and 5637 cell lines in which PAK4 was knocked out.”

Since caspase-3 and PARP protein cleavage were already detected in the PAK4-knockout T-24 and 5637 cells, why are these results not presented in Figure 4a? Additionally, the vertical axes of Figure 4a and 4b should ideally be uniformly arranged and correspond to each other to facilitate comparison between figures and alignment with the corresponding results section in the paper.

Ans: We thank the reviewer for the important questions. We re-evaluated the apoptotic marker expression in PAK4-knockout cell lines. The observed discrepancy may be attributed to phenotypic drift during prolonged passaging (>10 passages). To address this, we generated PAK4-knockout T-24 and 5637 cells again, and harvested protein lysates at early passage (P1-P2). Western blot analysis of these early-passage knockout cells confirmed that caspase-3 is decreased and PARP cleavage is increased compared to controls, consistent with PpD treatment group.

Changes in the text:

---A similar decreasing trend was observed for caspase-3 and PARP, where the level of cleaved PARP protein was significantly greater following PpD treatment than in the controls (Fig 4b). Meanwhile, we also confirmed that caspase-3 and PARP protein are all decreased, and cleaved PARP protein is increased in the T-24 and 5637 cell lines in which PAK4 was knocked out (Fig 4a). **(see page 18, line 376--385.)**

---The expression levels of cleaved PARP, PAK4, PARP, Caspase 3, Cyclin D1, CDK 4, Erk1/2, PCNA, and Vinculin were assessed. **(see page 18, line 388--389.)**

5. Line 317 lists n=10, whereas Figure 5a shows n=5. This may be a typographical error, please verify.

Ans: We apologize for this error. We have corrected the error in the revised manuscript, marking them in red.

**Reviewer #3:** The authors have investigated and reported on the effects of PAK4 protein degraders on bladder cancer.

Several main mechanisms of action have been investigated:

・Blocking the ERK1/2/CDK4 signaling pathway

・Cell cycle arrest in the G1 phase

・Induction of apoptosis

The above have been mentioned, but there are likely many other pathways that affect PAK4 protein.

The following pathways have also been reported, so why not consider investigating them?

<https://pmc.ncbi.nlm.nih.gov/articles/PMC9799984>

<https://pmc.ncbi.nlm.nih.gov/articles/PMC12625063>

Ans: We thank the reviewer for the insightful suggestions. We downloaded and carefully reviewed these two research articles on PAK4. We sincerely apologize for not conducting a comprehensive literature search when reviewing PAK4 research progress. We have now thoroughly read both articles and cited them in the “Introduction” section.

In our study, we focused on the effects of PpD on cell cycle and apoptosis in BLCA, mainly due to the primary rationale stems from the RNA-seq results shown in Fig 2b and 2c. The TOP 10 KEGG and TOP 10 REACTOME pathway analyses indicated significant enrichment of cell cycle and apoptosis-related pathways.

Changes in the text:

---p21-Activated kinase 4 (PAK4), which belongs to the serine/threonine (Ser/Thr) protein kinase family, is overexpressed in many human cancers and is related to poor prognosis, including in BLCA[17-20]. **(see page 4, line 66--68.)**

---Abril-Rodríguez, Gabriel et al. reported that PAK4 inhibition enhances anti-PD-1 immunotherapy efficacy by reversing immune cell exclusion through increased infiltration of CD8+ T cells and CD103+ dendritic cells, mediated by remodeling of the extracellular matrix and upregulation of CCL21-CXCL10 chemokine signaling[26]. Thus, PAK4 is considered a new diagnostic, and a mechanistic rationale for combining PAK4 kinase inhibitors with immune checkpoint blockade for BLCA therapy. **(see page 4, line 72--78.)**
