# Supplementary material for: PAK4-targeting PROTAC inhibits bladder cancer cell proliferation
Source: PLoS One. 2026 Sep 21;21(9):e0358561. doi: 10.1371/journal.pone.0358561 (PMC13592572; doi:10.1371/journal.pone.0358561)
Supplement: Supplementary file 4 [file pone.0358561.s005.docx]

**Reviewer #1:** We have no further questions and look forward to the authors making the sequencing data publicly available.

Ans: We thank the reviewer for the kind comments. Raw data, including sequencing data, mice data, were available at Figshare (https://doi.org/10.6084/m9.figshare.31968720).

**Reviewer #3:** The authors present "PAK4-Targeting PROTAC Inhibits Bladder Cancer Cell Proliferation," focusing on a novel peptide-based degrader, PpD. While the application of PROTAC technology to PAK4 in the context of bladder cancer is of interest, the manuscript in its current form suffers from several weaknesses that limit its impact and translational relevance.

Major points:

1:The authors claim that high PAK4 expression is "positively associated with an increased risk of disease progression" and predicts "poor prognosis". However, a close inspection of the Kaplan-Meier curves in Figure 1c and 1d suggests that the log-rank p-values (p=0.072 and p=0.5) do not reach standard statistical significance (p < 0.05). Overstating these findings is misleading. The authors must either provide a more robust cohort analysis or significantly temper their conclusions regarding PAK4 as a definitive prognostic biomarker in BLCA.

Ans: Thank the reviewer for this critical and insightful comment. We fully agreed with the reviewer that the log-rank P values in Figure 1c and 1d (P=0.072 and P=0.5) do not reach the conventional statistical significance threshold of P < 0.05. We have therefore revised and substantially tempered our conclusions throughout the manuscript. We have removed the overstated claims such as “positively associated with an increased risk of disease progression” and “poor prognosis”. Instead, we have softened the description to “showed a trend toward worse progression and survival”, and clearly stated that the correlation did not reach statistical significance. We have also added this limitation explicitly in the discussion section, acknowledging that the prognostic value of PAK4 in BLCA needs further validation in larger independent clinical cohorts. We apologize for the overinterpretation in the original manuscript and have carefully modified all related statements to be more rigorous and objective.

Changes in the text:

---p21-Activated kinase 4 (PAK4) is widely dysregulated and acts as an oncogene in multiple cancers **(see page 2, line 21-22.)**

---Collectively, our findings indicate that PpD exhibits potent anti-tumor activity against BLCA and holds promising potential for therapeutic application in other cancers.**(see page 2, line 38-40.）**

---The HR values (1.6 and 1.2, respectively) indicated a trend toward worse disease-free and overall survival in patients with high PAK4 expression, although the differences did not reach statistical significance (Fig 1c, d). These observations suggest a potential involvement of PAK4 in BLCA pathogenesis.**(see page 11, line 231-235.）**

---PpD shows potential for the treatment of metastatic kidney cancer. Experts from the Memorial Sloan Kettering Cancer Center have lauded PpD in a dedicated review published in the journal *eBioMedicine*, highlighting its prospective clinical value in future combinatorial oncology treatments[30]**(see page 23, line 500-503.）**

---From the perspective of mechanistic rationale, inhibition of PAK4 activity may constrain the growth and progression of PAK4-high tumors.**(see page 24, line 504-505.）**

---In the present study, we verified that PAK4 is highly expressed in BLCA tissues and tends to correlate with unfavorable clinical outcomes. **(see page 24, line 507-508.）**

---Of note, high PAK4 expression showed only a trend toward association with increased disease progression risk in BLCA patients; notably, the Kaplan–Meier survival analysis failed to reach the conventional statistical significance threshold (p < 0.05) (Fig 1).**(see page 24, line 512-515.）**

---PpD is expected to exhibit potential superior anti-tumor activity in the clinical extermination of BLCA cells.**(see page 25, line 531-532.）**

---In conclusion, our study validates the anti-tumor role and therapeutic potential of PpD in BLCA, expands its applicable research scope, and provides a novel preclinical reference for combination treatment of BLCA. The present findings offer meaningful mechanistic insights and preclinical translational implications for the management of solid tumors.**(see page 27, line 572-576.）**

2:The study evaluates PpD in isolation or against its own negative controls (Se-mutation). To demonstrate the therapeutic value of this PROTAC, it is essential to include a comparator group treated with a standard-of-care agent for bladder cancer, such as Cisplatin or Gemcitabine. Without this comparison, it is impossible to determine if PpD offers any clinical advantage over existing, more affordable chemotherapy regimens.

Ans: We fully agreed with the your valuable suggestion. To further validate the therapeutic potential of PpD and compare its efficacy with clinically approved chemotherapeutic agents for bladder cancer, we have supplemented additional in vivo animal experiments using cisplatin. In the revised manuscript, we added a cisplatin monotherapy group as well as a PpD combined with cisplatin group.

Changes in the text:

---To compare PpD and cisplatin in an in vivo experiment, 20 BALB/c 4-week-old nude mice (female) were randomly separated into 4 groups (5 mice/group), and 2×10^6^ T-24 cells were subcutaneously injected into the right shoulder as previously described. After 6 days, the mice were treated with control (PBS), or PpD 2.5 mg/kg, or cisplatin 2.5 mg/kg, or the combined regimen of PpD and cisplatin (alternating administration: PpD followed by cisplatin) every 2 days via abdominal injection. Tumor size, tumor volume, and mouse body weight were measured every 3 days. Tumor volume was calculated using the following equation: tumor volume=length×width×0.5. **(see page 10, line 197-205.）**

---To compare the anti-tumor efficacy of PpD with cisplatin, a first-line clinical agent for BLCA, we evaluated monotherapies and combination therapy in a xenograft model. As illustrated in Figure 5g, treatment with PpD, or cisplatin led to a substantial decrease in tumor volume relative to the control. As showed in Fig.5h, tumor weights were significantly reduced in all treatment groups compared to control group (PpD, p<0.05; cisplatin, p<0.05; combined, p<0.001), with the combination group showing significantly lower tumor weights than cisplatin monotherapy (p<0.05), while no significant difference was observed between the two monotherapies. All mice were well-tolerated, with no significant body weight loss in any group throughout the study (Fig.5i). Consistent with the Fig.5h results, the control group exhibited progressive tumor growth over 30 days (Fig.5j), whereas all treatments markedly suppressed tumor volume expansion, with the combination therapy exerting the most potent inhibitory effect (p<0.05, p<0.001 compared with control). **(see page 21, line 437-450.）**

---(g) Representative photographs of excised tumors from BALB/c nude mice bearing tumors at the end of the experiment. in the T-24 control group, PpD treatment group (with 2.5 mg/kg PpD treatment), Cisplatilin treatment group (with 2.5 mg/kg PpD treatment), and PpD + Cisplatin combination group (alternating injection: PpD first, then Cisplatin). (h) At the end of the experiment, tumors from all groups were dissected and weighed. Values are shown as mean ± SD. One-way ANOVA was applied for statistical comparisons. *p<0.05, ***p<0.001. (i) Mouse body weight was measured throughout the experimental period. One-way ANOVA was used for statistical comparison between groups. Error bars show standard deviation (SD); ns denotes no statistical significance. (j) Tumor volume changes of xenografted mice during the 30-day experiment. Group differences were analyzed via one-way ANOVA. Error bars indicate ± SD $\text{n=5}$. *p<0.05, ****p<0.001.**(see page 22-23, line 474-485.）**

3:The introduction and discussion emphasize PAK4's role in immune evasion and its potential to enhance immunotherapy. However, the in vivo experiments were conducted in BALB/c nude mice, which lack a functional T-cell-mediated immune system. This model is fundamentally incapable of validating the drug's effect on the tumor immune microenvironment. The authors should either perform experiments in an immunocompetent syngeneic model or remove the extensive speculation regarding immunotherapy enhancement.

Ans: Thank you very much for pointing out this critical issue. We completely agreed with your comment. We recognized that the *in vivo* experiments in this study were performed in BALB/c nude mice, which are deficient in functional T-cell-mediated immunity. Therefore, this nude mouse model is not suitable for validating the regulatory effect of PpD on the tumor immune microenvironment or its potential to boost immunotherapy efficacy.

In response to your suggestion, we have carefully revised relevant statements in both the Introduction and Discussion sections.

Changes in the text:

---However, the immunomodulatory and immunotherapy-synergistic effects of PAK4 targeting in BLCA require further validation in immunocompetent preclinical models. **(see page 4, line 77--79.）**

---However, such immunotherapeutic synergies cannot be extrapolated to the current BLCA study due to the limitation of the animal model used. **(see page 24, line 509-510.）**

---However, given that our in vivo experiments were performed in BALB/c nude mice with deficient T-cell-mediated immune function, we cannot draw definitive conclusions regarding the regulatory effect of PAK4 ablation or inhibition on the infiltration of tumoricidal immune cells.Validation in immunocompetent syngeneic models is required in future work. **(see page 26, line 551-555.）**

4:The authors attribute the effects of PpD almost exclusively to the ERK1/2/CDK4 pathway based on RNA-seq enrichment. Given that PAK4 is known to interact with various other critical pathways such as STAT3, PI3K/AKT, and Wnt/β-catenin, the current mechanistic analysis feels superficial. A more comprehensive Western blot analysis of these alternative pathways is necessary to confirm that the observed phenotype is not merely a localized effect on the cell cycle.

Ans: Thank you for pointing out this limitation in our mechanistic exploration. We fully agreed that PAK4 is closely associated with STAT3, PI3K/AKT and Wnt/β-catenin pathways, and our current mechanistic analysis is relatively focused on the cell cycle-related ERK1/2/CDK4 pathway based on RNA-seq enrichment results. We investigated the phosphorylation status of β-catenin, a component in the signaling pathway downstream of PAK4, following PpD treatment. The results demonstrated that PpD treatment effectively inhibits downstream signaling, as shown in Figure S7a&b . These findings are detailed in our prior publication [reference 27].

Changes in the text:

---Based on RNA-seq enrichment analysis, we focused our mechanistic investigation on the ERK1/2/CDK4 pathway. Considering that PAK4 is a versatile signaling hub that modulates multiple biological cascades including STAT3, PI3K/AKT and Wnt/β-catenin, we further explored the regulatory effect of PpD on these pathways. Our previous findings results confirmed that PpD markedly inhibits the activation β-catenin[27]. This evidence demonstrates that the biological phenotypes induced by PpD are not merely localized alterations in cell cycle progression, but arise from the combined regulation of multiple PAK4 downstream signaling pathways. Future studies will systematically investigate the crosstalk between these signaling axes and their collective functions in PpD-mediated biological effects. **(see page 25, line 532-542.）**

Response figure1:. PpD derectly decreases PAK4’s dowmstream proteins level. (a, b) Western blotting assay analysis of PAK4, β-catenin, and β-catenin^Ser675^ protein levels in 786-O and Caki-1 cell lines treated with indicated concentrations of PpD for 48 hours.

5:The discussion lacks a critical evaluation of the pharmacokinetic (PK) challenges inherent to peptide-based drugs, such as metabolic stability and membrane permeability. Peptide degraders often face high clearance rates in vivo compared to small-molecule PROTACs. The authors should provide preliminary data or a more rigorous discussion on the stability of PpD in serum to support its feasibility as a systemic clinical candidate.

Ans: We thank the reviewer for this insightful and important comment. We fully agree that peptide-based degraders generally face intrinsic pharmacokinetic limitations, including susceptibility to proteolytic degradation, poor passive membrane permeability, short plasma half-life, and rapid systemic clearance. These limitations are particularly relevant when peptide degraders are administered as free peptides.

In the present study, however, PpD was not developed as a naked peptide drug. Instead, we employed a nano-selenium-based delivery strategy to improve the drug-like properties of PpD. This nano-selenium formulation was designed to protect PpD from rapid enzymatic degradation, improve cellular uptake, and partially overcome the membrane permeability limitations commonly associated with peptide therapeutics. Therefore, the feasibility of PpD as a systemic antitumor agent should be considered in the context of this nano-delivery system rather than as a free peptide alone.

To address the reviewers’ concern, we further evaluated the serum stability of nano-selenium-delivered PpD. As shown in the newly added data S2 Fig, PpD exhibited a gradual time-dependent degradation profile in serum rather than rapid loss of intact peptide. Importantly, the calculated serum half-life of PpD was 10.97 h, indicating that the nano-selenium delivery system substantially prolonged the apparent serum stability of PpD and protected it from immediate degradation in the serum environment. These data support the feasibility of nano-selenium-delivered PpD for systemic in vivo proof-of-concept studies.

Changes in the text:

---Meanwhile, PpD exhibited a gradual time-dependent degradation profile in serum , the calculated serum half-life of PpD was 10.97 h, indicating that the nano-selenium delivery system substantially prolonged the apparent serum stability of PpD and protected it from immediate degradation in the serum environment. **(see page 26, line 564-567.）**

Minor Points:

1:・Consistency in Nomenclature: The manuscript inconsistently uses "Bca" and "BLCA" for bladder cancer.

Ans: We appreciate your reminding and apologize for two abbreviation for bladder cancer. In our manuscript R1, we have changed “Bca” to “BLCA”, the text all in red.

2:・Figure Gating: In Figure 2f and 3g, some percentages in the flow cytometry quadrants appear to contain typographical errors or do not sum to 100% exactly, which raises concerns about data processing.

Ans: We greatly appreciated this valuable observation.We confirmd there are no mistakes in the data. During plotting, FlowJo counted the values are rounded to three significant figures (Q4: the lower-left quadrant, or the Q2: the upper-right quadrant), so the rounded displayed values result in a total slightly different from 100% (example: Response figure 2). All raw data and calculation results were reliable.

To avoid misunderstanding, we have regenerated the figures with additional decimal places. The quadrant percentages now sum precisely to 100%. The revised figures have been uploaded accordingly.

Response figure 2
