# Supplementary material for: PAK4-targeting PROTAC inhibits bladder cancer cell proliferation
Source: PLoS One. 2026 Sep 21;21(9):e0358561. doi: 10.1371/journal.pone.0358561 (PMC13592572; doi:10.1371/journal.pone.0358561)
Supplement: Supplementary file 5 [file pone.0358561.s007.docx]

Dear Editor,

We are writing to sincerely apologize for the wrong arrangement of Figure 4A in the submission. When assembling the Western blot figures, the PAK4 and Vinculin blots of T-24 and 5637 cells were incorrectly exchanged by mistake.

We have fixed this problem thoroughly in the R3 revised manuscript by uploading the corrected Figure 4. No other contents in the submission system have been adjusted apart from Figure 4. We appreciate your careful review and kind reminder.

Sincerely,

Bohan Ma Ph.D.

Department of Urology, The First Affiliated Hospital of Xi'an Jiaotong University

Xi'an, P. R. China. 710061, China

E-mail: bohanma@xjtu.edu.cn
