# Supplementary material for: PAK4-targeting PROTAC inhibits bladder cancer cell proliferation
Source: PLoS One. 2026 Sep 21;21(9):e0358561. doi: 10.1371/journal.pone.0358561 (PMC13592572; doi:10.1371/journal.pone.0358561)
Supplement: Peer review history

# Peer review: Decision Letter 0

**DOI:** 10.1371/journal.pone.0358561.r001

**Authors:** Kota Ramana

16 Feb 2026

Dear Dr. XU,

Thank you for submitting your manuscript to PLOS ONE. After careful consideration, we feel that it has merit but does not fully meet PLOS ONE’s publication criteria as it currently stands. Therefore, we invite you to submit a revised version of the manuscript that addresses the points raised during the review process.

Please submit your revised manuscript by Apr 02 2026 11:59PM. If you will need more time than this to complete your revisions, please reply to this message or contact the journal office at plosone@plos.org. When you're ready to submit your revision, log on to https://www.editorialmanager.com/pone/ and select the 'Submissions Needing Revision' folder to locate your manuscript file.

- A letter that responds to each point raised by the academic editor and reviewer(s). You should upload this letter as a separate file labeled 'Response to Reviewers'.
- A marked-up copy of your manuscript that highlights changes made to the original version. You should upload this as a separate file labeled 'Revised Manuscript with Track Changes'.
- An unmarked version of your revised paper without tracked changes. You should upload this as a separate file labeled 'Manuscript'.

If you would like to make changes to your financial disclosure, please include your updated statement in your cover letter. Guidelines for resubmitting your figure files are available below the reviewer comments at the end of this letter.

If applicable, we recommend that you deposit your laboratory protocols in protocols.io to enhance the reproducibility of your results. Protocols.io assigns your protocol its own identifier (DOI) so that it can be cited independently in the future. For instructions see: https://journals.plos.org/plosone/s/submission-guidelines#loc-laboratory-protocols. Additionally, PLOS ONE offers an option for publishing peer-reviewed Lab Protocol articles, which describe protocols hosted on protocols.io. Read more information on sharing protocols at https://plos.org/protocols?utm_medium=editorial-email&utm_source=authorletters&utm_campaign=protocols.

We look forward to receiving your revised manuscript.

Kind regards,

Kota V Ramana, Ph.D.

Academic Editor

PLOS One

**Journal Requirements:**

1. When submitting your revision, we need you to address these additional requirements.

Please ensure that your manuscript meets PLOS ONE's style requirements, including those for file naming. The PLOS ONE style templates can be found at

https://journals.plos.org/plosone/s/file?id=wjVg/PLOSOne_formatting_sample_main_body.pdf and

https://journals.plos.org/plosone/s/file?id=ba62/PLOSOne_formatting_sample_title_authors_affiliations.pdf

2. To comply with PLOS One submissions requirements, in your Methods section, please provide additional information regarding the experiments involving animals and ensure you have included details on (a) methods of sacrifice, (b) methods of anesthesia and/or analgesia, and (c) efforts to alleviate suffering.

3. Thank you for stating in your Funding Statement:

This work was supported by the National Natural Science Foundation of China [82373103, Shan Xu] and Research Funds on Social Development from the Department of Science and Technology of Shaanxi Province of China (grant numbers 2024SF-YBXM-192, Xiaojing Bai).

Please provide an amended statement that declares *all* the funding or sources of support (whether external or internal to your organization) received during this study, as detailed online in our guide for authors at http://journals.plos.org/plosone/s/submit-now.  Please also include the statement “There was no additional external funding received for this study.” in your updated Funding Statement.

Please include your amended Funding Statement within your cover letter. We will change the online submission form on your behalf.

4. Thank you for stating the following financial disclosure:

This work was supported by the National Natural Science Foundation of China [82373103, Shan Xu] and Research Funds on Social Development from the Department of Science and Technology of Shaanxi Province of China (grant numbers 2024SF-YBXM-192, Xiaojing Bai).

Please state what role the funders took in the study.  If the funders had no role, please state: "The funders had no role in study design, data collection and analysis, decision to publish, or preparation of the manuscript."

If this statement is not correct you must amend it as needed.

Please include this amended Role of Funder statement in your cover letter; we will change the online submission form on your behalf.

5. When completing the data availability statement of the submission form, you indicated that you will make your data available on acceptance. We strongly recommend all authors decide on a data sharing plan before acceptance, as the process can be lengthy and hold up publication timelines. Please note that, though access restrictions are acceptable now, your entire data will need to be made freely accessible if your manuscript is accepted for publication. This policy applies to all data except where public deposition would breach compliance with the protocol approved by your research ethics board. If you are unable to adhere to our open data policy, please kindly revise your statement to explain your reasoning and we will seek the editor's input on an exemption. Please be assured that, once you have provided your new statement, the assessment of your exemption will not hold up the peer review process.

6. Your ethics statement should only appear in the Methods section of your manuscript. If your ethics statement is written in any section besides the Methods, please move it to the Methods section and delete it from any other section. Please ensure that your ethics statement is included in your manuscript, as the ethics statement entered into the online submission form will not be published alongside your manuscript.

7. Please include captions for your Supporting Information files at the end of your manuscript, and update any in-text citations to match accordingly. Please see our Supporting Information guidelines for more information: http://journals.plos.org/plosone/s/supporting-information.

8. PLOS ONE now requires that authors provide the original uncropped and unadjusted images underlying all blot or gel results reported in a submission’s figures or Supporting Information files. This policy and the journal’s other requirements for blot/gel reporting and figure preparation are described in detail at https://journals.plos.org/plosone/s/figures#loc-blot-and-gel-reporting-requirements and https://journals.plos.org/plosone/s/figures#loc-preparing-figures-from-image-files. When you submit your revised manuscript, please ensure that your figures adhere fully to these guidelines and provide the original underlying images for all blot or gel data reported in your submission. See the following link for instructions on providing the original image data: https://journals.plos.org/plosone/s/figures#loc-original-images-for-blots-and-gels.

In your cover letter, please note whether your blot/gel image data are in Supporting Information or posted at a public data repository, provide the repository URL if relevant, and provide specific details as to which raw blot/gel images, if any, are not available. Email us at plosone@plos.org if you have any questions.

9. If the reviewer comments include a recommendation to cite specific previously published works, please review and evaluate these publications to determine whether they are relevant and should be cited. There is no requirement to cite these works unless the editor has indicated otherwise. 

[Note: HTML markup is below. Please do not edit.]

Reviewers' comments:

**Comments to the Author**

1. Is the manuscript technically sound, and do the data support the conclusions?

Reviewer #1: Yes

Reviewer #2: No

Reviewer #3: Yes

**********

2. Has the statistical analysis been performed appropriately and rigorously?

Reviewer #1: Yes

Reviewer #2: Yes

Reviewer #3: Yes

**********

3. Have the authors made all data underlying the findings in their manuscript fully available?

The PLOS Data policy

Reviewer #1: Yes

Reviewer #2: Yes

Reviewer #3: Yes

**********

4. Is the manuscript presented in an intelligible fashion and written in standard English?

Reviewer #1: Yes

Reviewer #2: Yes

Reviewer #3: Yes

**********

**Reviewer #1:**  The study presents a promising investigation into a novel PROTAC-based therapeutic strategy for bladder cancer (BCa). The research question is relevant, and the experimental design is generally logical, progressing from target validation to in vivo proof-of-concept. The manuscript is clearly written. However, several major concerns must be addressed to strengthen the scientific rigor, mechanistic insight, and overall impact of the work before it can be considered for publication. My primary recommendations are outlined below.

1. In Figure 1c and 1d, the authors highlight Hazard Ratios (HR=1.6 and 1.2) as "statistically significant" for linking high PAK4 expression to poor survival. However, the presented Kaplan-Meier curves and associated log-rank p-values do not appear to reach statistical significance. The claim of a direct link to poor prognosis cannot stand without statistically robust support.

2. To establish causality, key experiments are needed. For example, does constitutive activation of MEK/ERK or overexpression of Cyclin D1/CDK4 rescue the cell cycle arrest and growth inhibition caused by PpD treatment or PAK4 knockout?

3. The study lacks a comparator group treated with a standard-of-care agent (e.g., cisplatin) or a known PAK4 inhibitor. This makes it impossible to assess the relative efficacy of PpD.

4. Only a single dose of PpD (2.5 mg/kg) was tested. A dose-ranging study is needed to determine the optimal effective dose and potential toxicity.

5. Please provide immunohistochemistry or western blot data from excised tumors to show PAK4 degradation, reduced p-ERK/CDK4, and increased apoptosis (e.g., cleaved caspase-3 staining) in the PpD-treated group compared to the control.

6. The abbreviation for bladder cancer is inconsistently used (“BLCA” in figures and database contexts, “Bca” in the text). Please choose one (e.g., BCa) and use it consistently throughout the manuscript for clarity.

7. More critically discuss the potential advantages and challenges of the PROTAC approach compared to traditional PAK4 kinase inhibitors.

**Reviewer #2:** The pivotal role of P21-activated kinase (PAK) in tumorigenesis and progression has garnered increasing attention. Among these, PAK4 mediates multiple biological behaviors at the cellular level, including tumor initiation, proliferation, metastasis, drug resistance, and immune evasion. PAK4 serves as a predictive, effective, and broadly applicable biomarker and therapeutic target for tumors. Several PAK4 inhibitors have demonstrated therapeutic efficacy in animal studies.

The paper focuses on PpD, a peptide-protein hydrolysis-targeted chimeric drug independently developed by the authors' team. Through cellular and animal models, it validates PpD's role in degrading PAK4 and its therapeutic efficacy in bladder tumors.

The paper presents a logically closed framework with detailed and appropriate methodology, employing validation across multiple levels and perspectives to yield credible conclusions. It may offer new therapeutic options for bladder tumors in the future.

The paper is now in a highly mature state. The following is intended solely for discussion among peers and does not affect the overall evaluation of the full paper.

1. Figure 1a indicates that PAK4 mRNA expression levels are higher in many other tumor tissues than in BLCA. The study currently employs only T-24 and 5637 cells as cellular models. Therefore, would it be more comprehensive to add renal carcinoma cells as positive controls and smooth muscle cells as negative controls, as suggested in the introduction?

If further experimental conditions are constrained or effective working time is limited, does PpD's effect on PAK4 degradation observed in Figure 1a also hold true in tumor cells beyond bladder tumors? What is the current status of research progress?

In other words, what is the distribution pattern of PAK4 in normal tissues? If PpD were to be applied clinically, what potential effects might it have on these numerous normal tissues containing the target? What is the authors' perspective on this?

2. Figure 2f，T24 sg-control group: 4.22% + 5.12% + 90.03% + 0.45% = 99.82%，

Figure 3g，5637 62 nM grou: 3.74% + 7.05% + 0.94% + 88.5% = 100.23%，

If this is not a clerical error, what could cause such discrepancies? Does this indicate abnormal cell status within this batch during experimentation? What is the author's perspective on this?

3. Line 269: “this effect was concentration dependent.”

However, as shown in Figure 3f, the proportion of cells in the G1 phase peaked at 125 nM PpD for both cell types, particularly in the 5637 cells.

4. Line 306: “However, we did not detect any changes in caspase-3 or the cleavage of the PARP protein in the T-24 and 5637 cell lines in which PAK4 was knocked out.”

Since caspase-3 and PARP protein cleavage were already detected in the PAK4-knockout T-24 and 5637 cells, why are these results not presented in Figure 4a? Additionally, the vertical axes of Figure 4a and 4b should ideally be uniformly arranged and correspond to each other to facilitate comparison between figures and alignment with the corresponding results section in the paper.

5. Line 317 lists n=10, whereas Figure 5a shows n=5. This may be a typographical error, please verify.

**Reviewer #3:**  The authors have investigated and reported on the effects of PAK4 protein degraders on bladder cancer.

Several main mechanisms of action have been investigated:

・Blocking the ERK1/2/CDK4 signaling pathway

・Cell cycle arrest in the G1 phase

・Induction of apoptosis

The above have been mentioned, but there are likely many other pathways that affect PAK4 protein.

The following pathways have also been reported, so why not consider investigating them?

https://pmc.ncbi.nlm.nih.gov/articles/PMC9799984

https://pmc.ncbi.nlm.nih.gov/articles/PMC12625063

**********

what does this mean?). If published, this will include your full peer review and any attached files.

If you choose “no”, your identity will remain anonymous but your review may still be made public.

**Do you want your identity to be public for this peer review?**  For information about this choice, including consent withdrawal, please see our Privacy Policy

Reviewer #1: No

Reviewer #2: No

Reviewer #3: No

**********

[NOTE: If reviewer comments were submitted as an attachment file, they will be attached to this email and accessible via the submission site. Please log into your account, locate the manuscript record, and check for the action link "View Attachments". If this link does not appear, there are no attachment files.]

To ensure your figures meet our technical requirements, please review our figure guidelines: https://journals.plos.org/plosone/s/figures

You may also use PLOS’s free figure tool, NAAS, to help you prepare publication quality figures: https://journals.plos.org/plosone/s/figures#loc-tools-for-figure-preparation.

NAAS will assess whether your figures meet our technical requirements by comparing each figure against our figure specifications.

# Author comment: Author response to Decision Letter 1

**DOI:** 10.1371/journal.pone.0358561.r002

9 Apr 2026

We greatly appreciate reviewers’ recommendations, we made a point-to-point revise, please check the "Response to reviewers" document.

Attachment Submitted filename: Response to Reviewers.docx

# Peer review: Decision Letter 1

**DOI:** 10.1371/journal.pone.0358561.r003

**Authors:** Kota Ramana

27 Apr 2026

Dear Dr. XU,

Thank you for submitting your manuscript to PLOS ONE. After careful consideration, we feel that it has merit but does not fully meet PLOS ONE’s publication criteria as it currently stands. Therefore, we invite you to submit a revised version of the manuscript that addresses the points raised during the review process.

Please submit your revised manuscript by Jun 11 2026 11:59PM. If you will need more time than this to complete your revisions, please reply to this message or contact the journal office at plosone@plos.org. When you're ready to submit your revision, log on to https://www.editorialmanager.com/pone/ and select the 'Submissions Needing Revision' folder to locate your manuscript file.

- A letter that responds to each point raised by the academic editor and reviewer(s). You should upload this letter as a separate file labeled 'Response to Reviewers'.
- A marked-up copy of your manuscript that highlights changes made to the original version. You should upload this as a separate file labeled 'Revised Manuscript with Track Changes'.
- An unmarked version of your revised paper without tracked changes. You should upload this as a separate file labeled 'Manuscript'.

If you would like to make changes to your financial disclosure, please include your updated statement in your cover letter. Guidelines for resubmitting your figure files are available below the reviewer comments at the end of this letter.

If applicable, we recommend that you deposit your laboratory protocols in protocols.io to enhance the reproducibility of your results. Protocols.io assigns your protocol its own identifier (DOI) so that it can be cited independently in the future. For instructions see: https://journals.plos.org/plosone/s/submission-guidelines#loc-laboratory-protocols. Additionally, PLOS ONE offers an option for publishing peer-reviewed Lab Protocol articles, which describe protocols hosted on protocols.io. Read more information on sharing protocols at https://plos.org/protocols?utm_medium=editorial-email&utm_source=authorletters&utm_campaign=protocols.

As the corresponding author, your ORCID iD is verified in the submission system and will appear in the published article. PLOS supports the use of ORCID, and we encourage all coauthors to register for an ORCID iD and use it as well. Please encourage your coauthors to verify their ORCID iD within the submission system before final acceptance, as unverified ORCID iDs will not appear in the published article. *Only*  the individual author can complete the verification step; PLOS staff *cannot*  verify ORCID iDs on behalf of authors.

We look forward to receiving your revised manuscript.

Kind regards,

Kota V Ramana, Ph.D.

Academic Editor

PLOS One

Journal Requirements:

If the reviewer comments include a recommendation to cite specific previously published works, please review and evaluate these publications to determine whether they are relevant and should be cited. There is no requirement to cite these works unless the editor has indicated otherwise.

[Note: HTML markup is below. Please do not edit.]

Reviewers' comments:

Reviewer's Responses to Questions

**Comments to the Author**

Reviewer #1: All comments have been addressed

Reviewer #3: (No Response)

**********

2. Is the manuscript technically sound, and do the data support the conclusions?

Reviewer #1: Yes

Reviewer #3: Partly

**********

3. Has the statistical analysis been performed appropriately and rigorously?

Reviewer #1: Yes

Reviewer #3: No

**********

4. Have the authors made all data underlying the findings in their manuscript fully available?

The PLOS Data policy

Reviewer #1: Yes

Reviewer #3: Yes

**********

5. Is the manuscript presented in an intelligible fashion and written in standard English?

Reviewer #1: Yes

Reviewer #3: Yes

**********

Reviewer #1: We have no further questions and look forward to the authors making the sequencing data publicly available.

Reviewer #3: The authors present "PAK4-Targeting PROTAC Inhibits Bladder Cancer Cell Proliferation," focusing on a novel peptide-based degrader, PpD. While the application of PROTAC technology to PAK4 in the context of bladder cancer is of interest, the manuscript in its current form suffers from several weaknesses that limit its impact and translational relevance.

Major points:

The authors claim that high PAK4 expression is "positively associated with an increased risk of disease progression" and predicts "poor prognosis". However, a close inspection of the Kaplan-Meier curves in Figure 1c and 1d suggests that the log-rank p-values (p=0.072 and p=0.5) do not reach standard statistical significance (p < 0.05). Overstating these findings is misleading. The authors must either provide a more robust cohort analysis or significantly temper their conclusions regarding PAK4 as a definitive prognostic biomarker in BLCA.

The study evaluates PpD in isolation or against its own negative controls (Se-mutation). To demonstrate the therapeutic value of this PROTAC, it is essential to include a comparator group treated with a standard-of-care agent for bladder cancer, such as Cisplatin or Gemcitabine. Without this comparison, it is impossible to determine if PpD offers any clinical advantage over existing, more affordable chemotherapy regimens.

The introduction and discussion emphasize PAK4's role in immune evasion and its potential to enhance immunotherapy. However, the in vivo experiments were conducted in BALB/c nude mice, which lack a functional T-cell-mediated immune system. This model is fundamentally incapable of validating the drug's effect on the tumor immune microenvironment. The authors should either perform experiments in an immunocompetent syngeneic model or remove the extensive speculation regarding immunotherapy enhancement.

The authors attribute the effects of PpD almost exclusively to the ERK1/2/CDK4 pathway based on RNA-seq enrichment. Given that PAK4 is known to interact with various other critical pathways such as STAT3, PI3K/AKT, and Wnt/β-catenin, the current mechanistic analysis feels superficial. A more comprehensive Western blot analysis of these alternative pathways is necessary to confirm that the observed phenotype is not merely a localized effect on the cell cycle.

The discussion lacks a critical evaluation of the pharmacokinetic (PK) challenges inherent to peptide-based drugs, such as metabolic stability and membrane permeability. Peptide degraders often face high clearance rates in vivo compared to small-molecule PROTACs. The authors should provide preliminary data or a more rigorous discussion on the stability of PpD in serum to support its feasibility as a systemic clinical candidate.

Minor Points:

・Consistency in Nomenclature: The manuscript inconsistently uses "Bca" and "BLCA" for bladder cancer.

・Figure Gating: In Figure 2f and 3g, some percentages in the flow cytometry quadrants appear to contain typographical errors or do not sum to 100% exactly, which raises concerns about data processing.

**********

what does this mean?). If published, this will include your full peer review and any attached files.

If you choose “no”, your identity will remain anonymous but your review may still be made public.

**Do you want your identity to be public for this peer review?**  For information about this choice, including consent withdrawal, please see our Privacy Policy

Reviewer #1: No

Reviewer #3: No

**********

[NOTE: If reviewer comments were submitted as an attachment file, they will be attached to this email and accessible via the submission site. Please log into your account, locate the manuscript record, and check for the action link "View Attachments". If this link does not appear, there are no attachment files.]

To ensure your figures meet our technical requirements, please review our figure guidelines: https://journals.plos.org/plosone/s/figures

You may also use PLOS’s free figure tool, NAAS, to help you prepare publication quality figures: https://journals.plos.org/plosone/s/figures#loc-tools-for-figure-preparation.

NAAS will assess whether your figures meet our technical requirements by comparing each figure against our figure specifications.

# Author comment: Author response to Decision Letter 2

**DOI:** 10.1371/journal.pone.0358561.r004

8 Jun 2026

We sincerely thank all reviewers for their thorough evaluation and constructive comments on our manuscript. These suggestions have significantly improved the quality, rigor, and completeness of our work. We have conducted additional experiments—including evaluate the serum stability of nano-selenium-delivered PpD, compare PpD and cisplatin in an in vivo experiment—to address each concern raised by the reviewers. Below, we provide point-by-point responses to all comments. Reviewer comments are shown in black, and our responses follow directly in blue, and the changed text in R2 in red. All figure references correspond to the revised manuscript. We have also submitted a tracked-changes version and a clean version of the revised manuscript.

Attachment Submitted filename: Response to Reviewers R2 .docx

# Editor report: Decision Letter 2

**DOI:** 10.1371/journal.pone.0358561.r005

**Authors:** Kota Ramana

16 Jun 2026

Dear Dr. XU,

Thank you for submitting your manuscript to PLOS ONE. After careful consideration, we feel that it has merit but does not fully meet PLOS ONE’s publication criteria as it currently stands. Therefore, we invite you to submit a revised version of the manuscript that addresses the points raised during the review process.

**Request from the Editorial Office:**

Please submit your revised manuscript by Jul 27 2026 11:59PM. If you will need more time than this to complete your revisions, please reply to this message or contact the journal office at plosone@plos.org. When you're ready to submit your revision, log on to https://www.editorialmanager.com/pone/ and select the 'Submissions Needing Revision' folder to locate your manuscript file.

- A letter that responds to each point raised by the academic editor and reviewer(s). You should upload this letter as a separate file labeled 'Response to Reviewers'.
- A marked-up copy of your manuscript that highlights changes made to the original version. You should upload this as a separate file labeled 'Revised Manuscript with Track Changes'.
- An unmarked version of your revised paper without tracked changes. You should upload this as a separate file labeled 'Manuscript'.

If you would like to make changes to your financial disclosure, please include your updated statement in your cover letter. Guidelines for resubmitting your figure files are available below the reviewer comments at the end of this letter.

If applicable, we recommend that you deposit your laboratory protocols in protocols.io to enhance the reproducibility of your results. Protocols.io assigns your protocol its own identifier (DOI) so that it can be cited independently in the future. For instructions see: https://journals.plos.org/plosone/s/submission-guidelines#loc-laboratory-protocols. Additionally, PLOS ONE offers an option for publishing peer-reviewed Lab Protocol articles, which describe protocols hosted on protocols.io. Read more information on sharing protocols at https://plos.org/protocols?utm_medium=editorial-email&utm_source=authorletters&utm_campaign=protocols.

As the corresponding author, your ORCID iD is verified in the submission system and will appear in the published article. PLOS supports the use of ORCID, and we encourage all coauthors to register for an ORCID iD and use it as well. Please encourage your coauthors to verify their ORCID iD within the submission system before final acceptance, as unverified ORCID iDs will not appear in the published article. *Only*  the individual author can complete the verification step; PLOS staff *cannot*  verify ORCID iDs on behalf of authors.

We look forward to receiving your revised manuscript.

Kind regards,

Sarah Jose, Ph.D.

Staff Editor

PLOS One

Journal Requirements:

If the reviewer comments include a recommendation to cite specific previously published works, please review and evaluate these publications to determine whether they are relevant and should be cited. There is no requirement to cite these works unless the editor has indicated otherwise.

Please review your reference list to ensure that it is complete and correct. If you have cited papers that have been retracted, please include the rationale for doing so in the manuscript text, or remove these references and replace them with relevant current references. Any changes to the reference list should be mentioned in the rebuttal letter that accompanies your revised manuscript. If you need to cite a retracted article, indicate the article’s retracted status in the References list and also include a citation and full reference for the retraction notice.

[Note: HTML markup is below. Please do not edit.]

[NOTE: If reviewer comments were submitted as an attachment file, they will be attached to this email and accessible via the submission site. Please log into your account, locate the manuscript record, and check for the action link "View Attachments". If this link does not appear, there are no attachment files.]

To ensure your figures meet our technical requirements, please review our figure guidelines: https://journals.plos.org/plosone/s/figures

You may also use PLOS’s free figure tool, NAAS, to help you prepare publication quality figures: https://journals.plos.org/plosone/s/figures#loc-tools-for-figure-preparation.

NAAS will assess whether your figures meet our technical requirements by comparing each figure against our figure specifications.

# Author comment: Author response to Decision Letter 3

**DOI:** 10.1371/journal.pone.0358561.r006

15 Jul 2026

We sincerely thank all reviewers for your careful review and constructive suggestions regarding our manuscript. Your valuable comments have greatly enhanced the rigor, integrity and quality of our research. To resolve all the questions you raised, we have carried out supplementary experiments, including evaluating the serum stability of nano-selenium-loaded PpD and comparing PpD with cisplatin in in vivo assays.

We respond to each comment point by point below. Reviewers’ comments are marked in black, our replies in blue, and all revised content in the second revised manuscript (R2) is highlighted in red. All figure labels correspond to the updated manuscript. We have uploaded both a tracked-changes manuscript and a clean revised manuscript. In accordance with the journal’s image specifications, complete uncropped and unaltered original blot and gel images have been added as supporting information.

# Editor report: Decision Letter 3

**DOI:** 10.1371/journal.pone.0358561.r007

**Authors:** Kota Ramana

13 Aug 2026

Dear Dr. XU,

Thank you for submitting your manuscript to PLOS ONE. After careful consideration, we feel that it has merit but does not fully meet PLOS ONE’s publication criteria as it currently stands. Therefore, we invite you to submit a revised version of the manuscript that addresses the following concern.

Found inconsistency in Figure 4A of the submitted original blot images. The inconsistency is listed as below:

4A: sg-PAK4-10 and sg-PAK4-33 seemed exchanged.

Blot for Vinculin exchanged

Provide a clarification for replacing the original blot images.

Please submit your revised manuscript by Oct 11 2026 11:59PM. If you will need more time than this to complete your revisions, please reply to this message or contact the journal office at plosone@plos.org. When you're ready to submit your revision, log on to https://www.editorialmanager.com/pone/ and select the 'Submissions Needing Revision' folder to locate your manuscript file.

- A letter that responds to each point raised by the academic editor and reviewer(s). You should upload this letter as a separate file labeled 'Response to Reviewers'.
- A marked-up copy of your manuscript that highlights changes made to the original version. You should upload this as a separate file labeled 'Revised Manuscript with Track Changes'.
- An unmarked version of your revised paper without tracked changes. You should upload this as a separate file labeled 'Manuscript'.

If you would like to make changes to your financial disclosure, please include your updated statement in your cover letter. Guidelines for resubmitting your figure files are available below the reviewer comments at the end of this letter.

If applicable, we recommend that you deposit your laboratory protocols in protocols.io to enhance the reproducibility of your results. Protocols.io assigns your protocol its own identifier (DOI) so that it can be cited independently in the future. For instructions see: https://journals.plos.org/plosone/s/submission-guidelines#loc-laboratory-protocols. Additionally, PLOS ONE offers an option for publishing peer-reviewed Lab Protocol articles, which describe protocols hosted on protocols.io. Read more information on sharing protocols at https://plos.org/protocols?utm_medium=editorial-email&utm_source=authorletters&utm_campaign=protocols.

As the corresponding author, your ORCID iD is verified in the submission system and will appear in the published article. PLOS supports the use of ORCID, and we encourage all coauthors to register for an ORCID iD and use it as well. Please encourage your coauthors to verify their ORCID iD within the submission system before final acceptance, as unverified ORCID iDs will not appear in the published article. *Only*  the individual author can complete the verification step; PLOS staff *cannot*  verify ORCID iDs on behalf of authors.

We look forward to receiving your revised manuscript.

Kind regards,

Kota V Ramana, Ph.D.

Academic Editor

PLOS One

Journal Requirements:

If the reviewer comments include a recommendation to cite specific previously published works, please review and evaluate these publications to determine whether they are relevant and should be cited. There is no requirement to cite these works unless the editor has indicated otherwise.

Please review your reference list to ensure that it is complete and correct. If you have cited papers that have been retracted, please include the rationale for doing so in the manuscript text, or remove these references and replace them with relevant current references. Any changes to the reference list should be mentioned in the rebuttal letter that accompanies your revised manuscript. If you need to cite a retracted article, indicate the article’s retracted status in the References list and also include a citation and full reference for the retraction notice.

[Note: HTML markup is below. Please do not edit.]

[NOTE: If reviewer comments were submitted as an attachment file, they will be attached to this email and accessible via the submission site. Please log into your account, locate the manuscript record, and check for the action link "View Attachments". If this link does not appear, there are no attachment files.]

To ensure your figures meet our technical requirements, please review our figure guidelines: https://journals.plos.org/plosone/s/figures

You may also use PLOS’s free figure tool, NAAS, to help you prepare publication quality figures: https://journals.plos.org/plosone/s/figures#loc-tools-for-figure-preparation.

NAAS will assess whether your figures meet our technical requirements by comparing each figure against our figure specifications.

# Author comment: Author response to Decision Letter 4

**DOI:** 10.1371/journal.pone.0358561.r008

19 Aug 2026

Dear Editor,

We are writing to sincerely apologize for the wrong arrangement of Figure 4A in the submission. When assembling the Western blot figures, the PAK4 and Vinculin blots of T-24 and 5637 cells were incorrectly exchanged by mistake.

We have fixed this problem thoroughly in the R3 revised manuscript by uploading the corrected Figure 4. No other contents in the submission system have been adjusted apart from Figure 4. We appreciate your careful review and kind reminder.

Sincerely,

Bohan Ma Ph.D.

Department of Urology, The First Affiliated Hospital of Xi'an Jiaotong University

Xi'an, P. R. China. 710061, China

E-mail: bohanma@xjtu.edu.cn

Attachment Submitted filename: Response to Reviewers R3.docx

# Editor report: Decision Letter 4

**DOI:** 10.1371/journal.pone.0358561.r009

**Authors:** Kota Ramana

2 Sep 2026

PAK4-Targeting PROTAC Inhibits Bladder Cancer Cell Proliferation

PONE-D-25-59407R4

Dear Dr. XU,

We’re pleased to inform you that your manuscript has been judged scientifically suitable for publication and will be formally accepted for publication once it meets all outstanding technical requirements.

Within one week, you’ll receive an e-mail detailing the required amendments. When these have been addressed, you’ll receive a formal acceptance letter and your manuscript will be scheduled for publication.

An invoice will be generated when your article is formally accepted. Please note, if your institution has a publishing partnership with PLOS and your article meets the relevant criteria, all or part of your publication costs will be covered. Please make sure your user information is up-to-date by logging into Editorial Manager at Editorial Manager® and clicking the ‘Update My Information' link at the top of the page. For questions related to billing, please contact billing support.

If your institution or institutions have a press office, please notify them about your upcoming paper to help maximize its impact. If they’ll be preparing press materials, please inform our press team as soon as possible -- no later than 48 hours after receiving the formal acceptance. Your manuscript will remain under strict press embargo until 2 pm Eastern Time on the date of publication. For more information, please contact onepress@plos.org.

Kind regards,

Kota V Ramana, Ph.D.

Academic Editor

PLOS One

Additional Editor Comments (optional):

Reviewers' comments:

# Editor report: Acceptance letter

**DOI:** 10.1371/journal.pone.0358561.r010

**Authors:** Kota Ramana

PONE-D-25-59407R4

PLOS One

Dear Dr. XU,

I'm pleased to inform you that your manuscript has been deemed suitable for publication in PLOS One. Congratulations! Your manuscript is now being handed over to our production team.

At this stage, our production department will prepare your paper for publication. This includes ensuring the following:

* All references, tables, and figures are properly cited

* All relevant supporting information is included in the manuscript submission,

* There are no issues that prevent the paper from being properly typeset

You will receive further instructions from the production team, including instructions on how to review your proof when it is ready. Please keep in mind that we are working through a large volume of accepted articles, so please give us a few days to review your paper and let you know the next and final steps.

Lastly, if your institution or institutions have a press office, please let them know about your upcoming paper now to help maximize its impact. If they'll be preparing press materials, please inform our press team within the next 48 hours. Your manuscript will remain under strict press embargo until 2 pm Eastern Time on the date of publication. For more information, please contact onepress@plos.org.

You will receive an invoice from PLOS for your publication fee after your manuscript has reached the completed accept phase. If you receive an email requesting payment before acceptance or for any other service, this may be a phishing scheme. Learn how to identify phishing emails and protect your accounts at https://explore.plos.org/phishing.

If we can help with anything else, please email us at customercare@plos.org.

Thank you for submitting your work to PLOS One and supporting open access.

Kind regards,

PLOS One Editorial Office Staff

on behalf of

Dr. Kota V Ramana

Academic Editor

PLOS One
